# Rationalizing and Adapting Water-Accelerated Reactions for Sustainable Flow Organic Processes

**DOI:** 10.1021/acssuschemeng.3c02164

**Published:** 2023-05-30

**Authors:** Katarzyna A. Maltby, Krishna Sharma, Marc A. S. Short, Sannia Farooque, Rosalie Hamill, A. John Blacker, Nikil Kapur, Charlotte E. Willans, Bao N. Nguyen

**Affiliations:** † Institute of Process Research & Development, School of Chemistry, University of Leeds, Leeds LS2 9JT, U.K.; ‡ School of Mechanical Engineering, University of Leeds, Leeds LS2 9JT, U.K.

**Keywords:** water as a solvent, multiphase flow, on-water reactions

## Abstract

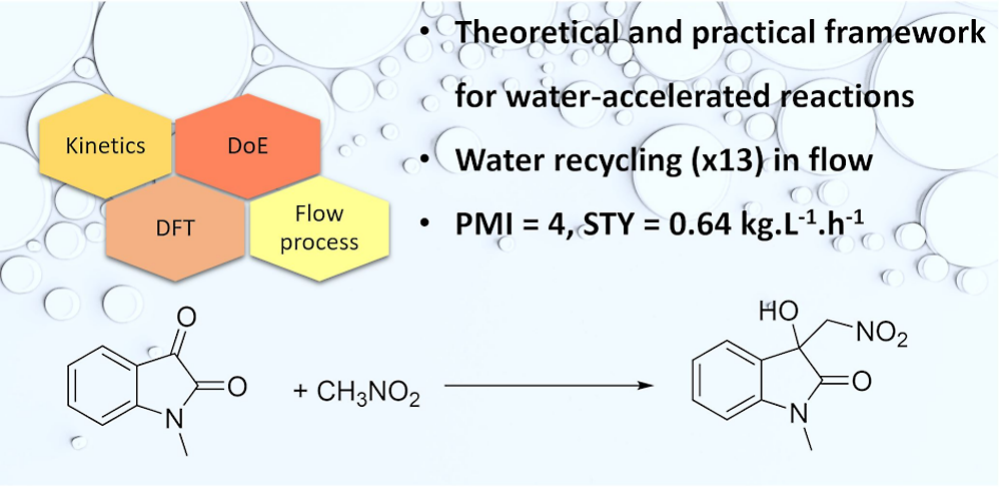

Water-accelerated reactions, wherein at least one organic reactant is not soluble in water, are an important class of organic reactions, with a potentially pivotal impact on sustainability of chemical manufacturing processes. However, mechanistic understanding of the factors controlling the acceleration effect has been limited, due to the complex and varied physical and chemical nature of these processes. In this study, a theoretical framework has been established to calculate the rate acceleration of known water-accelerated reactions, giving computational estimations of the change to Δ*G*^‡^ which correlate with experimental data. In-depth study of a Henry reaction between *N*-methylisatin and nitromethane using our framework led to rationalization of the reaction kinetics, its lack of dependence on mixing, kinetic isotope effect, and different salt effects with NaCl and Na_2_SO_4_. Based on these findings, a multiphase flow process which includes continuous phase separation and recycling of the aqueous phase was developed, and its superior green metrics (PMI-reaction = 4 and STY = 0.64 kg L^–1^ h^–1^) were demonstrated. These findings form the essential basis for further in silico discovery and development of water-accelerated reactions for sustainable manufacturing.

## Abstract 2

A theoretical and practical framework for predicting, rationalizing, and adapting water-accelerated reactions into holistically sustainable flow processes has been demonstrated.

## 1 Introduction

Water is the reaction medium used by nature and has been touted as the ultimate sustainable reaction medium for chemical reactions.^1−3^ Despite the obvious advantages, organic syntheses often avoid water, and significant research effort has been directed at finding acceptable alternative organic solvents to improve sustainability instead. This curious trend can be attributed to poor solubility of the majority of organic compounds in water, incompatibility of traditional reagents with water, and the challenges in treating organic contaminated waste water.^4^ In this context, on-water reactions, i.e., organic reactions which take place as an emulsion in water and exhibit unusual rate acceleration compared to the same reaction in an organic solvent,^5,6^ are particularly attractive. Furthermore, improvements in regio- and stereoselectivity have also been reported in some cases in water in comparison to reactions performed in organic solvents.^6−8^ The advantages of on-water reactions are as follows: (i) they can be carried out in the water medium, even when the reactants are highly insoluble in water; (ii) rate acceleration under on-water conditions can lead to improved space-time-yield (STY); and (iii) simple and efficient purification of the product in high-yielding reactions.

**Figure 1:**
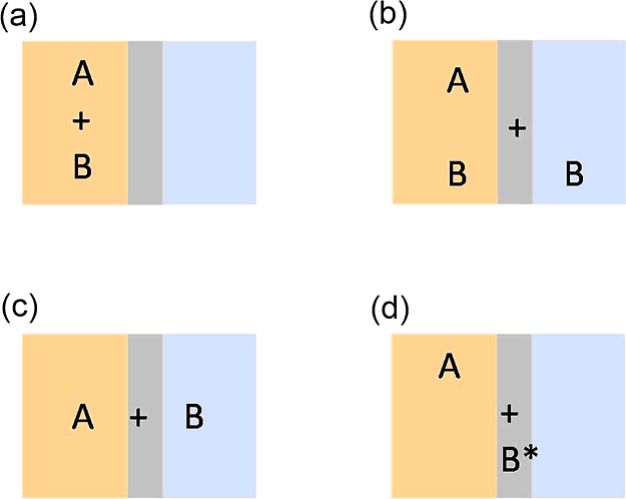
Simplified phase complexity scenarios of water-accelerated reactions: (a) all reactants are insoluble in water; (b) one reactant is partially soluble in water; (c) reactants are phase-separated; and (d) one reactant is activated at the aqueous–organic interface.

Since the early discoveries by Breslow and Sharpless,^5,6^ the past few decades have seen significant growth in the number of on-water reactions, some of which are synthetically useful. These have been well summarized and discussed in numerous reviews.^9−13^ Despite, or perhaps because of, the very wide range of classes of reactions which are known to benefit from the on-water effect, e.g., pericyclic,^14,15^ multicomponent,^16^ nucleophilic ring-opening,^8,17,18^ Mannich,^7^ aldol,^19^ Henry,^20^ Lewis acid-catalyzed,^19^ and organocatalytic reactions,^15,21^ the mechanistic rationalization of their rate acceleration, which may guide the discovery of new on-water reactions, is still in its infancy. This is further confounded by the limited availability of reliable kinetic data^22−24^ and the complex and variable phase behavior of these reactions (Figure 1), which may start off as “in water” and become “on water” as the reaction progresses or vice versa.^25^

The water-acceleration effect has been attributed to the concentration increase in organic droplets,^26^ polarity effect,^27,28^ and stabilization of the transition state at the water–organic interface.^29^ Some of these aspects are established, such as the dependence of the acceleration effect on droplet size/biphasic interfacial area.^30^ Others are less well understood, e.g., stabilization of the transition state by hydrogen bonds in early ab initio/statistical and density functional theory (DFT) studies,^31−34^ and conflicting salt effects.^6,22,23^ In many cases, at least one reactant is partially soluble in water, rendering the strict “on-water reactions” definition invalid and the broader term “water-accelerated reactions”, which will be employed throughout this manuscript, more applicable. These and the lack of a theoretical framework for water-accelerated reactions have been major obstacles in the rational discovery of new and synthetically important reactions and their applications in syntheses.

In this study, we report our comprehensive computational and experimental study of a water-accelerated reaction, leading to a theoretical/practical framework that accounts for both the physical and chemical aspects of this reaction. The results consist of (i) a suitable modern molecular modeling method to study water-accelerated reaction; (ii) the application of such a method for rationalizing conflicting kinetic observations of an example reaction; and (iii) the practical application of mechanistic insights, solubility, and phase behaviors to adapt this example reaction into a multiphase flow process with excellent green metrics by successfully recycling the aqueous phase.

## 2 Experimental Section

All experiments were performed at pH 7, verified by measurements with Mettler Toledo SevenExcellence S400.

### 2.1 Materials

All solvents and reagents were purchased from Sigma-Aldrich UK and used without further purification. Solvents were of HPLC standard.

### 2.2 Standard Protocol for Henry Reaction

The reactions were heated and stirred (1 cm × 1 cm cross-bar stirrer) on a custom-made heating block with 1 in. offset stirring. Reactions were carried out in 4-dram glass vials (2.1 × 7 cm, 14 mL) sealed with a lid (PTFE septum) unless stated otherwise.

Methylisatin **1** (0.0806 g, 0.5 mmol) was added to a 4-dram vial (2.1 × 7 cm) equipped with a cross-bar stirrer (1 cm × 1 cm) followed by nitromethane (812 μL, 15 mmol). After 5 min, deionized water (3 mL) was added, and the sample tube was sealed with a lid and heated to 70 °C at 700 rpm. After 3 h, the reaction mixture was extracted with ethyl acetate (10 mL) to afford the product **2** as pale-yellow oil (0.1077 g, 97%).

### 2.3 Reactions in the Presence of Different Salts

The reactions were performed in deionized water, 1 M NaCl, 1 M LiCl, 1 M Na_2_SO_4_, and 0.1 M phosphate buffer at pH 7. Reactions were done separately, worked up, and analyzed by ^1^H NMR for the kinetic data. Methylisatin **1** (0.0806 g, 0.5 mmol) was added to the 4-dram vials (2.1 × 7 cm) separately equipped with cross-bar stirrers (1 × 1 cm) followed by nitromethane (812 μL, 15 mmol). After 5 min, the relevant aqueous additive (3 mL) was added, and the sample tube was sealed with a lid and heated to 70 °C at 700 rpm. The facile reaction on phosphate buffer was done at room temperature to allow kinetic analysis. After a specific time, the reaction mixtures were extracted with ethylacetate and dried (MgSO_4_), and the solvent was evaporated to afford the product as pale-yellow oil.

### 2.4 Recycling of the Aqueous Phase in Batch

Methylisatin **1** (51 mg; 0.317 mmol) and nitromethane (0.42 mL; 7.925 mmol) were placed in a reaction vial (OD: 2.1 cm; H: 7 cm) with a PTFE septum lid. When methylisatin dissolved fully in nitromethane, 3 mL of water or 0.1 M phosphate buffer at pH 7 was added, and the reaction vial was placed in a custom-made aluminum heating block with 1 in. offset stirring. The reaction was stirred using a magnetic stirrer (1 cm × 1 cm cross-bar stirrer). Each reaction was left for 3 h to react at 70 °C. Due to issues with phase separation, the sample was left at room temperature with no stirring overnight to reach complete separation; then, the aqueous phase was separated using 1 mL plastic syringe with a stainless-steel needle. A sample of the organic phase was dissolved in CDCl_3_ and analyzed via ^1^H NMR using ratios of aromatic signals.

### 2.5 Flow Experiments

Flow experiments were performed in commercially available continuous stirred tank reactors (CSTRs, fReactors, Asynt) with a membrane phase separator (Zaiput SEP-10) using hydrophobic membrane OB-900-S10 (if syringe pumps used) or OB-2000-S10 (piston recirculation pumps). The setup is shown in Figure 6.

The reactants were delivered to the reactors using syringe/syringe pumps with two streams: (i) *N*-methylisatin **1** and trimethoxybenzene (internal standard) dissolved in nitromethane and (ii) 0.1 M aqueous phosphate buffer. PTFE tubing was used for all connections (external diameter 1/8″ and internal diameter 1/16″). A cascade of three mini-CSTR fReactors was used, each fReactor was equipped with a 1 cm × 1 cm cross-bar stirrer, and the overall volume was 4.8 mL (excluding tubing between reactors). Temperature was measured with a thermocouple type J and a handheld temperature reader.

The standard experimental conditions are as follows: 0.48 mL/min overall flow rate (0.24 mL/min flow rate of each phase), 10 min residence time, 40 °C, 850 rpm, 0.121 g of methylisatin, and 6 mg of trimethoxybenzene in 1 mL of nitromethane. Samples for analysis were taken from the organic phase of reaction after phase separation (about 2 drops of the organic phase dissolved in about 0.6 mL of CDCl_3_), and yields/conversions were calculated via ^1^H NMR.

### 2.6 Characterization

Nuclear magnetic resonance (NMR) spectra were recorded for ^1^H at 400 and 500 MHz and ^13^C at 100 and 125 MHz on a Bruker DPX400 or DRX500 spectrometer. A Bruker DRX 500 spectrometer was equipped with a multinuclear inverse probe for one-dimensional ^1^H and two-dimensional heteronuclear single-quantum coherence (^1^H–^13^C HSQC), heteronuclear multiple bond correlation (^1^H–^13^C HMBC), and double quantum filtered correlation (^1^H–^1^H COSY). Chemical shifts (δ) are quoted in ppm downfield of tetramethylsilane or residual solvent peaks (7.26 and 77.16 ppm for CDCl_3_ in ^1^H and ^13^C, respectively). The coupling constants (*J*) are quoted in Hz (multiplicities: s, singlet; bs, broad singlet; d, doublet; t, triplet; and q, quartet, and apparent multiplicities are described as m).

High-resolution mass spectroscopy (HRMS) spectra were recorded on a Dionex Ultimate 3000 spectrometer using electron spray ionization (ESI). All masses quoted are correct to four decimal places. Infrared (IR) spectra were recorded using a PerkinElmer Spectrum One FT-IR spectrophotometer or Bruker Alpha Platinum AR FTIR. Vibrational frequencies are reported in wavenumbers (cm^–1^).

HPLC was carried out on Agilent 1290 infinity series, equipped with a diode-array detector (DAD), binary pump system connected with online degasser, and Zorbax Eclipse XDB C18, 150 × 4.6 mm, 5 μm, column. The flow rate and the injection volume were 1 mL/min and 10 μL, respectively. The chromatograms were recorded by scanning the absorption at 190–600 nm.

## 3 Results and Discussion

**Scheme 1:**
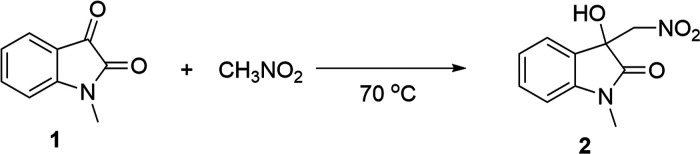


**Figure 2:**
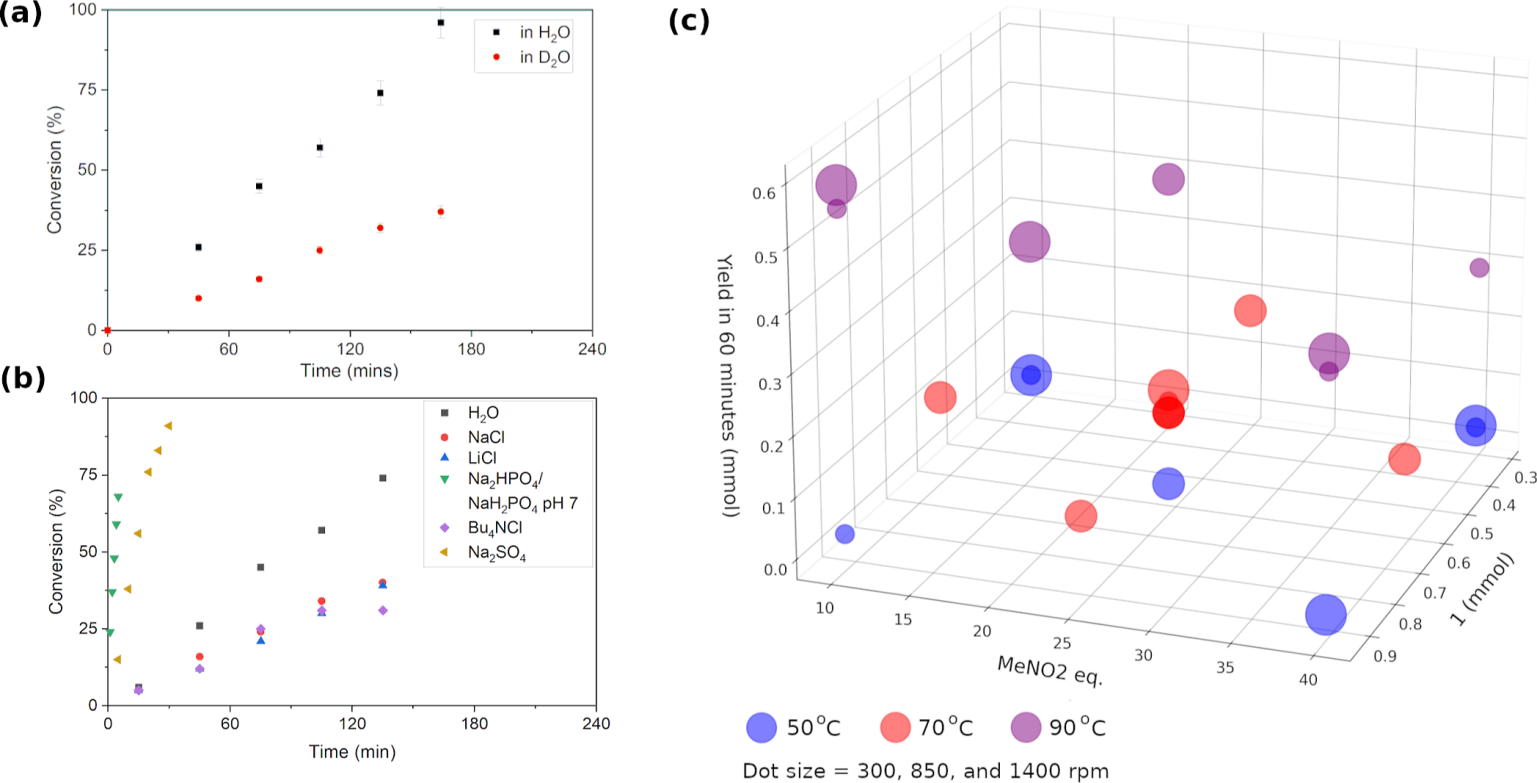
Dependence of the water-accelerated Henry reaction on reaction conditions: (a) kinetic isotope effect; (b) salt effects by NaCl 1 M, LiCl 1 M, Bu_4_NCl 1 M, Na_2_SO_4_ 1 M (70 °C), and phosphate buffer 0.1 M at pH 7 (22 °C), error bars are excluded for clarity; and (c) effect of temperature (color), stirring rate (dot size), excess nitromethane, and amount of **1** on reaction yield at 1 h.

Experimental investigations into solvent and salt effects were performed for a previously published Henry reaction (Table 1, Scheme 1, and Figure 2b).^35^ This reaction was chosen due to its established water-accelerated nature^35^ and its well-understood mechanism in organic solvents. The reaction has a two-step mechanism, partial solubility of one reactant in water, i.e., nitromethane, and a likely enolization step at the organic–water interface [scenario (d) in Figure 1]. Complete consumption of *N*-methylisatin (**1**) was observed in 3 h under water-accelerated conditions when 25 equiv of nitromethane was used. No dehydration product was observed by ^1^H NMR, due to the neutral reaction conditions. The reaction did not proceed in dichloromethane, ethanol, and a mixture of ethanol and water. No reaction was observed when no solvent was added.

**Table 1:** Influence of Reaction Conditions on Henry Reaction^a^^,^^b^

no.	solvent/medium^a^	reaction time (h)	yield (%, ^1^H NMR)
1	none	8	0
2	1,2-dichloroethane	18	0
3	EtOH	18	0
4	EtOH/H_2_O 9:1	18	0
5	H_2_O (pH 7)	3	98

a Typical reaction conditions: *N*-methylisatin (0.317 mmol), nitromethane (7.925 mmol, 25 equiv), solvent (3 mL), 70 °C, and 800 rpm stirring rate with a BRAND crosshead magnetic stirrer (10 mm span), in sealed 14 mL dram vials (OD 22 mm) and custom aluminum heating blocks (see the Supporting Information).

b Measured with a pH probe.

Kinetic profiling of the reaction with ^1^H NMR showed zero-order kinetics in [**1**] with excess nitromethane (Figure 2). When D_2_O was used instead of water, a kinetic isotope effect of  was observed (Figure 2a), indicating that a proton transfer is involved in the rate-determining step (RDS). In contrast to the beneficial hydrophobic effect reported by Breslow with the addition of LiCl salt,^6^ different effects were observed in this study. When NaCl 1 M, LiCl 1 M, or Bu_4_NCl 1 M solutions were used as reaction medium, a common and similar decrease in reaction rate was observed (Figure 2b). This effect can be attributed to the salting-out effect,^36^ i.e., the decrease in solubility of nitromethane in water due to increased ionic strength, which highlights the need for enolization of nitromethane in the aqueous phase. Measurement of solubility of nitromethane at 70 °C by ^1^H NMR showed a decrease from 17.1 ± 0.7% w/w in deionized water to 12.9 ± 0.7% w/w in NaCl 1 M. The presence of phosphate buffer 0.1 M at pH 7 led to >20 times acceleration in reaction rate,^37^ consistent with well-documented phosphate-catalyzed proton transfers.^38,39^ Another salt which showed an unexpected catalytic effect, albeit less pronounced, was Na_2_SO_4_. The only related example in the literature is where a change of selectivity was observed between Na_2_SO_4_ and NaOTs as salt additives reported by Sela and Vigalok in a Passerini-type multicomponent reaction.^40^ Additionally, the solubility of nitromethane in Na_2_SO_4_ 1 M solution showed an even further decrease to 6.8 ± 0.2% w/w without compromising the acceleration of reaction rate. Thus, the observed acceleration of the reaction in Na_2_SO_4_ 1 M compared to deionized water cannot be easily explained.

A design of experiments (DoE) was performed to investigate the importance of stirring, temperature, and reactant amount on the reaction yield in 1 h (Figure 2c). Different stirring rates (300–1400 rpm), 1 in. off-center, did not affect the yield of reaction, in contrast with observations by Huck.^41^ Temperature was found to have the most significant impact on the reaction yield, suggesting that the RDS is chemical in nature rather than mass transfer at the water–organic interface. Molar equivalents of nitromethane did not affect reaction yield at 50 and 70 °C. Observations at 90 °C can be difficult to interpret close to the nitromethane boiling point (101.2 °C).

This reaction presents a series of unusual behaviors which are not readily explained and is therefore an excellent case study to validate our computational method. These behaviors are (i) the need for water-accelerated conditions; (ii) the low impact of stirring rate; and (iii) the counterintuitive salt effect of Na_2_SO_4_.

### 3.1 Comparison of Computational Methods for Water-Accelerated Reactions

A reliable in silico technique for the discovery of water-accelerated reactions, via decreases in activation energy, is an important enabling technology. While DFT studies showing stabilization of transition states through the inclusion of explicit water molecules in nucleophilic substitution, cyclocondensation, and Claisen rearrangement are known,^42−45^ purposeful modeling of water-accelerated reactions is rare. The most recent study was carried out by Jung and Marcus in 2007, using a relatively low-level method and basis set, i.e., B3LYP/6-31+G(d), given the importance of hydrogen-bond-stabilized transition states in these reactions.^31^ Thus, we compared several molecular modeling methods for predicting Δ*G*^‡^ and the water-induced change in activation energy barriers (via inclusion of explicit water molecules) of three known water-accelerated reactions with experimental data.^5^ These methods are PM6-D3H4, B3LYP/6-31+G(d,p), M06-2x/def2-SVP, wB97X-D/def2-TZVP, and wB97X-D/ma-def2-TZVP (Supporting Information, Section S2.5). Attempts at using more accurate DFT and wavefunction methods, e.g., wB97X-V and DLPNO-CCSD(T), were unsuccessful due to the large size and multi-fragment nature of the transition states. The use of very diffuse basis sets (def2-TZVPD and aug-cc-pVTZ) led to basis set near-linear dependencies and failed self-consistent field convergence.^46^ In each case, the activation energies are calculated in toluene and toluene with explicit water molecules. The results indicated that only methods M06-2x/def2-SVP, wB97X-D/def2-TZVP, and wB97X-D/ma-def2-TZVP produced the decreases in Δ*G*^‡^ which follow the trend observed experimentally in these reactions. However, the calculated value of Δ*G*^‡^ for the cycloaddition reaction using wB97X-D/ma-def2-TZVP was too high (35.4 kcal mol^–1^), given that it only takes 10 min to reach completion at 23 °C. In addition, the CPU time required for M06-2x/def2-SVP optimization of transition states is normally an order of magnitude lower compared to that of wB97X-D/ma-def2-TZVP in potential high-throughput in silico screening. Similar observations have also been reported for calculations of intermolecular interactions.^47^ Thus, M06-2x/def2-SVP was taken forward as the method of choice for studying water-accelerated organic reactions.

### 3.2 Computational Studies of Water-Accelerated Henry Reaction

**Figure 3:**
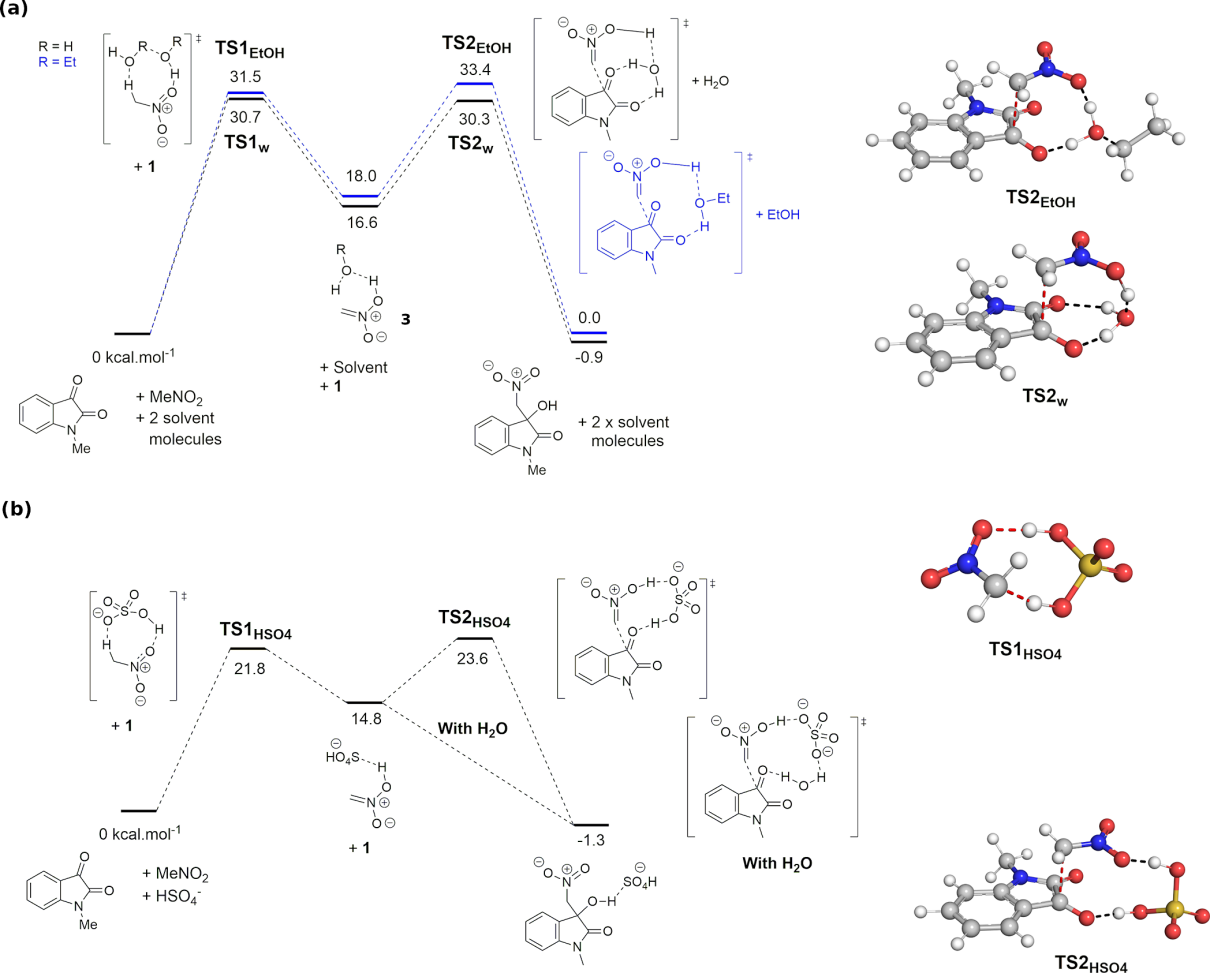
Energy profile of Henry reaction under (a) water-accelerated conditions, or in ethanol, and (b) in the presence of HSO_4_^–^.

The M06-2x/def2-SVP method was applied to the reaction between **1** and nitromethane. Due to the reaction conditions with excess nitromethane, a conductor-like polarizable continuum model (CPCM) solvent model of nitromethane was used with explicit water molecules. For a reaction in an organic solvent, the CPCM solvent model of ethanol with explicit ethanol molecules was used to provide the pre-requisite proton for enolization (Table 1, entries 3 and 5). A two-step mechanism was identified, with **step 1** being the enolization of nitromethane into its nucleophilic form **3** and **step 2** being the nucleophilic attack on **1** (Figure 3a). Under water-accelerated conditions, **step 1** is the RDS with Δ*G*^‡^ = 30.7 kcal mol^–1^ (**TS1**_**w**_). Analysis of the HOMO of **TS2**_**w**_ showed the expected interaction between the HOMO of the enolized nitromethane and the LUMO of ketone **1** (see Supporting Information, Figure S22) The reaction in ethanol resulted in an increase of 2.7 kcal mol^–1^ in Δ*G*^‡^ (**TS2**_**EtOH**_) and a switch of the RDS to **step 2**. Both **TS1**_**EtOH**_ and **TS2**_**EtOH**_ are higher in energy than **TS1**_**w**_ and **TS2**_**w**_, as ethanol is a better hydrogen bond acceptor than the donor compared to water, leading to less stabilization of the proton transfer transition states.^48^ However, the majority of the increase in activation energy comes from **TS2**_**EtOH**_. While **TS2**_**w**_ is stabilized with three H-bonds to the water molecules, only two of those H-bonds are possible with ethanol in **TS2**_**EtOH**_. Close examination of the distances of the forming C–C bond in **TS2**_**EtOH**_ (C···C 2.263 Å) and **TS2**_**w**_ (C···C 2.252 Å) indicated a later transition state under water-accelerated conditions, which correspond to a lower Δ*G*^‡^. These computational results are consistent with our experimental observations, with a significant decrease in activation energy barrier when switching from the ethanol solvent to water-accelerated conditions (Table 1). The identification of **step 1** as the RDS under water-accelerated conditions is also in agreement with the zero-order kinetics of the reaction in [**1**].

Importantly, the calculated mechanism under water-accelerated conditions provides rationalization for the observed strong dependence on temperature and the low dependence on stirring rate. An activation energy barrier of 30.7 kcal mol^–1^ is generally considered high enough to prevent significant conversion at room temperature. Thus, elevated temperature, e.g., 70 or 90 °C, is required. As the reaction is intrinsically limited in rate, increasing mass transfer and organic/aqueous surface area by increasing stirring rate has a limited impact on the reaction rate.

Based on the identification of **step 1** as the RDS under water-accelerated conditions and the observed rate acceleration with phosphate buffer 0.1 M at pH 7, it was hypothesized that Na_2_SO_4_, which is better than NaCl at salting-out nitromethane from the aqueous phase,^36^ may also catalyze **step 1** as a proton transfer catalyst. The active catalytic form HSO_4_^–^ has the pre-requisite proton and is present in very low concentration at pH 7 (p*K*_a_ = 1.92).^49^ Thus, M06-2x/def2-SVP was used to calculate the reaction pathway with HSO_4_^–^ as the proton transfer catalyst in place of water molecules.

**Figure 4:**
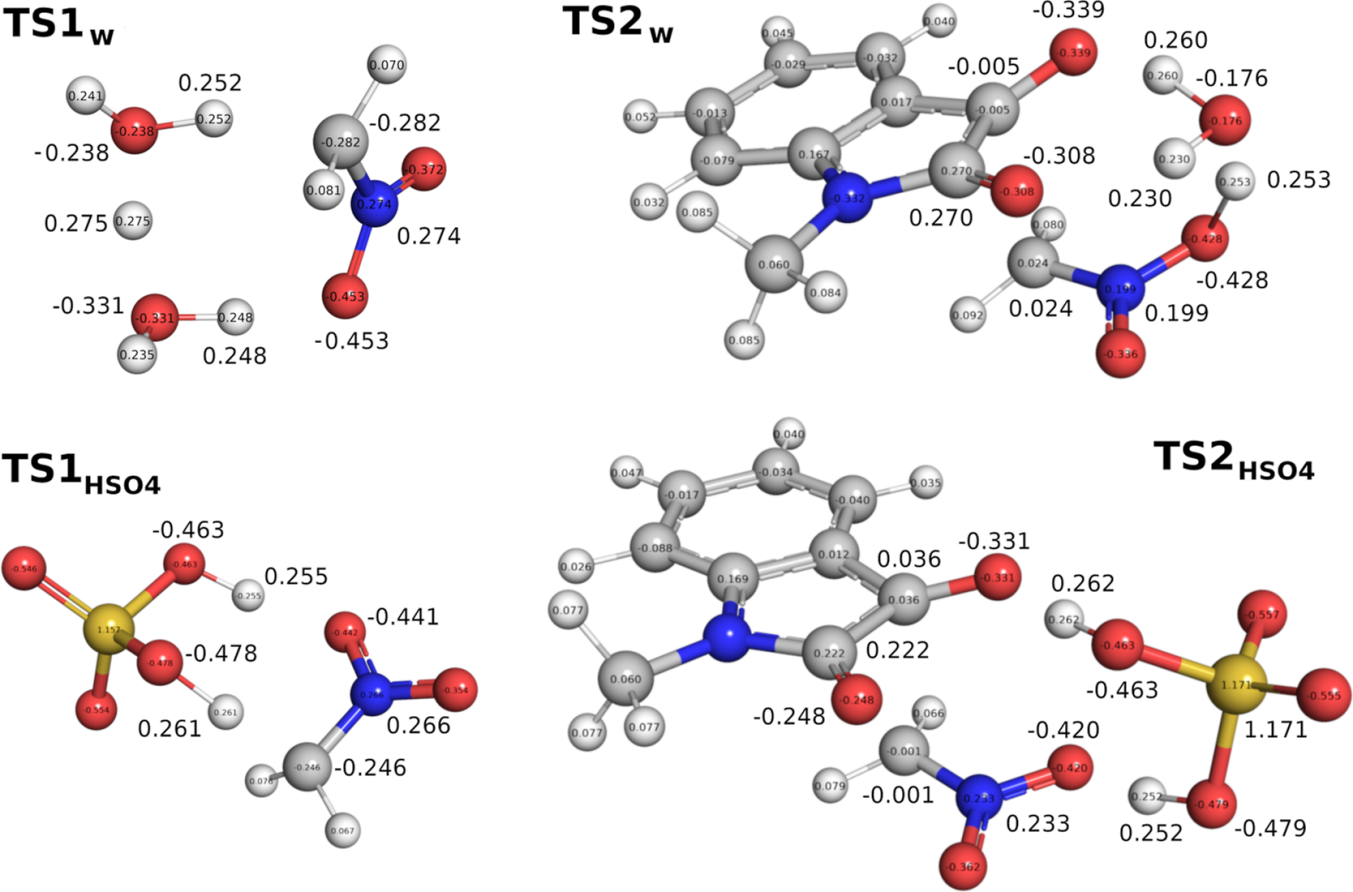
Natural bond orbital (NBO) charge distribution analysis of the transition states **TS1**_**w**_, **TS2**_**w**_, **TS1**_**HSO**_4__, and **TS2**_**HSO**_4__.

The results of this calculation showed large decreases of 8.9 and 6.7 kcal mol^–1^ in the activation energy barrier for **step 1** and **step 2**, respectively (Figure 3b, **TS2**_**HSO**_4__ still has a small second imaginary frequency of 25.6 cm^–1^ after exhaustive optimization). Examination of the natural bond orbital (NBO) charges showed a lesser build-up of charges on the enolized form of nitromethane in **TS1**_**HSO**_4__ (C −0.246 and N 0.266) compared to those in **TS1**_**w**_ (C −0.282 and N 0.274, Figure 4). The same trend was observed for **TS2**_**HSO**_4__ (O_2_N–C −0.001 and C=O −0.248) and **TS2**_**w**_ (O_2_N–C 0.024 and C=O −0.308). These contributed to the lower energies of **TS1**_**HSO**_4__ and **TS2**_**HSO**_4__. Importantly, when an additional molecule of water was included in **step 2** with HSO_4_^–^, no transition state was found. Instead, all attempts at finding the transition state optimized directly to the final product. Thus, it is likely that **step 2** is barrierless under water-accelerated conditions with Na_2_SO_4_ 1 M, leaving **step 1** as the RDS. The very significant decrease in Δ*G*^‡^ of the reaction is partially countered by the low concentration of HSO_4_^–^ (∼10^–5^ M at pH 7) and provides a rationale for the observed rate enhancement of the reaction in Na_2_SO_4_ 1 M. This computational explanation was experimentally verified. A reaction performed in NaHSO_4_ × 10^–5^ M was found to reach 94% conversion in 10 min. Increasing the concentration of Na_2_SO_4_ from 1 to 2 M also led to an increase in yield from 38 to 93% at 10 min of reaction time. This is the first reported example of Na_2_SO_4_ acting as a proton transfer pre-catalyst.

### 3.3 Sustainable Flow Process for Water-Accelerated Henry Reaction

To achieve sustainable processes with water-accelerated reaction, recycling of the aqueous phase is essential. Consequently, a continuous process with in-line phase separation and aqueous phase recycling was envisioned. Theoretical calculation suggested a possible decrease in PMI-reaction (process mass intensity of reaction) metrics from 33 in the literature batch protocol (method A, Table 2) to 8 in flow (with 25 equiv of nitromethane),^35^ if the aqueous phase can be recycled at least 15 times (see Supporting Information, Section S1.11). Full-process PMI including work-up for flow processes can be highly dependent on scale, and prior studies have sometimes excluded work-up from its calculation.^50,51^ Thus, PMI-reaction, which excludes evaporation of nitromethane to isolate the product, will be used in this study for consistency.^52^ To circumvent traditional extraction, which suffers from slow phase separation and requires an additional amount of organic solvent, a membrane-enabled continuous phase separation using a Zaiput device was selected.^53^

**Table 2:** Comparison of Input Materials in Batch and Batch/Flow Protocols^a^

method	1 (g)	MeNO_2_ (mL)	aqueous phase (mL)^b^	reaction time (min)	PMI/PMI-reaction
A	0.081	0.11	3^c^	30	33
B	0.225	2.1	3^c^^,^^d^	180^c^/10^d^	17^c^^,^^e^/16^d^^,^^e^
C	0.051	0.42	0.42^d^	10	14
D	18.7	155	12^d^	10	10
E	16.6	50	13^d^	20	4

a Method A: literature batch protocol at room temperature;^35^ method B: DoE-optimized batch protocol with recycling of the aqueous phase; method C: batch protocol using a 1:1 phase ratio at 40 °C, no recycling; method D: flow protocol using 25 equiv of nitromethane at 40 °C with continuous aqueous phase recycling (13 cycles); and method E: flow protocol using 9 equiv of nitromethane at 40 °C with continuous aqueous phase recycling (4 cycles).

b Including top-up water/buffer solution.

c Using water.

d Using 0.1 M phosphate buffer pH 7.

e Calculated for batch protocols with recycling of water (at 70 °C) or 0.1 M phosphate buffer (25 °C), 5 cycles.

**Figure 5:**
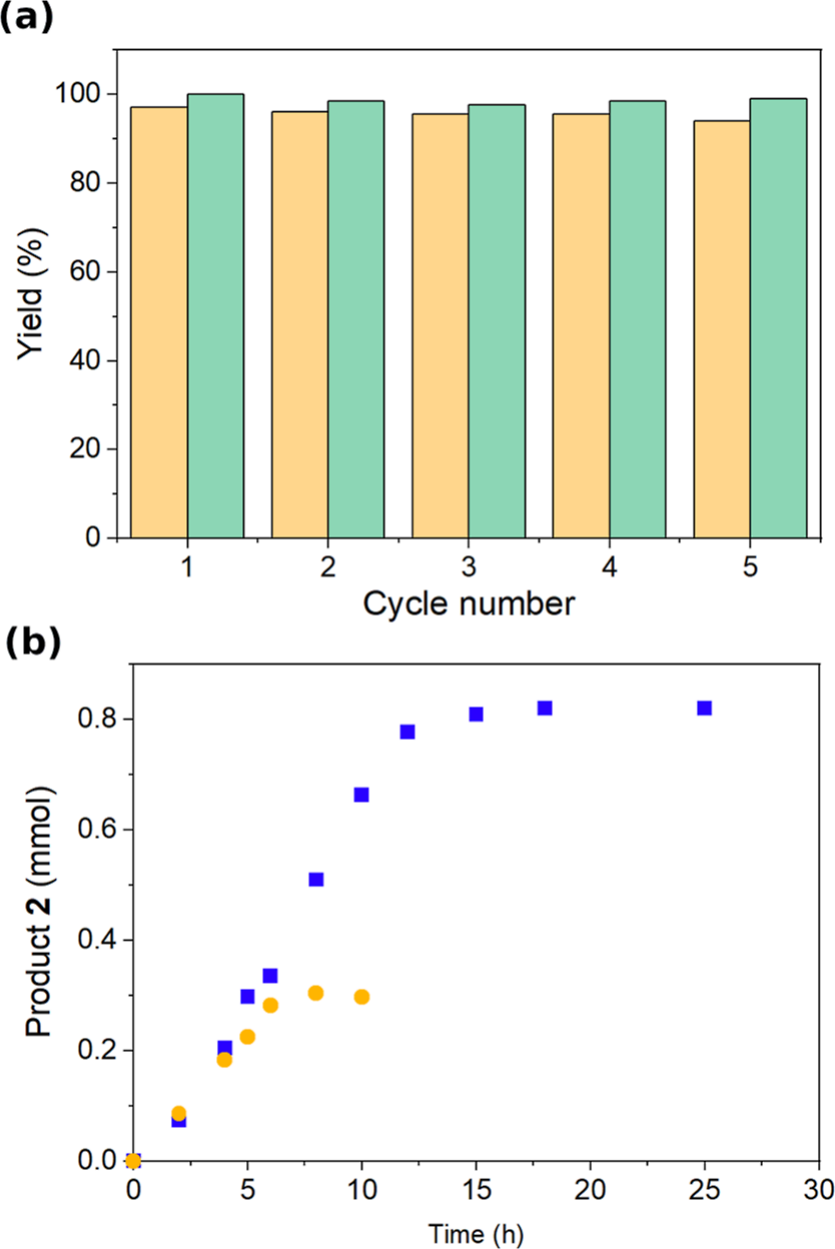
(a) Reaction yields with recycling of the aqueous phase in batch, orange bar depicting yield with water as the aqueous phase and green bar depicting yield with 0.1 M phosphate buffer pH 7 as the aqueous phase. Reaction conditions: 51 mg of **1**, 0.42 mL of nitromethane, 3 mL of aqueous phase recycled up to 5 times, 850 rpm, 70 °C (water), 25 °C (phosphate buffer), 3 h (water), 10 min (phosphate buffer). (b) Kinetic profile of Henry reaction in batch at a 1:1 phase ratio using 25 equiv (●) or 9 equiv (■) of nitromethane. Reaction conditions: 0.42 mL solution of **1** in nitromethane with 5 mol % trimethoxybenzene as an internal standard [(●) 0.121 g **1**/1 mL; (■) 0.331 g **1**/1 mL], 0.42 mL of 0.1 M phosphate buffer, 40 °C, 850 rpm, 0–25 min.

Initial water recycling studies in batch, based on conditions developed via DoE (method B, Table 2), showed no changes to reaction yield in up to 5 cycles (Figure 5a). The 3 h reaction time in water at 70 °C was considered too long for flow. Thus, a 0.1 M phosphate buffer was used instead of water, and the temperature was kept at 25 °C to give a reaction time of 10 min for up to 100% yield (method B, Table 2). Again, there was no change in reaction yield after recycling the buffer solution 5 times (Figure 5a). However, phase separation post-reaction was slow, taking up to 16 h to fully separate when water was used. When a 1:1 phase ratio was employed (method C), the temperature was increased to 40 °C to maintain a reaction time of 10 min, giving the same yield. A small amount (∼3%) of a new side product, **4** (Figure 7), was detected at this stage, due to contamination of isatin in a new commercial batch of starting material **1**. Prolonged exposure of either **1** or **2** to the reaction conditions did not result in any formation of **4**, ruling out a demethylation reaction.

**Figure 6:**
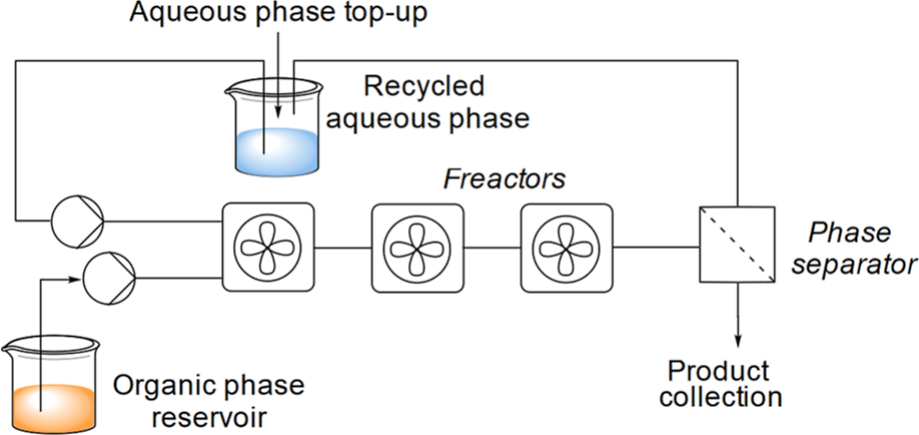
Flow process diagram including the continuous recycling of the aqueous phase.

Flow experiments were performed using a cascade of three mini-CSTRs named fRreactors (Figure 6).^54^ These fReactors have proven effective at carrying out multiphase reactions with longer reaction times in continuous flow, where reproducible mixing can be important in process control. A phase ratio of 1:1 (v/v) was used to improve the phase separation using the Zaiput membrane separator. These changes also improved the productivity of the process and provided a satisfactory mass balance. When higher aqueous–organic phase ratios were used, a lower yield of product was observed, which was attributed to partial solubility of **1**, nitromethane, and product **2** in the aqueous phase.

Initial flow experiments employed a residence time of 10 min using 3× fReactors with a total reactor volume of 4.8 mL, at a combined 0.48 mL min^–1^ flow rate. Multiphase mixing is very poor inside normal 1/8 in. PTFE tubing, and thus, the reaction was considered complete after the CSTRs. Various PTFE hydrophobic and hydrophilic membranes from Zaiput were tested for phase separation. For a lower aqueous to organic phase ratio (1:1), the hydrophobic membrane was required, and the pore size was selected based on the type of pump used. The best results were obtained with OB-900-S10 (for syringe pumps) and OB-2000-S10 (for diaphragm/rotary piston pumps). At higher aqueous to organic phase ratios (up to 7:1), the hydrophilic membrane IL-900-S10 was used with good separation and without breakthrough. The separated aqueous phase, which contains partially dissolved nitromethane, **1**, and **2**, was recycled through a reservoir, using a recirculation pump (Figure 6).

**Figure 7:**
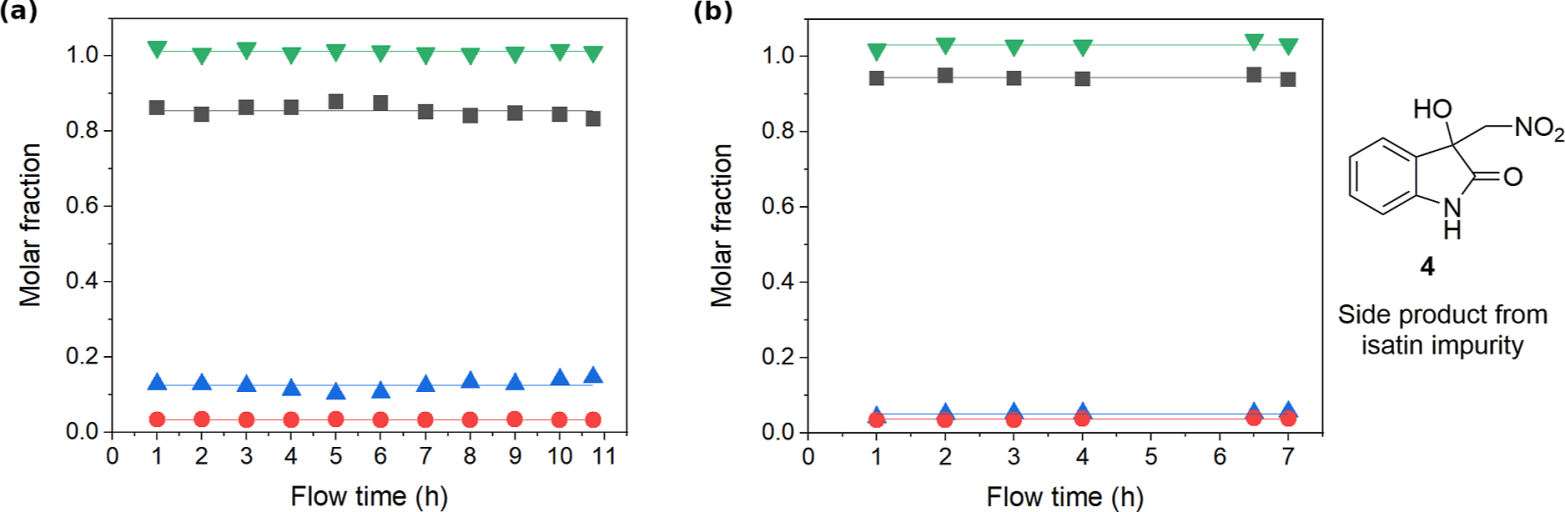
Yields and mass balance of flow processes with continuous aqueous phase recycling using (a) 0.121 g of **1** per 1 mL CH_3_NO_2_ and (b) 0.3331 g of **1** per 1 mL CH_3_NO_2_. (▲) Molar fraction of **1**; (■) molar fraction of product **2**; (●) molar fraction of side product **3**; and (▼) total mass balance.

The output of the flow process was analyzed by ^1^H NMR, showing a stable composition of **1**, **2**, and **4**, with an average yield of 85.5 ± 1.4% over 11 h, which corresponds to 13 cycles of aqueous phase recycling (12 mL at 0.24 mL min^–1^ flow rate) and a very high space-time-yield (STY) of 0.47 kg L^–1^ h^–1^ (method D, Table 2 and Figure 7a). However, partial solubility of water in nitromethane led to a gradual loss of the aqueous phase (initially 9 mL) over time. Thus, the aqueous phase was topped-up at 4.5 h (2 mL) and 8 h (1 mL). The PMI-reaction value for the process over 11 h was calculated as 10. This is slightly above the optimal theoretical value 8 due to our experiment being stopped at 13 cycles and the need for top-up of the aqueous phase.

To further improve the sustainability of the process, the amount of **1** was increased to reduce the nitromethane/**1** ratio from 25 to 9, while maintaining the same aqueous–organic phase ratio of 1:1. The 1:9 molar ratio between **1** and nitromethane is the solubility limit of **1** in nitromethane at room temperature. Furthermore, reducing the amount of hazardous nitromethane is an important consideration for the greenness of the process. This change led to an increased reaction time of 20 min in both batch and the fReactors (Figure 5b), due to the zero-order kinetics of the reaction. The aqueous phase was topped-up at 3.5 h (0.5 mL) and 5.5 h (0.5 mL). The flow process worked well and gave an average yield of 94.4 ± 0.5% (no further purification other than solvent evaporation was required) and an STY of 0.64 kg L^–1^ h^–1^ over 7 h (method E, Table 2 and Figure 7b). This consists of 4 cycles (13 mL at 0.12 mL min^–1^ flow rate), giving an impressive PMI-reaction value of 4. As a comparison, typical organic syntheses in industry have an average PMI value of 10–20 per step, with a significant contribution from organic solvents as reaction and purification media.^55^ These can be readily minimized in processes based on water-accelerated reactions.

## 4 Conclusions

We report in this manuscript a comprehensive computational and experimental framework for studying, rationalizing, and applying water-accelerated reactions in sustainable processes. These are complex phenomena which are influenced by many factors, e.g., phase behaviors, mass-transfer/mixing, solubility, and water-stabilized transition states, and can display different responses to external stimuli, depending on the complex physical and chemical aspects of the system. For the water-accelerated Henry reaction between *N*-methylisatin **1** and nitromethane, a combination of phase behaviors, solubility, and modern DFT modeling with explicit water molecules provided an excellent rationalization for (i) the rate acceleration under water-accelerated conditions; (ii) the low impact of stirring rate; (iii) and the unexpected rate acceleration effect of Na_2_SO_4_ as the source of a proton transfer catalyst. Importantly, we demonstrated the sustainability of continuous processes employing water-accelerated reactions when optimized for recycling of the aqueous phase, enabled by continuous phase separation. No degradation of yield and purity profile was observed when the aqueous phase was recycled up to 13 times. The use of multiphase flow reactors with excellent mixing led to exceptional sustainability metrics (STY = 0.64 kg L^–1^ h^–1^ and PMI-reaction = 4). The experimental and theoretical framework reported here will underpin the future discovery and development of this important class of reactions into sustainable manufacturing processes.

## Supporting Information Available

Experimental procedures and calculations of green metrics and DFT computational details (PDF)

## Abbreviations

PMI: process mass intensity
RDS: rate-determining step
DFT: density functional theory
CSTR: continuous stirred tank reactor
