# Supplementary material for: Rationalizing and Adapting Water-Accelerated Reactions for Sustainable Flow Organic Processes
Source: ACS Sustain Chem Eng. 2023 May 30;11(23):8675–84. doi: 10.1021/acssuschemeng.3c02164 (PMC10265699; doi:10.1021/acssuschemeng.3c02164)
Supplement: Supplementary file 1 — sc3c02164_si_001.pdf [file sc3c02164_si_001.pdf]

Rationalizing and Adapting Water-Accelerated Reactions for Holistically Sustainable Flow Organic Processes

*Katarzyna A. Maltby, Krishna Sharma, Marc A. S. Short, Sannia Farooque, Rosalie Hamill, A. John Blacker, Nikil Kapur, Charlotte E. Willans, Bao N. Nguyen**

Electronic Supporting Information

1 Experimental procedures S3
1.1 General information S3
1.2 Standard protocol for Henry reaction S4
1.3 Reactions in D_2O S10
1.4 Reactions in the presence of different salts S11
1.5 Effect of process parameters S12
1.6 Recycling of aqueous phase in batch S13
1.7 Flow experiments and NMR analysis with trimethoxybenzene as an internal standard S17
1.8 Continous aqeuous phase recycling S20
1.9 Measurements of solubility of $MeNO_2$ S24
1.10 PMI-reaction calculations S25
1.11 Space-time-yield calculations S28
1.12 PMI-reaction and STY calculations for literature examples S30
2 Comparing molecular modelling techniques for known water-accelerated reactions S34
2.1 General procedure S34
2.2 Cycloaddition reaction S34
2.3 Diels-Alder reaction S50
2.4 Claisen Rearrangement S67
2.5 Summary of different molecular modelling techniques S79
3 DFT calculations of Henry reaction S82
3.1 Calculations in nitromethane S82
3.2 Calculation in ethanol S86
3.3 Calculation in nitromethane at 90 ºC S89
3.4 Calculation in nitromethane with Na_2SO_4 .... S92
4 References S95

1 Experimental procedures

1.1 General information

All solvents and reagents were purchased from commercial sources. The solvents were HPLC standard and purchased from Sigma Aldrich. Chemicals were purchased from Sigma-Aldrich (Dorset, UK), Alfa Aesar (Heysham, UK) and Flurochem Ltd. (Glossop, UK) unless stated otherwise. Nuclear magnetic resonance (NMR) spectra were recorded for ^{1}H at 400 and 500 MHz and ^{13}C at 100 and 125 MHz on a Bruker DPX400 or DRX500 spectrometer. Bruker DRX 500 spectrometer was equipped with a multinuclear inverse probe for one-dimensional ^{1}H and two-dimensional heteronuclear single quantum coherence (^{1}H−^{13}C HSQC), heteronuclear multiple bond correlation (^{1}H−^{13}C HMBC), and double quantum filtered correlation (^{1}H−^{1}H COSY). Chemical shifts (δ) are quoted in ppm downfield of tetramethylsilane or residual solvent peaks (7.26 and 77.16 ppm for $CDCl_3$ in ^{1}H and ^{13}C, respectively). The coupling constants (J) are quoted in Hz (multiplicities: s singlet, bs broad singlet, d doublet, t triplet, q quartet and apparent multiplicities are described as m).

The analytical TLC chromatography was carried out using alumina-backed plates coated with silica gel 60 with a fluorescence indicator (20 × 20 cm, Merck) and nanosilica gel C18-100 with a fluorescence indicator (10 × 10 cm; no. 811062). HPLC system (Agilent 1290 infinity series) was equipped with diode-array detector (DAD), binary pump system connected with online degasser and Zorbax Eclipse XDB C18, 150 × 4.6 mm, 5 µm. The flow rate and the injection volume were 1 mL/min and 10 µL respectively. The chromatograms were recorded by scanning the absorption at 190−600 nm. High resolution mass spectra (HRMS) were recorded on a Dionex Ultimate 3000 spectrometer using electron spray ionization (ESI). All masses quoted are correct to four decimal places. Infrared (IR) spectra were recorded using a PerkinElmer Spectrum One FT-IR spectrophotometer or Bruker Alpha Platinum AR FTIR. Vibrational frequencies are reported in wavenumbers (cm^{-1}).

Liquid chromatography was carried out on Agilent 1200 LC with a Bruker HCT Ultra Ion Trap for MS detection and a photodiode array detector (PAD) for UV/Vis measurements. The electron spray ionization (ESI) parameters for the positive ionization (PI) mode were as follows: spray voltage: 4000 V; dry gas flow rate: 10 dm^3 min^{-1}; dry gas temperature: 365 °C; capillary: 60 nA; nebulizing pressure: 65 psi; nebulizing gas: N_2. The ESI (electrospray ionization) parameters in the NI (negative ion) mode were as follows: spray voltage 4000 V (applied to the spray tip needle), dry gas 10 dm^3 min^{-1}, dry temperature 365 °C, capillary 60 nA, nebulizer 65 psi, nebulizing gas N_2. Solvents were removed under reduced pressure using a Buchi rotary evaporator at 20 mbar, followed by further drying under high vacuum at 0.5 mmHg.

1.2 Standard protocol for Henry reaction

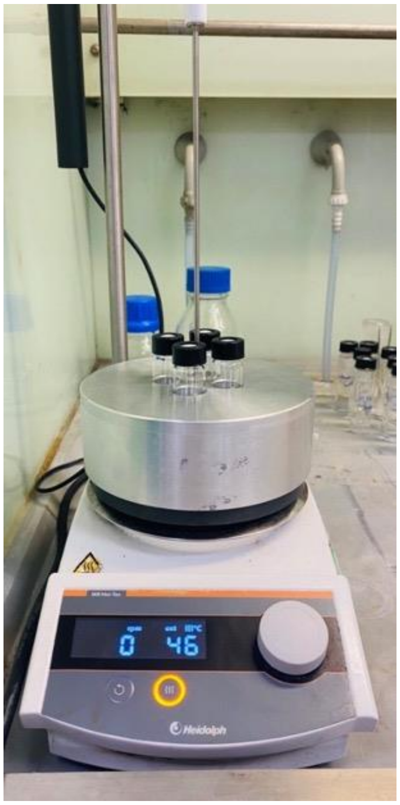

Figure S1. Experimental setup for standard batch reactions

The reactions were heated and stirred (1 cm × 1 cm cross-bar stirrer) on a custom-made heating block with 1 inch off-set stirring (Figure S1). Reactions were carried out in 4-dram glass vials (2.1 × 7 cm, 14 mL) sealed with a lid (PTFE septum) unless stated otherwise.

Methylisatin **1** (0.0806 g, 0.5 mmol) was added to a 4-dram vial (2.1 × 7 cm) equipped with a cross-bar stirrer (1 cm × 1 cm) followed by nitromethane (812 µL, 15 mmol). After 5 minutes deionized water (3 mL) was added and the sample tube was sealed with a lid and heated to 70 °C at 700 rpm. After three hours the reaction mixture was extracted with ethyl acetate (10 mL) to afford the product **2** as pale-yellow oil (0.1077 g, 97%). IR (neat): ν_{max} 3366, 2958,2930, 2860, 1727, 1686, 1609, 1547, 1469, 1318, 1095, 759 cm^{-1}.

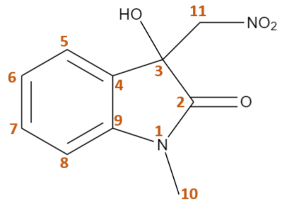

^{1}H NMR (400 MHz, $CDCl_3$): δ 7.42 – 7.32 (m, 2H, H-7 and H-8, 7.13 (t, *J* = 7.6, 1H, H-6), 6.89 (d, *J* = 7.6 Hz, 1H, H-5), 4.94 – 4.77 (m, 2H, H-11), 4.58 (s, 1H, OH), δ 3.21 (s, 3H, H-10). ^{13}C NMR (100 MHz, $CDCl_3$,): δ 174.8 (CO, C-2), 143.9 (C_q, C-9), 131.4 (C-8), 125.9 (C_q, C-4), 124.5 (C-7), 123.9 (C-6), 109.4 (C-5), 78.5 (CH_2, C-11), 73.5 (Cq, C-3) and 26.8 (CH_3, C-10). ESI MS (m/z): 223 $(M+H)^+$. HRMS(ESI) calcd for $C_{10}H_{10}N_2O_4Na$ $[M+Na]^+$ 245.0551, Found 245.0550.

Lit. values: ^{1}H NMR (300 MHz, $CDCl_3$): δ 3.22 (3H, s, CH_3), 4.58 (1H, s, OH), 4.88 (2H, m, CH_2), 6.89 (1H, d, *J* = 8.1 Hz, Ar), 7.13 (1H, t, *J* = 8.1 Hz, Ar), 7.40 (1H, t, *J* = 8.1 Hz, Ar), 7.42 (1H, d, *J* = 8.1 Hz, Ar).[1] ^{13}C NMR (100 MHz, $CDCl_3$, TMS): δ 25.8, 72.2, 95.4, 108.2, 122.2, 123.8, 126.6, 130.0, 143.6, 173.9.[2]

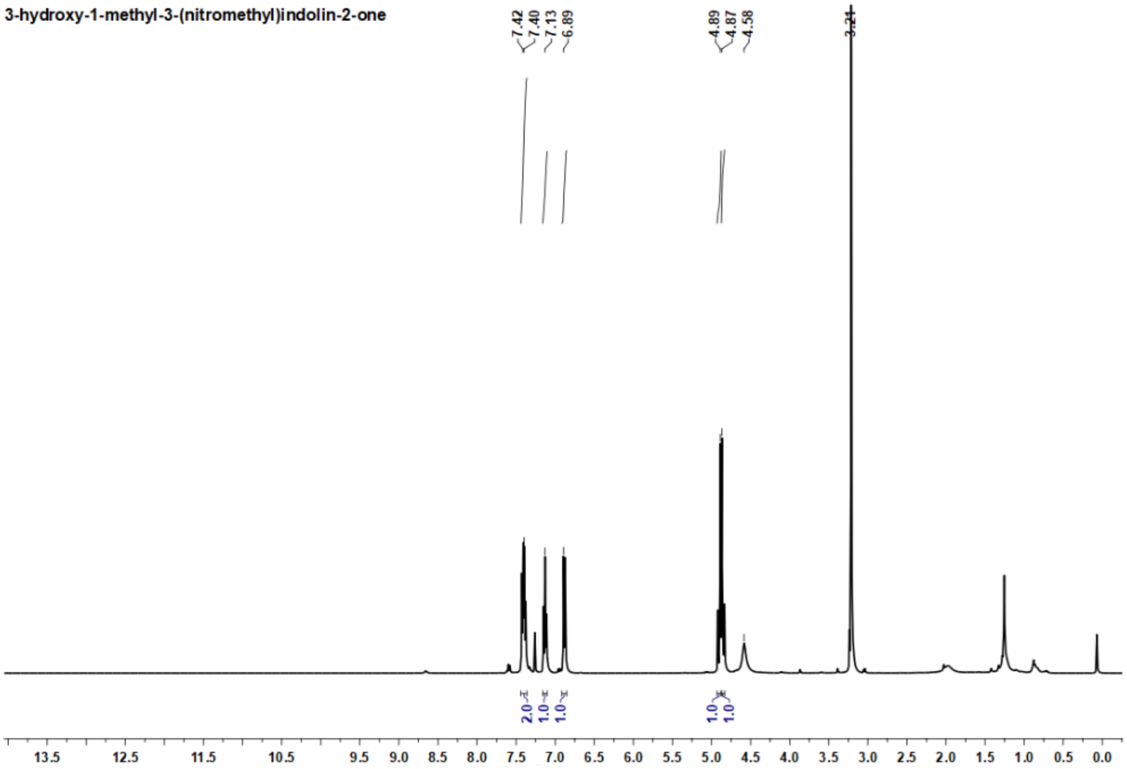

Figure S2. ^{1}H NMR spectrum for the product **2**. Grease: 0.80 and 1.30 ppm, $CHCl_3$: 7.26 ppm, water: 1.50 ppm.

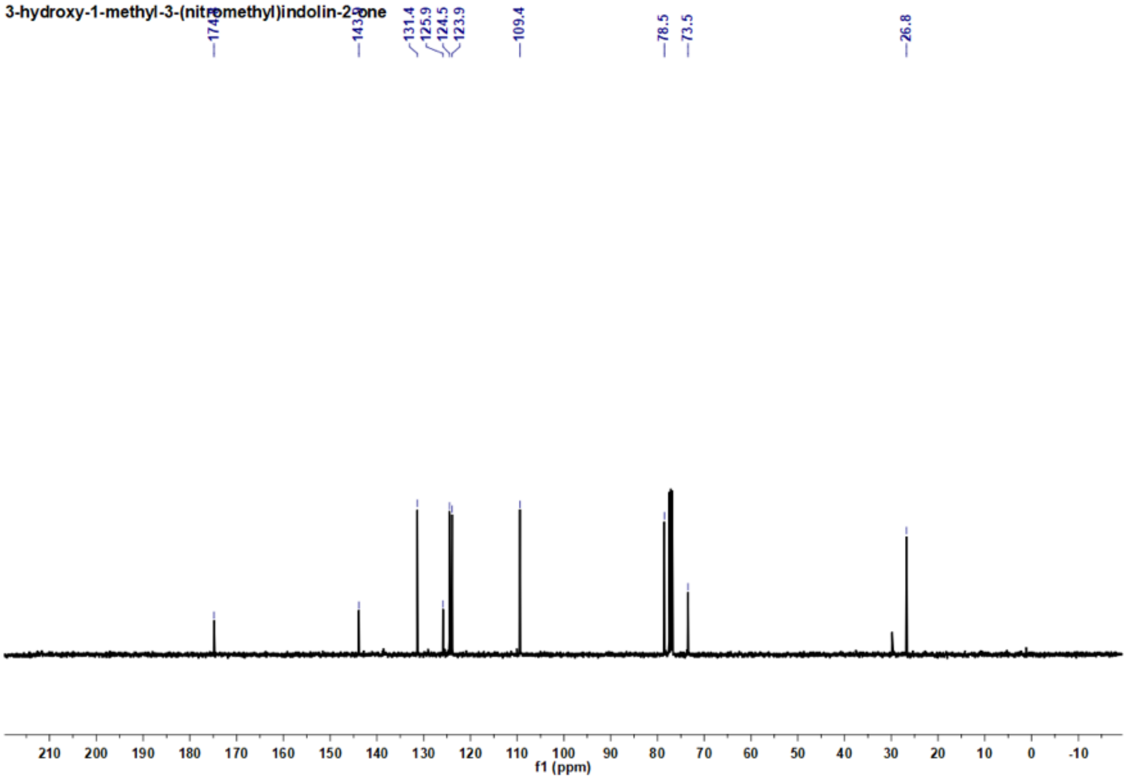

Figure S3. ^{13}C NMR spectrum for the product **2**. Grease: 28.5 ppm, $CDCl_3$: 77.16 ppm.

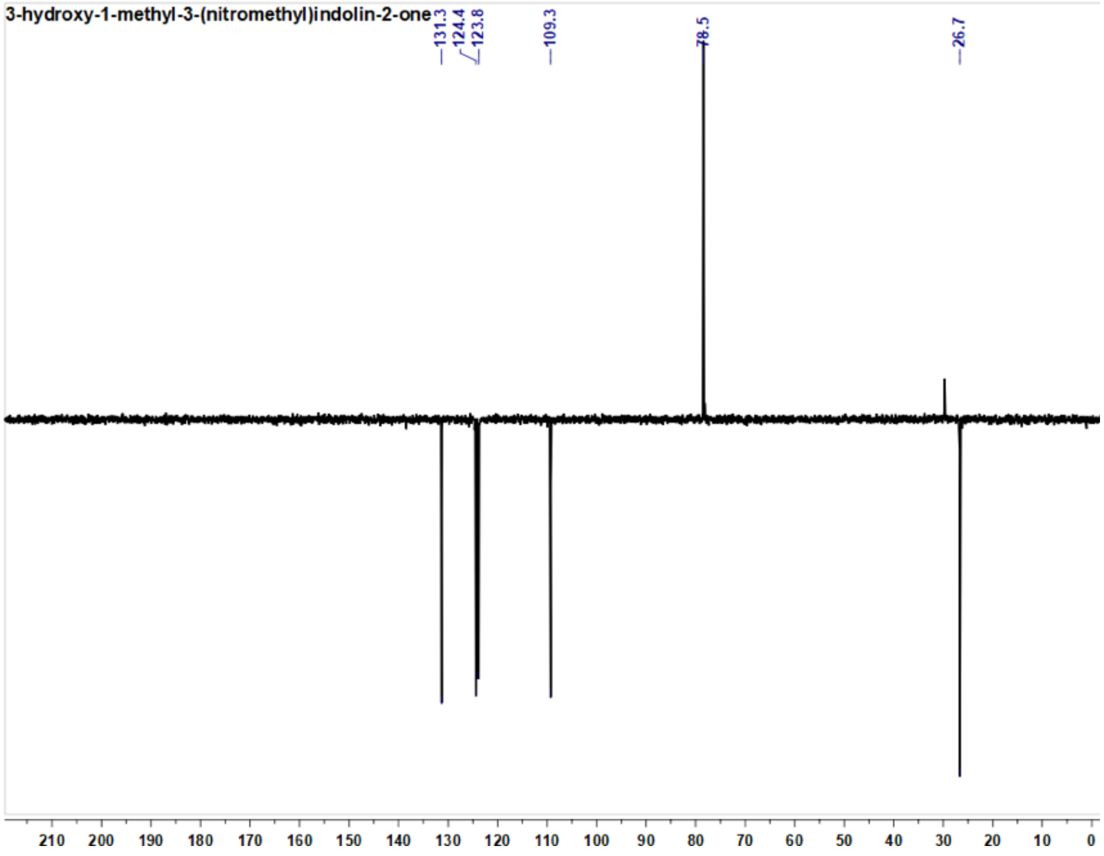

Figure S4. DEPT135 NMR spectrum for the product **2**. Grease: 28.5 ppm.

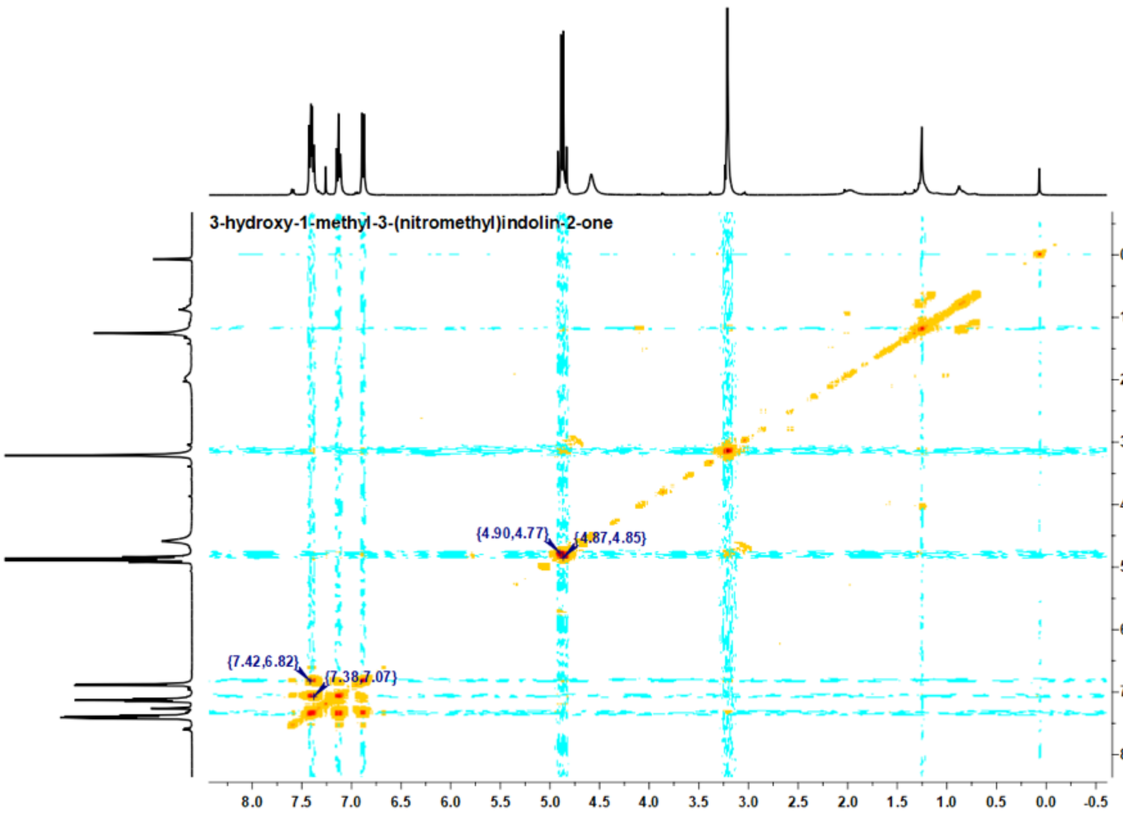

Figure S5. COSY ^{1}H-^{1}H NMR spectrum for the product **2**

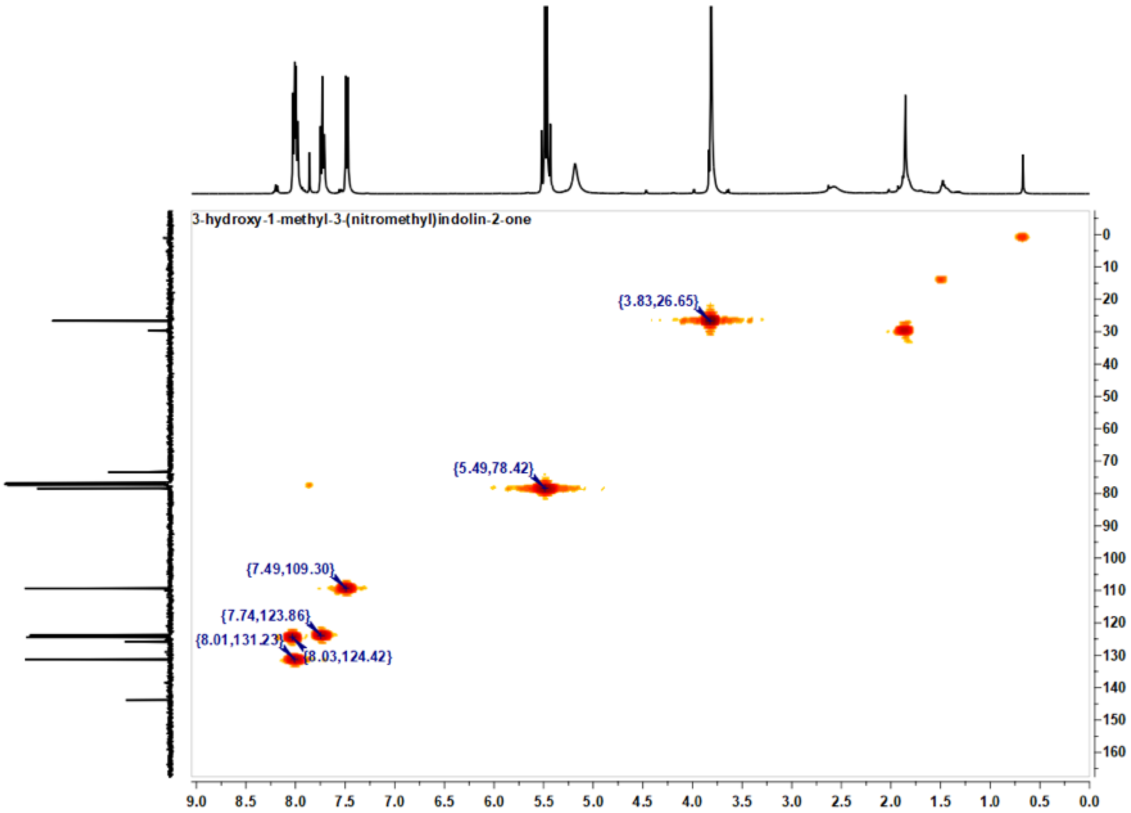

Figure S6. HSQC ^{1}H-^{13}C NMR spectrum for the product **2**

Some batches of methylisatin are contaminated with isatin (non methylated compound), thus sometimes a co-product **4** of reaction is observed. The co-product **4** was isolated from reaction mixture by evaporating nitromethane and using preparative LC to separate the co-product (Agilent 1260 Preparative LC with multiwave detector, column: Phenomenex Kinetex 5 um EVO C18 column (250 x 21.2 mm)). The sample was prepared by redissolving solid crude product in 1:1 mixture of acetonitrile and water (188 mg in 2 mL), mobile phase: acetonitrile and water with 0.1 % TFA (trifluoroacetic acid); method details: injection volume 50 µL, gradient: 5-35 % acetonitrile over 25 min, 95 % acetonitrile in 1 min, hold 1 min at 95 %, back to 5 % acetonitrile, overall method time 30 min. See Figure S7 for ^{1}H NMR spectrum of the isolated co-product and relevant literature data.

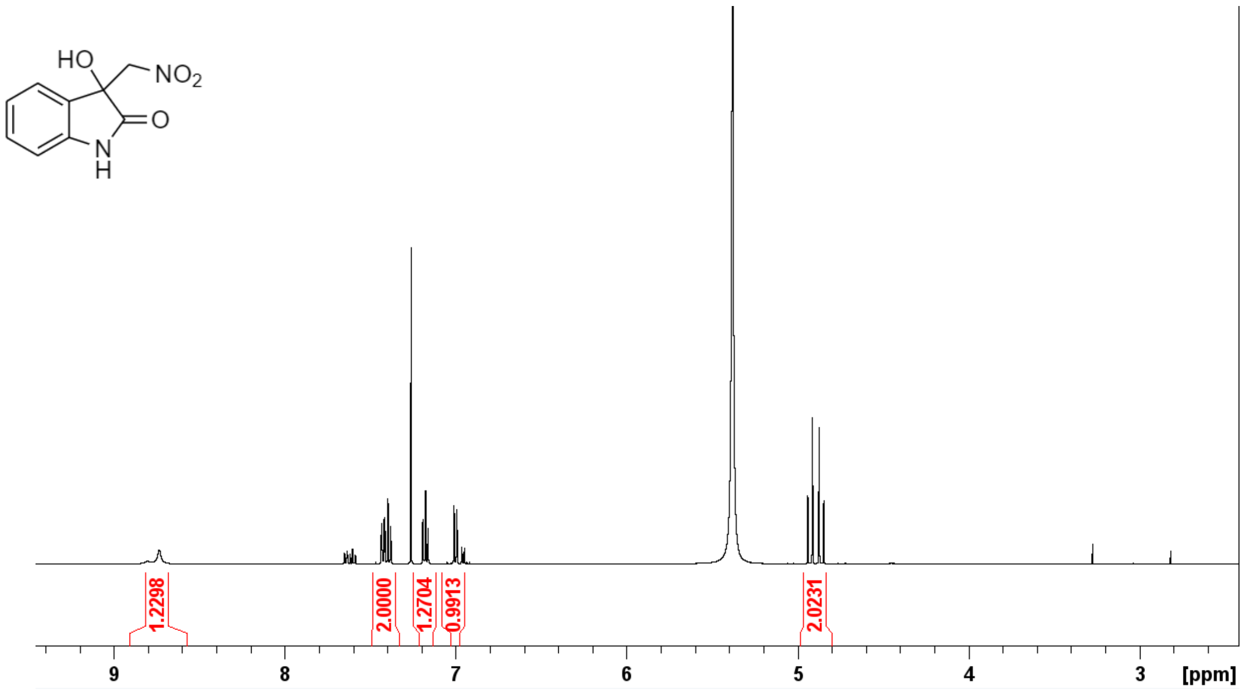

Figure S7. ^{1}H NMR spectrum of isolated co-product **4**, the sample is contaminated with about 22 % isatin (7.62 ppm, 6.96 ppm)

^{1}H NMR (500 MHz, $CDCl_3$): δ 4.80-4.99 (m, 2H, CH_2), 7.00 (d, J = 7.7 Hz, 1H), 7.18 (t, 7.6 Hz, 1H), 7.36 – 7.46 (m, 2H), 8.74 (br s, 1H, NH). ^{1}H NMR (500 MHz, d_6-DMSO): δ 4.94-5.06 (m, 2H, CH_2), 6.71 (s, 1H, OH), 6.85 (d, J = 7.6 Hz, 1H), 6.99 (t, 7.6 Hz, 1H), 7.27 (t, 7.6 Hz, 1H), 7.40 (d, 7.45 Hz, 1H), 10.55 (s, 1H, NH). ^{13}C NMR (125 MHz, d_6-DMSO): δ 72.8, 78.5, 110.0, 121.8, 124.7, 127.9, 130.2, 142.6, 175.9. HRMS(ESI) calcd for $C_9H_8N_2O_4Na$ $[M+Na]^+$ 231.0376, Found 231.0364.

Literature values:[2] ^{1}H NMR (500 MHz, $CDCl_3$+d_6-DMSO): δ 4.81 (d, J = 12.6 Hz, 1H), 4.88 (d, J = 12.6 Hz, 1H), 6.5 (s, 1H, OH), 6.85 (d, J = 7.3 Hz, 1H), 6.95 (t, J = 7.3 Hz, 1H), 7.21 (t, J = 7.3 Hz, 1H), 7.31 (d, J = 7.3 Hz, 1H), 10.3 (s, 1H, NH). ^{13}C NMR (100 MHz, d_6-DMSO): δ 78.2, 95.4, 109.8, 121.5, 124.3, 127.6, 129.8, 142.4, 175.6.

1.3 Reactions in D_2O

The rate of reaction was studied water-accelerated as well as on-D_2O. Six reactions were done water-accelerated and on-D_2O separately, worked up and analyze by ^{1}H NMR for the kinetic data. Methylisatin **1** (0.0806 g, 0.5 mmol) was added to six 4-dram vials (2.1 × 7 cm) separately equipped with cross-bar stirrers (1 × 1 cm) followed by nitromethane (812 µL, 15 mmol). After 5 minutes deionized water (3 mL) was added and the sample tube was sealed with a lid and heated to 70 °C at 700 rpm. The reactions are stopped at different reaction time. After 15, 45,

75, 105, 135 and 165 minutes the reaction mixtures were extracted with ethylacetate to afford the product as pale-yellow oil.

Table S1. Kinetic data of reactions in water and in D_2O

Time (min)	^{1}H NMR conversion (%)	
	Using water	Using D_2O
15	6	4
45	26	10
75	45	16
105	57	25
135	74	32
165	96	37

1.4 Reactions in the presence of different salts

The reactions were performed in deionized water, 1M NaCl, 1M LiCl, 1M Na_2SO_4 and 0.1M phosphate buffer at pH 7. Reactions were done separately, worked up and analyzed by ^{1}H NMR for the kinetic data. Methylisatin **1** (0.0806 g, 0.5 mmol) was added to the 4-dram vials (2.1 × 7 cm) separately equipped with cross-bar stirrers (1 × 1 cm) followed by nitromethane (812 µL, 15 mmol). After 5 minutes the relevant aqueous additive (3 mL) was added and the sample tube was sealed with a lid and heated to 70 °C at 700 rpm. The facile reaction on phosphate buffer was done at room temperature to allow kinetic analysis. After specific time the reaction mixtures were extracted with ethylacetate, dried ($MgSO_4$) and solvent was evaporated to afford the product as pale-yellow oil.

1M NaCl: Sodium chloride (5.85 g, 0.10 mol) was added to deionized water (80 mL) and stirred until completely dissolved. The solution was transferred to a volumetric flask and made up to 100 mL.

1M LiCl: Lithium chloride (4.239 g, 0.10 mol) was added to deionized water (80 mL) and stirred until completely dissolved. The solution was transferred to a volumetric flask and made up to 100 mL.

1M Na_2SO_4: Anhydrous sodium sulfate (14.20 g, 0.10 mol) was added to deionized water (80 mL) and stirred until completely dissolved. The solution was transferred to a volumetric flask and made up to 100 mL.

Phosphate Buffer Solution 0.1 M: $Na_2HPO_4 \cdot 2H_2O$ (1.342 g, 0.0075 mol) and $NaH_2PO_4 \cdot 2H_2O$ (0.3838 g, 0.0025 M) were added to deionized water (80 mL) and stirred until completely dissolved. The solution was transferred to a volumetric flask and made up to 100 mL. The pH was adjusted to 7 using HCl.

1M TBACl: Tetrabutylammonium chloride (5.558 g, 0.020 mol) was added to deionized water (15 mL) and stirred until completely dissolved. The solution was transferred to a volumetric flask and made up to 20 mL.

Table S2. Kinetic data for the effect of additives on the rate of reaction

Time (min)	^{1}H NMR conversion (%)					
	H_2O (1 M)	NaCl (1 M)	LiCl (1 M)	TBACl (1 M)	Na_2SO_4 (1 M)	Phosphate buffer (0.1 M)
1	-	-	-	-	-	24
2	-	-	-	-	-	37
3	-	-	-	-	-	48
4	-	-	-	-	-	59
5	-	-	-	-	15	68
6	-	-	-	-	-	78
10	-	-	-	-	38	-
15	6	5	5	5	56	-
20	-	-	-	-	76	-
25	-	-	-	-	83	-
30	-	-	-	-	90	-
45	26	16	12	12	-	-
75	45	24	21	25	-	-
105	57	34	30	31	-	-
135	74	40	39	32	-	-

1.5 Effect of process parameters

A Design of Experiment (DoE) was used to evaluate the effect of temperature, rate of stirring, ratio of nitromethane to methylisatin and the concentration of methylisatin in reaction mixture; optimum reaction conditions were found using this method and the rate of reaction of optimum method was evaluated and compared with standard method. The reactions were done in the order proposed by DoE method; methylisatin was added to a 4-dram glass vial (2.1 × 7 cm) equipped with a cross-bar stirrer (1×1) followed by nitromethane). After 5 minutes deionized water (3 mL) was added and the sample tube was sealed with a lid and heated and stirred at the conditions given in the table below. After 60 minutes the reaction mixtures were extracted with ethylacetate to afford the product as pale-yellow oil and analyzed by ^{1}H NMR spectroscopy. Reaction rate was calculated based on a zero-order reaction in [**1**].

Table S3. Design of Experiment results of Henry reaction.

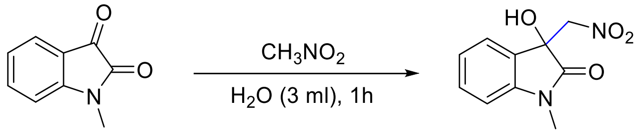

1 **2**

No.	Run order	**1** (mg)	**1** (M)[a]	$MeNO_2$ (µL)	$MeNO_2$/**1**	Phase ratio (aq:org)	T (°C)	Stirring (rpm)	^{1}H NMR Yield (%)	Yield (mmol **2**)	Rate (mM/min)[a]

N3	1	51	0.086	685	40	4.4	50	300	7	0.022	0.100
N9	2	150	0.186	2000	40	1.5	50	1400	1	0.009	0.031
N5	3	51	0.086	685	40	4.4	90	300	93	0.294	1.332
N10	4	51	0.100	171	10	17.5	90	1400	78	0.247	1.299
N11	5	150	0.266	504	10	6	90	1400	63	0.587	2.792
N13	6	51	0.092	428	25	7	70	850	55	0.174	0.847
N8	7	51	0.086	685	40	4.4	50	1400	8	0.025	0.115
N16	8	101	0.144	1350	40	2.2	70	850	18	0.113	0.433
N6	9	150	0.186	2000	40	1.5	90	300	42	0.391	1.304
N17	10	101	0.163	844	25	3.6	50	850	3	0.019	0.082
N15	11	101	0.188	338	10	8.9	70	850	18	0.113	0.564
N2	12	150	0.266	504	10	6	50	300	3	0.028	0.133
N1	13	51	0.100	171	10	17.5	50	300	4	0.013	0.067
N4	14	150	0.266	504	10	6	90	300	59	0.550	2.614
N20	15	101	0.163	844	25	3.6	70	1400	28	0.176	0.762
N7	16	51	0.100	171	10	17.5	50	1400	4	0.013	0.067
N23	17	101	0.163	844	25	3.6	70	850	22	0.138	0.598
N12	18	150	0.186	2000	40	1.5	90	1400	45	0.419	1.397
N19	19	101	0.163	844	25	3.6	70	300	25	0.157	0.680
N14	20	150	0.219	1260	25	2.4	70	850	12	0.112	0.437
N21	21	101	0.163	844	25	3.6	70	850	22	0.138	0.598
N22	22	101	0.163	844	25	3.6	70	850	22	0.138	0.598
N18	23	101	0.163	844	25	3.6	90	850	83	0.521	2.257

[a]Concentration was calculated using the total volume of aqueous and organic phase.

1.6 Recycling of aqueous phase in batch

1.6.1 Using water the aqueous phase

Methylisatin **1** (51 mg; 0.317 mmol) and nitromethane (0.42 mL; 7.925 mmol) were placed in a reaction vial (OD: 2.1 cm; H: 7 cm) with a PTFE septum lid. When methylisatin dissolved fully in nitromethane, 3 mL water was added and the reaction vial was placed in a custom-made aluminium heating block with 1 inch off-set stirring. The reaction was stirred using magnetic stirrer (1 cm x 1 cm cross-bar stirrer). Each reaction was left for 3 h to react at 70 °C. Due to issues with phase separation, the sample was left at room temperature with no stirring overnight, then aqueous phase was separated using 1 mL plastic syringe with a stainless steel needle. A sample of organic phase was dissolved in $CDCl_3$ and analyzed via ^1H NMR using ratios of aromatic signals at 7.6 ppm (methylisatin), 7.4 ppm (product), and 7.33 ppm (co-product, if observed).

Table S4. Results of recycling experiments when water was used as an aqueous phase

	Volume of water [mL]	^1H NMR yield [%]
Reaction 1/Recycle 0	3.0	97.0
Reaction 2/Recycle 1	2.85	96.0

Reaction 3/Recycle 2	2.4	95.5
Reaction 4/Recycle 3	2.25	95.5
Reaction 5/Recycle 4	1.8	94.0

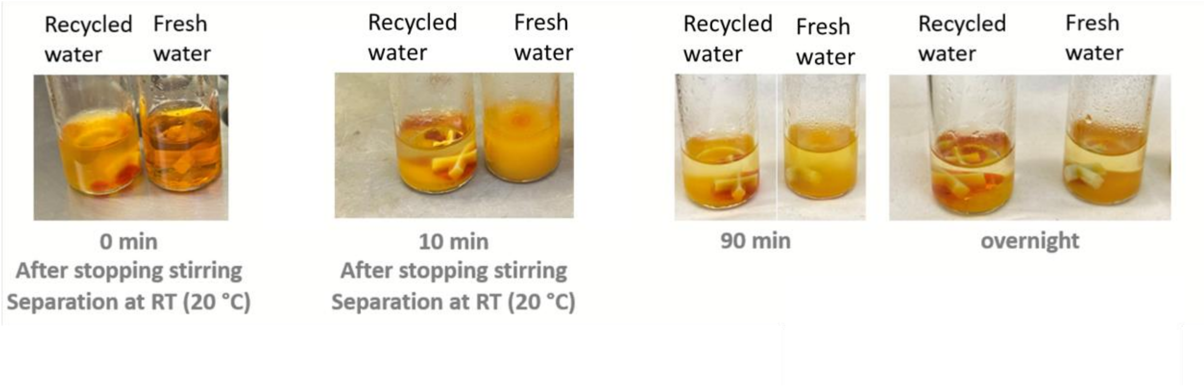

Figure S8. Phase separation over time with fresh and recycled water.

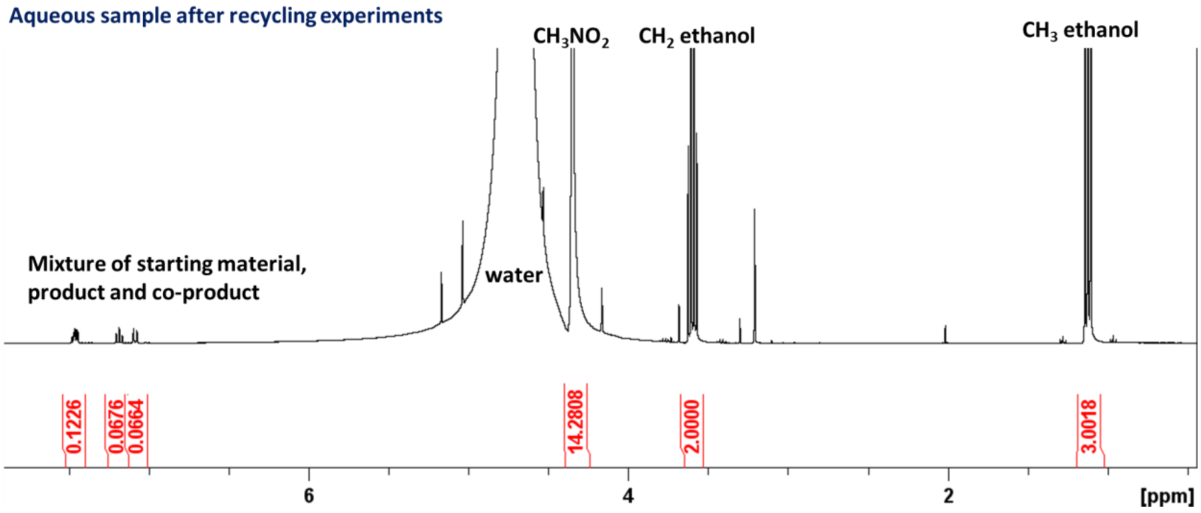

Figure S9. ^{1}H NMR spectrum of a recycled aqueous phase (after final recycling). This sample was prepared in D_2O with ethanol as internal standard (0.6 mL of aqeous sample from reaction + 0.2 mL D_2O with internal standard, to prepare D_2O with internal standard: 0.15 mL ethanol in 2.5 mL D_2O). A 0.6 mL sample of aqeous phase contained roughly 0.01 mmol of starting material/product mixture and 51 mg nitromethane.

1.6.2 Using 0.1 M phosphate pH 7 buffer as the aqueous phase

Methylisatin (51 mg; 0.317 mmol) and nitromethane (0.42 mL; 7.925 mmol) were placed in a reaction vial (D: 2.1 cm; H: 7 cm) with a PTFE septum lid. When methylisatin dissolved fully in nitromethane, 3 mL aqueous phosphate buffer was added and the reaction vial was placed in a custom-made aluminium heating block with 1 inch off-set stirring. The reaction was stirred using magnetic stirrer (1 × 1 cm cross-bar stirrer). Each reaction was left for 10 min to react at 25 °C, after the reaction was finished phases were left to separate at room temperature for the time

indicated in Table S5, the aqeous phase was separated using 1 mL plastic syringe with a stainless steel needle. A sample of organic phase was dissolved in $CDCl_3$ and analyzed via ^1H NMR.

Table S5. Results of recycling experiments when 0.1 M phosphate pH 7 buffer was used as an aqoues phase

	Volume of phosphate buffer [mL]	Separation time [min]	^1H NMR yield [%]
Reaction 1/Recycle 0	3	38 min	100
Reaction 2/Recycle 1	3	30 min	98.5
Reaction 3/Recycle 2	2.6	34 min	97.5
Reaction 4/Recycle 3	2.35	55 min	98.5
Reaction 5/Recycle 4	2.2	50 min	99.0

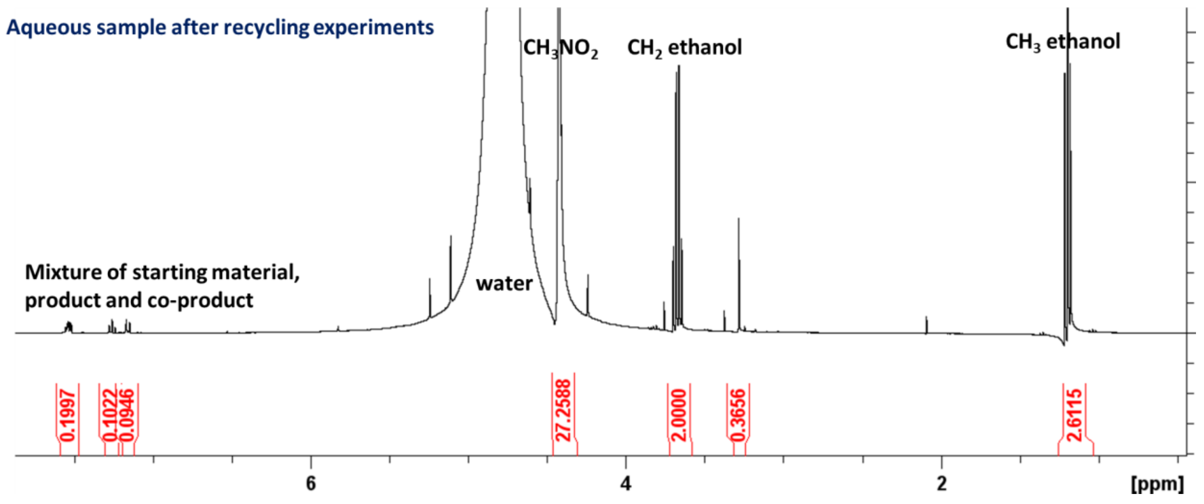

Figure S10. ^1H NMR spectrum of recycled 0.1 M phosphate pH 7 buffer (after final recycling). This sample was prepared in D_2O with ethanol as internal standard (0.6 mL of aqeous sample from reaction + 0.2 mL D_2O with internal standard, to prepare D_2O with internal standard: 0.15 mL ethanol in 5 mL D_2O). A 0.6 mL sample of aqeous phase contained roughly 0.01 mmol of starting material/product mixture and 52 mg nitromethane.

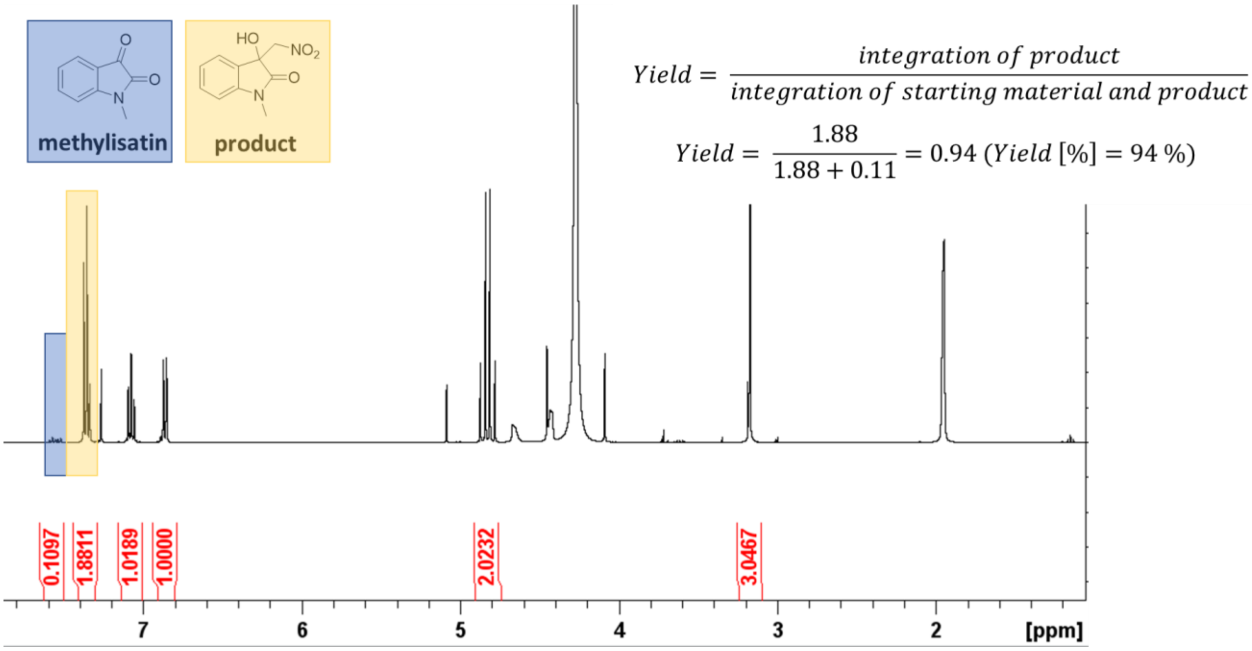

Figure S11. Example calculation of ^{1}H NMR yield using the ratio between aromatic peaks.

1.6.3 DSL measurement of aqueous phase from reaction using phosphate buffer

A reaction mixture using phosphate buffer was allowed to settle for 15 minutes after completion. The aqueous phase was directly transferred to a cuvette for DLS measurement. The results are summarized below.

Results

			Size (d.n...	% Intensity:	St Dev (d.n...
Z-Average (d.nm):	362.5	Peak 1:	316.1	76.2	74.17
PdI:	0.346	Peak 2:	0.8952	23.8	0.2452
Intercept:	0.621	Peak 3:	0.000	0.0	0.000

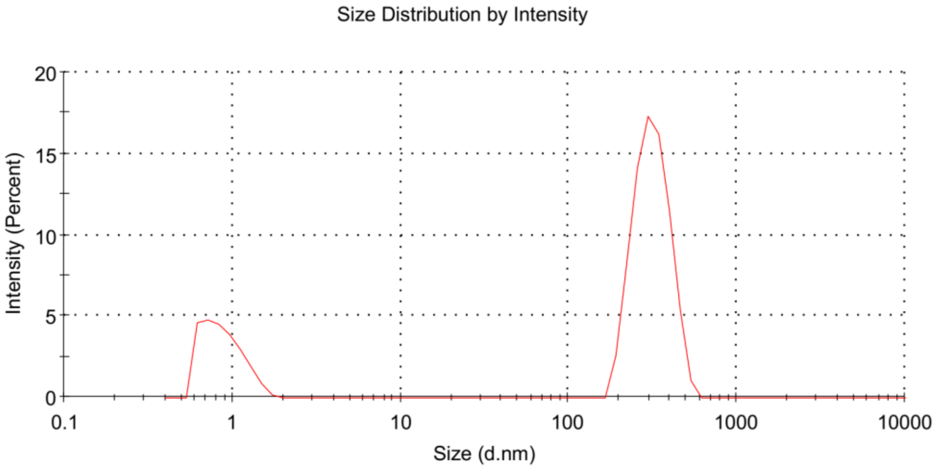

Figure S12. DLS measurement of the aqueous phase after reaction using phosphate buffer

1.7 Flow experiments and NMR analysis with trimethoxybenzene as an internal standard

Flow experiments were performed in a commercially available CSTR reactors (fReactors, Asynt) with a membrane separator connected to them (Zaiput SEP-10), the whole set-up is shown in the Figure S13.

The reactants were delivered to the reactors using syringe pumps, one syringe was filled with methylisatin and trimethoxybenzene (internal standard) dissolved in nitromethane; second syringe was filled with 0.1 M aqueous phosphate buffer. PTFE tubing was used for all connections (external diameter 1/8", internal diameter 1/16").

A cascade of three CSTR reactors was used, each reactor was equipped with a 1 cm x 1 cm cross bar stirrer, the overall volume 4.8 mL (excluding tubing between reactors). The reactors are made of PEEK, thus the temperature at a hot plate has to be set above the required reaction temperature. Additionally, temperature measurements (measured with a thermocouple type J and a hand held temperature reader, water was used instead of reaction medium) showed that there is about 3 °C gradient of temperature between 1st reactor in the cascade and the last one. The reaction temperature at the hot plate was set at 50 °C.

The efficiency of phase separation was tested for various aqueous to organic ratios, starting from 1:1 aqueous phase to organic, up to 7:1 ratio. For 1:1 ratio the best separation was achieved using hydrophobic membrane OB-900-S10 (if syringe pumps used) or OB-2000-S10 (piston pumps). Higher aqueous phase ratio required the use of a hydrophilic membrane, IL-900-S10 was used (tested only with syringe pumps).

Unless otherwise specified, the standard experimental conditions are: 0.48 mL/min overall flow rate (0.24 mL/min flow rate of each phase), 10 min residence time, 50 °C set on a hot plate, 850 rpm, 0.121 g methylisatin and 6 mg trimethoxybenzene in 1 mL nitromethane. Samples for analysis were taken from the organic phase of reaction after phase separation (about 2 drops of organic phase dissolved in about 0.6 mL $CDCl_3$), yields were calculated via ^{1}H NMR as described in Figure S13 and S14.

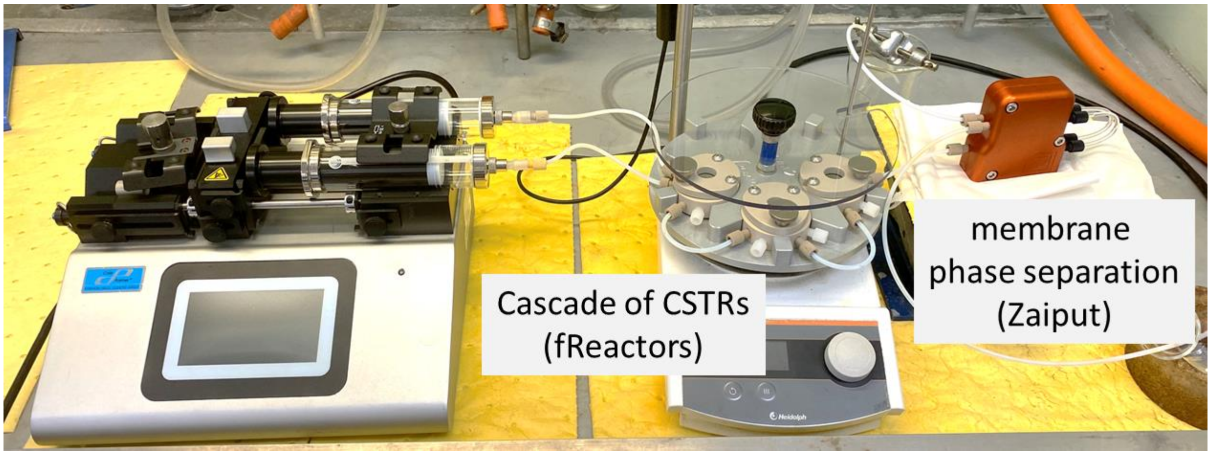

Figure S13. Experimental set up for flow experiments.

NMR analysis was performed using ^{1}H NMR spectrum with trimethoxybenzene (TMB) as an internal standard. Yields were calculated by referencing integrals agains sample at time 0 min, examples of calculations below.

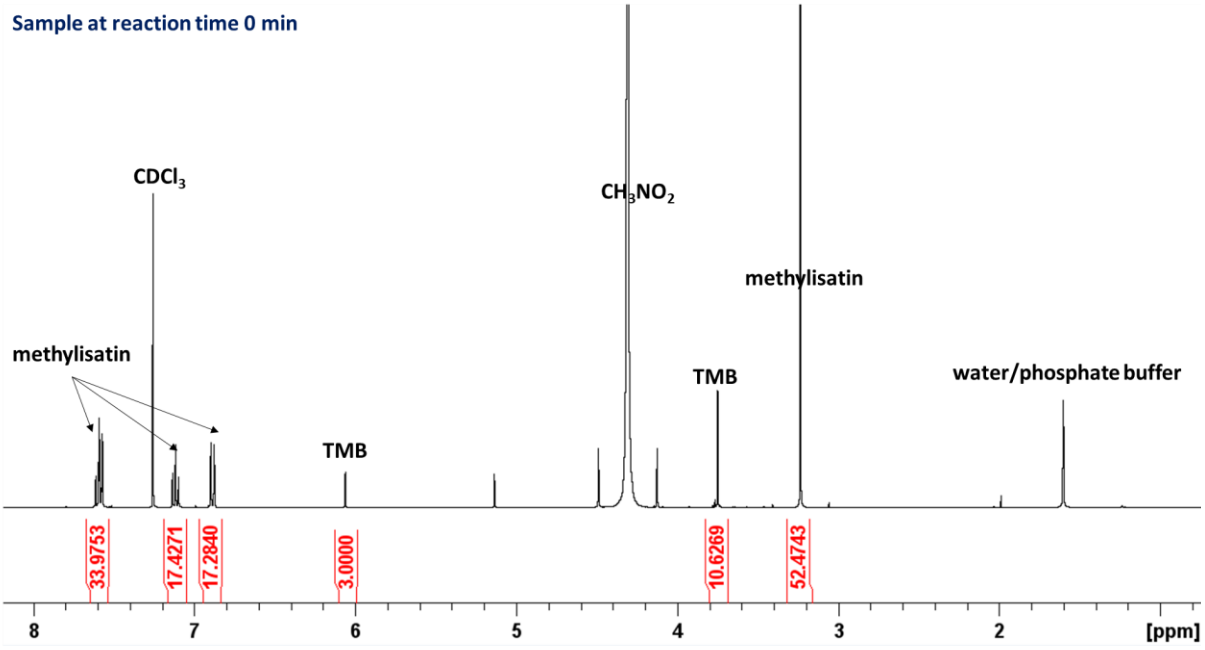

Figure S14. Example of ^{1}H NMR spectrum of reaction mixture at time = 0 min in $CDCl_3$.

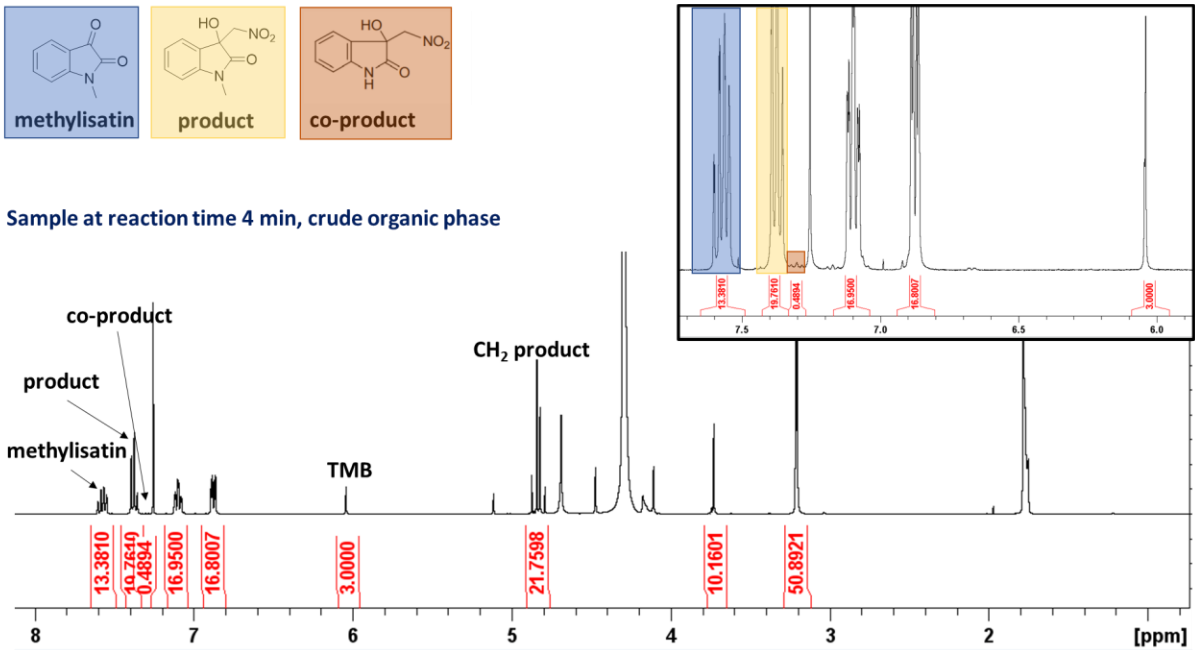

Figure S15. Example of ^{1}H NMR spectrum of the reaction mixture at time = 4 min in $CDCl_3$.

Calculations for sample at time 4 min:

$$Yield\ [molar\ fraction] = \frac{product\ integration\ in\ sample\ at\ time\ 4\ min}{methylisatin\ integration\ in\ sample\ at\ time\ 0\ min}$$

$$methylisatin = \frac{13.4}{34.0} = 0.39 \qquad product = \frac{19.8}{34} = 0.58 \qquad co-product = \frac{0.5}{34} = 0.015$$

$$mass\ balance = \frac{13.4 + 19.8 + 0.5}{34} = \frac{33.7}{34} = 0.99$$

Flow experiments with lower organic to aqueous ratios

A flow experimental set up with 3 CSTR (described above, Figure S12) was used to investigate the influence of lower organic to aqueous ratios on the reaction. Ratio 1:1, 1:2, and 1:3 were used, a table with residence times and flow rates is included below. While 1:1 ratio was investigated a hydrophobic membrane was used in Zaiput (OB-900-S10). For ratios 1:2 and 1:3 the membrane was changed for hydrophilic (IL-900-S10) to assure good phase separation. One syringe was loaded with a solution of methylisatin **1** in nitromethane with trimethoxybenzene (5 mol % in respect to **1**) as an internal standard, specifically 1.8 g methylisatin and 94 mg trimethoxybenzene was dissolved in 15 mL nitromethane. The second syringe was loaded with 0.1 M phosphate buffer, pH 7. A sample for analysis was taken from organic layer and dissolved in $CDCl_3$ for NMR analysis. The samples were analyzed using internal standard (examples of calculations in Figure S13 and S14) and without including the internal standard (examples of calculations in Figure S11). Lower organic to aqueous ratios (1:2 and 1:3) resulted in the loss of organic mass into water and if samples were analyzed using internal standard, it looked like the yield of reaction was decreasing (Figure S15). When the samples were analyzed without the use of internal standard, using ratios of aromatic peaks, the yield was the same (Figure S15, grey bar). This confirmed that the decrease of yield was caused by the disappearance of organic material into aqueous phase and not by changes to conversion or selectivity of the reaction. The yield presented is an average of three samples taken at steady state (at 25 min, 30 min, and 35 min of experiment which roughly corresponds to 2.5, 3, and 3.5 volumes of the reactor).

Ratio (organic : aqueous)	Residence time [min]	Overall flow rate [mL/min]	Flow rate of organic phase [mL/min]	Flow rate of aqueous phase [mL/min]
1:1	10	0.48	0.24	0.24
1:2	10	0.48	0.16	0.32
1:3	10	0.48	0.12	0.36

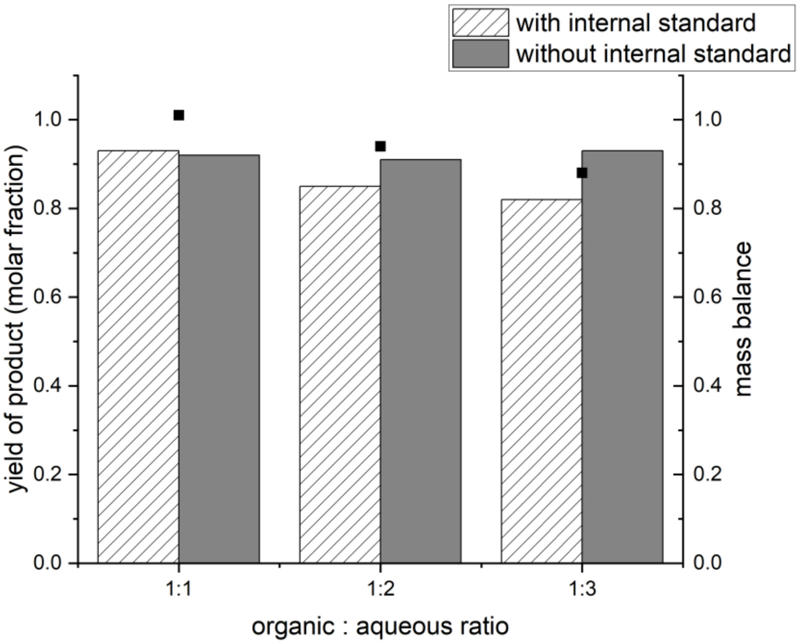

Figure S16. Results of flow experiments with different organic : aqueous phase ratios. The bars represent yield in steady state calculated with two methods (with and without internal standard). The black squeres represent mass balance.

1.8 Continous aqeuous phase recycling

Continuous aqueous phase recycling was performed for two experimental conditions (Method D and E in the Table 2 in the main manuscript), other parameters (temperature, stirring, phase separation) were kept the same as in Section 1.7:

1) Method D: 0.121 g methylisatin **1** in 1 mL nitromethane, residence time 10 min, pumps used: milliGAT for organic phase and ISMATEC MCP-CPF with RH0 CTC head for aqueous phase (data presented in Figure 7 in the main manuscript and Table S7 here). Overal flow rate 0.48 mL/min (0.24 mL/min on each syringe), the volume of recirculating loop and aqeous phase reservoir was 9 mL. Due to the fresh organic phase solubilizing aqueous phase, the aqueous phase had to be topped up with fresh phosphate buffer (at 4.5 h experiment 2 mL was added, at 8 h additional 1 mL was added, overall volume of aqueous phase used for this experiment was 12 mL).
2) Method E: 0.331 g methylisatin **1** in 1 mL nitromethane (solubility limit), residence time 20 min, pumps used: milliGAT for organic phase and KNF Simdos 02 FEM 1.02 TT for aqueous phase (data presented in Figure 7 in the main manuscript and Table S8 here) Overal flow rate 0.24 mL/min (0.12 mL/min on each syringe), the volume of recirculating loop and aqeous phase reservoir was 12 mL. Due to the fresh organic phase solubilizing aqueous phase, the aqueous phase had to be topped up with fresh phosphate

buffer (at 3.5 h experiment 0.5 mL was added, at 5.5 h additional 0.5 mL was added, overall volume of aqueous phase used for experiment 13 mL).

Before large scale flow experiments were conducted, an initial study in batch was done to determine the influence of methylisatin **1** concentration in nitromethane on reaction yield (Figure S16). The batch study confirmed zero order kinetics in methylisatin **1** and it was determined that 20 min residence time in flow should be sufficient to achieve full conversion.

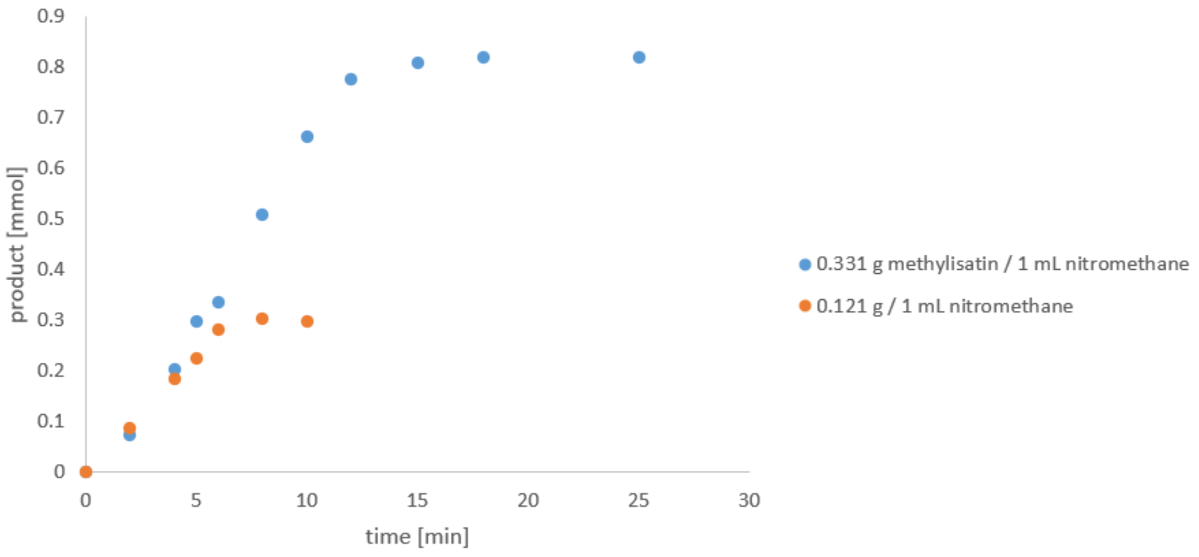

Figure S17. Reaction progress of batch experiments using 9 eq. (0.331 g of **1** / 1 mL of nitromethane) and 25 eq. (0.121 g of **1** / 1 mL of nitromethane) of $MeNO_2$. Reaction conditions: 40 °C, 850 rpm, 0.42 mL phosphate pH 7 buffer 0.1 M, 0.42 mL of premixed solution of methylisatin **1**, nitromethane and trimethoxybenzene (internal standard, 5 mol % in respect to methylisatin).

Table S6. Reaction progress of batch experiments using 9 eq. and 25 eq. of nitromethane (the same data presented in Figure S16)

time [min]	0.121 g **1** / 1 mL nitromethane			0.331 g **1** / 1 mL nitromethane		
	Methylisatin **1** [mmol]	Product **2** [mmol]	Co-product **4** [mmol]	Methylisatin **1** [mmol]	Product **2** [mmol]	Co-product **4** [mmol]
0	0.316	0	0	0.865	0	0
2	0.225	0.085	0.003	0.779	0.074	0.010
4	0.124	0.183	0.004	0.643	0.204	0.015
5	0.085	0.224	0.005	0.557	0.298	0.027
6	0.31	0.281	0.004	0.509	0.335	0.016
8	0.011	0.303	0.005	0.333	0.509	0.026
10	0.010	0.297	0.005	0.192	0.663	0.036
12				0.071	0.777	0.028
15				0.038	0.808	0.027

18				0.029	0.820	0.028
25				0.035	0.820	0.020

Table S7. Results of large scale aqueous phase recycling at concentration 0.121 g **1** / 1 mL nitromethane

time [h]	Molar fraction (based on ^{1}H NMR)			Mass balance
	Methylisatin **1**	Product **2**	Co-product **4**	
0	1	0	0	1
1	0.13	0.86	0.03	1.02
2	0.13	0.84	0.03	1.01
3	0.12	0.86	0.03	1.02
4	0.11	0.86	0.03	1.01
5	0.10	0.88	0.03	1.02
6	0.10	0.88	0.03	1.01
7	0.12	0.85	0.03	1.01
8	0.13	0.84	0.03	1.01
9	0.13	0.85	0.03	1.01
10	0.14	0.84	0.03	1.02
10.75	0.14	0.83	0.03	1.01

Table S8. Results of large scale aqueous phase recycling at concentration 0.331 g **1** / 1 mL nitromethane

time [h]	Molar fraction (based on ^{1}H NMR)			Mass balance
	Methylisatin **1**	Product **2**	Co-product **4**	
0	1	0	0	1
1	0.04	0.94	0.03	1.02
2	0.05	0.95	0.03	1.03
4	0.05	0.94	0.03	1.03
6	0.05	0.94	0.04	1.03
6.5	0.05	0.95	0.04	1.04
7	0.06	0.94	0.04	1.03

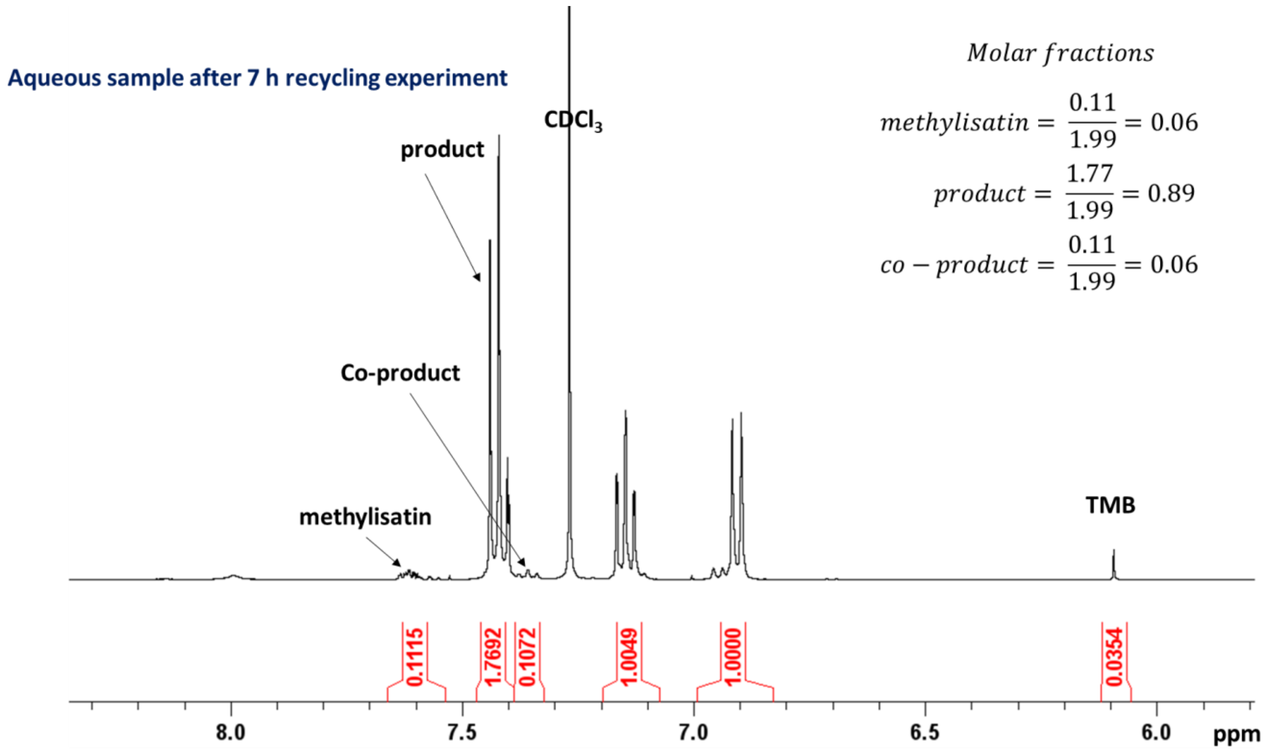

Figure S18. Qualitative analysis of the final aqueous sample from 7h recycling experiment with 0.331 g methylisatin / 1 mL nitromethane. To prepare sample, about 300 µL of aqueous phase was evaporated to dryness and redissolved in $CDCl_3$. TMB was detected in the sample, but it cannot be used for quantification as it does not dissolve in aqueous phase in the same ratio as nitromethane and **1**.

1.9 Measurements of solubility of $MeNO_2$

A mixture of 3.5 mL of nitromethane and 7 mL of water or 1M NaCl or 1M Na_2SO_4 was put in a 4-dram glass vial with 10 x 10 mm cross-shaped stir bar. The vial was placed on a magnetic stirrer, which was set at 850 rpm, the temperature was maintained with an aluminium heating block. The mixture was stirred for at least 45 minutes to equilibrate conditions in the vial, and measurements were taken at two temperatures: 25 and 70 °C. The resultant solubilities are an average of three samples and were calculated using ^1H NMR with a relaxation delay of 30s (Table S9). The obtained values of nitromethane solubility in water are within the same range as the previously reported values for nitromethane/water mixtures.[4,5]

Table S9. Nitromethane solubility in water, 1M NaCl and 1M Na_2SO_4 at 25 and 70 °C

Mixture	Solubility at 25 °C (% w/w)	Average solubility at 25 °C (% w/w)	Solubility at 70 °C (% w/w)	Average solubility at 70 °C (% w/w)
Nitromethane / water	11.1	11.3 ± 0.2	17.8	17.1 ± 0.7
	11.4		17.0	
	11.4		16.5	
Nitromethane / 1M NaCl	10.4	10.0 ± 0.6	13.3	12.9 ± 0.7
	10.3		13.4	
	9.4		12.1	
Nitromethane / 1M Na_2SO_4	5.4	5.6 ± 0.2	6.8	6.8 ± 0.2
	5.8		7.0	
	5.6		6.7	

General procedure of conducting measurements: a 150 µL aliquot of aqueous phase was taken and dissolved in 0.6 mL D_2O with ethanol as an internal standard (5 mL D_2O and 0.15 mL ethanol). The weight of 4-dram vial with D_2O before and after adding 150 µL aliquot was noted. The content of nitromethane in the aliquot was calculated using ^1H NMR with ethanol as an internal standard. Weight % (w %) was calculated using the ratio of the mass of nitromethane to the mass of 150 µL aliquot. Examples of an NMR spectrum and calculations are presented below.

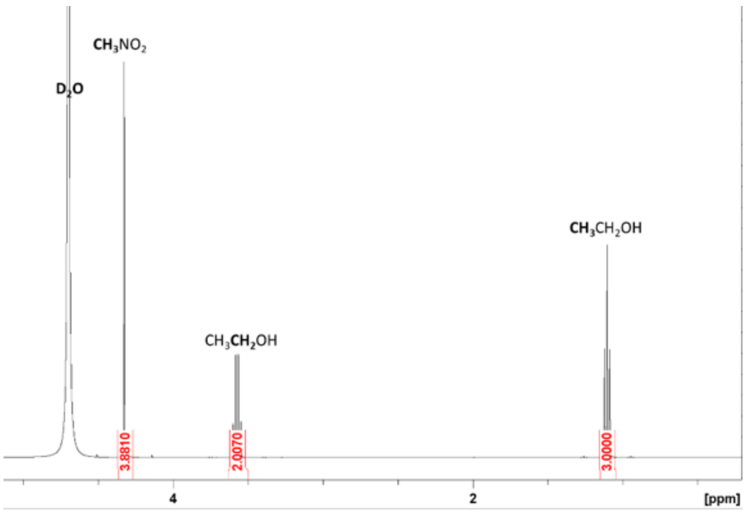

Figure S19. Example of ^{1}H NMR spectrum

$$\mathrm{Mass_{EtOH}}\ \text{(internal standard)in 0.6 mL}\ \mathrm{D_2O} = 12.94\ \mathrm{mg}$$

$$n_{\mathrm{EtOH}} = 0.281\ \mathrm{mmol}$$

$$n_{Nitromethane} = \frac{\text{integration of}\ \mathrm{CH_3NO_2} \times n_{\mathrm{EtOH}}}{number\ of\ protons\ in\ CH_3NO_2}$$

$$= \frac{3.9 \times 0.281\ \mathrm{mmol}}{3} = 0.365\ \mathrm{mmol}$$

$$\text{Mass of}\ \mathrm{CH_3NO_2} = 0.365\ \mathrm{mmol} \times 61\frac{\mathrm{mg}}{\mathrm{mmol}} = 22.3\ \mathrm{mg}$$

$$\text{Mass of aliquot} = 0.135\ \mathrm{g}$$

$$\text{Concentration (\% w/w)} = \frac{\mathrm{Mass_{nitromethane}}}{\text{Mass of aliquot}} \cdot 100\% = \frac{0.0223\ \mathrm{g}}{0.135\ \mathrm{g}} \cdot 100\% = 16.5\%$$

1.10 PMI-reaction calculations

$$\text{PMI-reaction} = \frac{mass\ of\ methylisatin + nitromethane + aqueous\ phase}{mass\ of\ product}$$

Note: Calculatations are based on reaction in steady state, the start up of reactor is not included

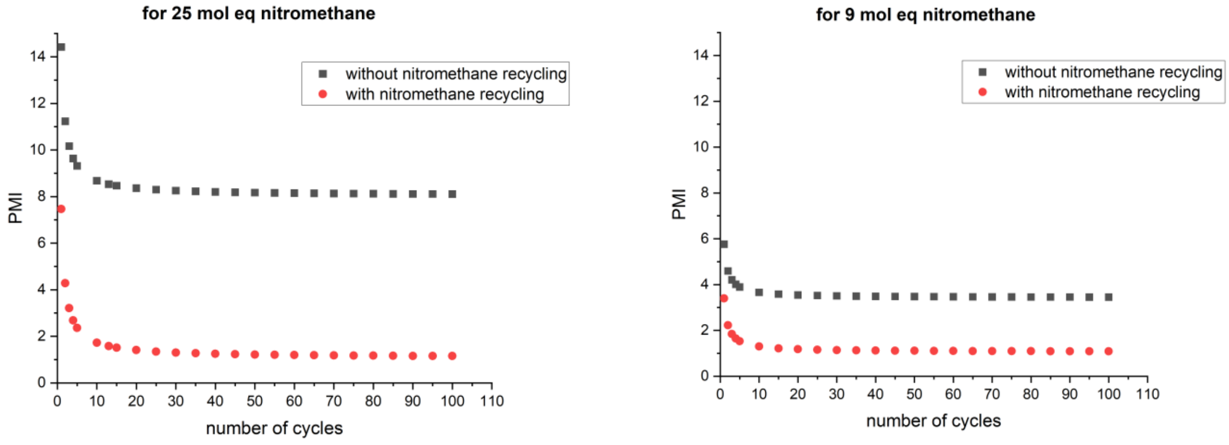

Figure S20. Theoretical PMI-reaction calculations for different flow systems *vs* number of cycles of reusing the aqueous phase, including and not inclucing nitromethane recycling

1.10.1 PMI-reaction literature value (Method A)[3]

Input: 80.5 mg methylisatin (0.5 mmol)
122 mg nitromethane
3000 mg water (3 mL)

Product: 98 mg

The paper reported 88 % isolated yield, for the purpose of calculations it was assumed that the same yield can be obtained via recrystallization, solvents for extraction and purification were not included in the calculations.

$$Mass\ of\ product = isolated\ yield\ \cdot mmol\ of\ methylisatin\ \cdot molecular\ weight\ of\ product$$

$$= 0.88\ \cdot 0.5\ mmol\ \cdot 222\frac{mg}{mmol} = 98\ mg$$

$$\text{PMI-reaction} = \frac{80.5+122+3000}{98} = 33$$

1.10.2 PMI-reaction using DoE protocol and recycling the aqueous phase for 5 cycles (Method B)

1.10.2.1 Using water as aqueous phase

Input: 5 x 51 mg = 0.255 g methylisatin
5 x 0.48 g (5 x 0.42 mL) = 2.4 g nitromethane
3 mL water

Product: 0.34 g

5 cycles, yield by ^{1}H NMR: 97 % yield, 96 %, 95.5 %, 95.5 %, and 94 %

$$Mass\ of\ product = (0.97\ \cdot 0.32 +\ 0.96\ \cdot 0.32 +\ 0.955\ \cdot 0.32 +\ 0.955\ \cdot 0.32 +\ 0.94\ \cdot 0.32)\ \cdot 222 \frac{mg}{mmol}$$
$$= (0.31 + 0.31 + 0.31 + 0.31 + 0.30) \cdot 222 \frac{mg}{mmol} = 342\ mg = 0.34\ g$$

$$\text{PMI-reaction} = \frac{0.255+2.4+3}{0.34} = 16.6$$

1.10.2.2 Using 0.1 M phosphate buffer pH 7 as the aqueous phase

Input: 5 x 51 mg = 0.255 g methylisatin

5 x 0.48 g (5 x 0.42 mL) = 2.4 g nitromethane

3 mL aqueous phosphate buffer, 0.1 M

Product: 0.35 g

5 cycles, yield by ^{1}H NMR: 100 %, 98.5 %, 97.5 %, 98.5 %, and 99 %

$$Mass\ of\ product = (1\ \cdot 0.32 +\ 0.985\ \cdot 0.32 +\ 0.975\ \cdot 0.32 +\ 0.985\ \cdot 0.32 +\ 0.99\ \cdot 0.32)\ \cdot 222 \frac{mg}{mmol}$$
$$= (0.32 + 0.32 + 0.31 + 0.32 + 0.32) \cdot 222 \frac{mg}{mmol} = 353\ mg = 0.35\ g$$

$$\text{PMI-reaction} = \frac{0.255+2.4+3}{0.35} = 16.2$$

1.10.3 PMI-reaction for flow with reduced amount of aqueous phase, no aqueous phase recycling (Method C)

Input: 51 mg methylisatin (0.32 mmol)

480 mg nitromethane

420 mg aqueous phase (0.1 M phosphate buffer, d = 1 g/mL)

Product: 66 mg

93 % yield (by ^{1}H NMR), after evaporation of nitromethane the crude reaction product is obtained as light orange solid (the orange colour comes from unreacted methylisatin)

$$Mass\ of\ product = 0.93\ \cdot 0.32\ \cdot 222 \frac{mg}{mmol} = 66\ mg$$

$$\text{PMI-reaction} = \frac{51+480+420}{66} = 14$$

1.10.4 PMI-reaction for flow with aqueous phase recycling (Method D)

Input: 18.7 g methylisatin (116 mmol)

176.5 g nitromethane 154.8 mL; d = 1.14 g/mL

12 g aqueous phase (0.1 M phosphate buffer, d = 1 g/mL)

Product: 21.9 g

85 % yield (by ^{1}H NMR), after evaporation of nitromethane the crude reaction product is obtained as light orange solid (the orange colour comes from unreacted methylisatin)

$$Mass\ of\ product = 0.85 \cdot 116 \cdot 222 \frac{mg}{mmol} = 21.9\ g$$

$$\text{PMI-reaction} = \frac{18.7+176.5+12}{21.9} = 9.5$$

1.10.5 PMI-reaction for flow with increased lower nitromethane:1 ratio (Method E)

Input: 16.6 g methylisatin (103 mmol)

57 g nitromethane

13 g aqueous phase (0.1 M phosphate buffer, d = 1 g/mL)

Product: 21.5 g

94 % yield (by ^{1}H NMR), after evaporation of nitromethane the crude reaction product is obtained as light orange solid (the orange colour comes from unreacted methylisatin)

$$Mass\ of\ product = 0.94 \cdot 103 \cdot 222 \frac{mg}{mmol} = 21.5\ g$$

$$\text{PMI-reaction} = \frac{16.6+57+13}{21.5} = 4$$

1.11 Space-time-yield calculations

$$STY = \frac{the\ amount\ of\ product}{volume\ of\ the\ system\ \cdot time}$$

Note: Calculatations are based on reaction in steady state, the start up of reactor is not included

1.11.1 Experiments with 25 eq. of nitromethane

Concentration of starting solution: 0.121 g / 1 mL nitromethane

In mmol/mL: 0.75 mmol/1 mL

$$concentration\ \left[\frac{mmol}{mL}\right] = concentration\ \left[\frac{g}{mL}\right] \cdot \frac{1}{molecular\ weight} \cdot 1000 = 0.121\ \cdot \frac{1}{161} \cdot 1000 = \ 0.75\ mmol/mL$$

Yield (molar fraction) 0.94

The amount of product 0.705 mmol / 1 mL

$$the\ amount\ of\ product\ \left[\frac{mmol}{mL}\right] = yield\ [molar\ fraction] \cdot concentration\ of\ starting\ material\ \left[\frac{mmol}{mL}\right] = 0.94\ \cdot 0.75 \frac{mmol}{mL} = 0.705 \frac{mmol}{mL}$$

The amount of product in g / mL: 0.156 g / mL

$$concentration\ \left[\frac{g}{mL}\right] = concentration\ \left[\frac{mmol}{mL}\right] \cdot \frac{molecular\ weight\ \left[\frac{mg}{mmol}\right]}{1000} = 0.705\ \cdot \frac{222}{1000} = \ = \ 0.156\ g/mL$$

Flow rate of organic: 0.24 mL/ min (residence time 10 min)

Assume time 1 min

$$STY = \frac{concentration\ of\ product\ \left[\frac{g}{mL}\right] \cdot volume\ flown\ within\ assumed\ time}{volume\ of\ reactor \cdot time}$$

$$STY = \frac{0.156 \frac{g}{mL}\ \cdot 0.24\ mL}{4.8\ mL \cdot 1\ min} = 0.0078\ \frac{g}{mL\ \cdot min} = 0.468\ \frac{g}{mL\ \cdot h} = 468\ \frac{g}{L\ \cdot h} = 0.47\ \frac{kg}{L\ \cdot h}$$

1.11.2 Experiments with 9 eq. of nitromethane

Concentration of starting solution: 0.331 g / 1 mL nitromethane

In mmol/mL: 2.05 mmol/1 mL

$$concentration\ \left[\frac{mmol}{mL}\right] = concentration\ \left[\frac{g}{mL}\right] \cdot \frac{1}{molecular\ weight} \cdot 1000 = 0.331\ \cdot \frac{1}{161} \cdot 1000 = \ 2.05\ mmol/mL$$

Yield: 94%

The amount of product 1.93 mmol / 1 mL

$$the\ amount\ of\ product\ \left[\frac{mmol}{mL}\right]$$

$$= yield\ [molar\ fraction] \cdot concentration\ of\ starting\ material\ \left[\frac{mmol}{mL}\right]$$

$$= 0.94\ \cdot 2.05 \frac{mmol}{mL} = 1.93\ \frac{mmol}{mL}$$

The amount of product in g / mL: 0.428 g / mL

$$concentration\ \left[\frac{g}{mL}\right] = concentration\ \left[\frac{mmol}{mL}\right] \cdot \frac{molecular\ weight\ \left[\frac{mg}{mmol}\right]}{1000} = 1.93\ \cdot \frac{222}{1000} =$$

$$= \ 0.428\ g/mL$$

Flow rate of organic: 0.12 mL/ min (residence time 20 min)

Assume time 1 min

$$STY = \frac{concentration\ of\ product\ \left[\frac{g}{mL}\right] \cdot volume\ flown\ within\ assumed\ time}{volume\ of\ reactor \cdot time}$$

$$STY = \frac{0.428 \frac{g}{mL}\ \cdot 0.12\ mL}{4.8\ mL \cdot 1\ min} = 0.0107\ \frac{g}{mL\ \cdot min} = 0.642\ \frac{g}{mL\ \cdot h} = 642 \frac{g}{L\ \cdot h} = \ 0.64 \frac{kg}{L\ \cdot h}$$

1.12 PMI-reaction and STY calculations for literature examples

Note: All examples are batch reactions. The organic solvents used for for purification was not included, as in small scale reaction these tends to be used excessively. Thus, the green metrics we calculated for litereature protocols represent their best-case-scenario without taking into account purification stage.

1.12.1 Example 1

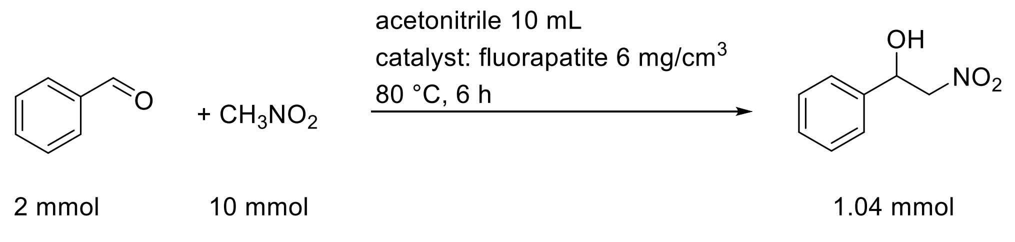

Figure S21 Literture example of a Henry reaction using fluorapatite as a catalyst.[6] Conversion 65 %, Selectivity 80 %.

General procedure: *Henry reaction of nitromethane and benzaldehyde was per- formed in 50 mL glass reactor equipped with a 6-blade pitched turbine impeller. The reactor was immersed in an oil bath with PID controller to*

maintain the temperature of the oil bath. 10 mmol of nitromethane (0.6 g) was reacted with 2 mmol of ben-zaldehyde (0.2 g) in the presence of 0.006 g/cm3 catalyst (0.06 g) and 10 mL of acetonitrile, 1000 rpm at 323 K for 6 h. The initial sample was collected when the reaction reached the desired temperature. 0.1 mL of samples were taken out at fixed inter- vals up to 6 h and centrifuged to separate the catalyst particles."[6]

The highest yielding reaction was chosen for calculations, at 80 °C, Table 1 in the original paper.

	Starting material	Nitromethane	Solvent	Catalyst	Product
mmol	2 mmol	10 mmol	-	-	1.04
MW	106	61	-	-	167
mass	212 mg	610 mg	7.9 g (10 mL, density: 0.79 g/cm^3	60 mg (assuming 10 mL overall volume)	174 mg

$$PMI = \frac{0.610 + 0.212 + 0.060 + 7.9}{0.174} = 50$$

$$STY = \frac{0.174\ g}{10\ mL\ \cdot 6\ h} = 0.0029\ \frac{g}{mL\ \cdot h} = 0.0029\ \frac{kg}{L\ \cdot h}$$

1.12.2 Example 2

General procedure: *"To the reaction vessel containing α-Keto Amide (0.2 mmol), nitromethane (4.0 mmol), arginine (20 mol%) and water 2.0 mL was added, and the reaction mixture was vigorously stirred at room temperature. After the complete consumption of starting material as monitored by thin layer, the mixture was extracted with DCM (30 mL × 3), combined organic phases and concentrated, then chromatography using ethyl acetate and hexane as eluent (1:6) to afford pure products."*[7]

a) Using organic base, the highes yield was chosen

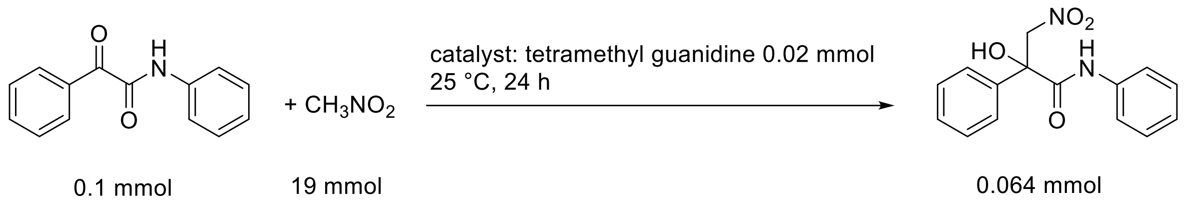

Figure S22 Literature example of a Henry reaction using organic base (tetramethyl guanidine) as a catalyst.[7] 64 % yield.

The highest yielding reaction catalyzed by an organic base was chosen, Table 1 Entry 3 in the original publication.

	Starting material	Nitromethane	Solvent	Catalyst	Product
mmol	0.1 mmol	19 mmol	-	0.02 mmol	0.064
MW	225	61	-	115	286
mass	22.5 mg	1.14 g (1 mL, density: 1.14 g/mL)	-	2.3 mg	18 mg

$$PMI = \frac{0.023 + 0.002 + 1.14}{0.018} = 65$$

$$STY = \frac{0.018\ g}{1\ mL\ \cdot 24\ h} = 7.5\ \cdot\ 10^{-4}\ \frac{g}{mL\ \cdot h} = 7.5\ \cdot\ 10^{-4}\ \frac{kg}{L\ \cdot h}$$

b) Using L- Arginine, the highest yield was chosen

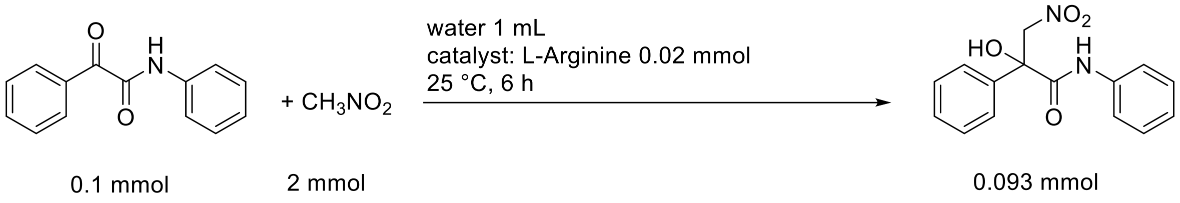

Figure S23 Literature example of a Henry reaction using L-Arginine as a catalyst. 93 % yield.[7]

The highest yield reaction was chosen for the calculations, Table 1 Entry 10 in the original paper.

	Starting material	Nitromethane	Solvent	Catalyst	Product
mmol	0.1 mmol	2 mmol	-	0.02 mmol	0.093
MW	225	61	-	174	286
mass	22.5 mg	122 mg	1 g (1 mL water)	3 mg	27 mg

$$PMI = \frac{0.023 + 0.003 + 0.122 + 1}{0.027\ g} = 43$$

$$STY = \frac{0.027\ g}{1.1\ mL\ \cdot 6\ h} = 0.004\ \frac{g}{mL\ \cdot h} = 0.004\ \frac{kg}{L\ \cdot\ h}$$

1.12.3 Example 3

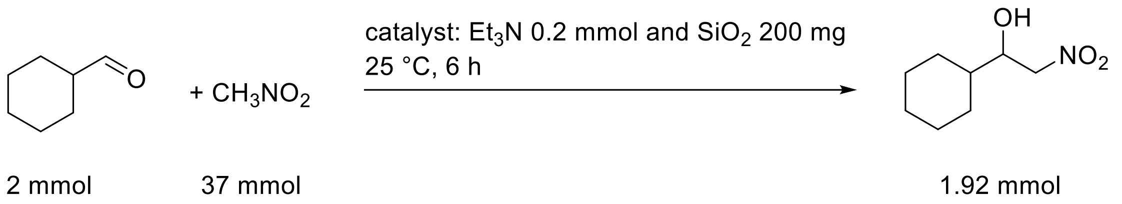

Figure S24 Literature example of a Henry reaction using triethylamine and silicon dioxide as catalysts.[8] 96 % yield.

General procedure: *"Triethylamine (20 mg, 0.2 mmol) was added to a mixture of 1a (268 mg, 2.0 mmol) and silica gel (200 mg) in nitromethane (2 mL). The mixture was stirred at room temperature for 2 h. Silica gel was filtered and washed with EtOAc. After 10% aqueous citric acid solution (2 mL) was added to the solution, the mixture was extracted with EtOAc three times and the combined organic layer was dried. Evaporation of the solution at 40 °C gave a residue which was purified by column chromatography on silica gel using heptane–acetone (4:1) as eluent to afford 2a (386 mg, 99%)."*[8]

The reaction chosen for calculations: Table 2 Entry 8 in the original paper.

	Starting material	Nitromethane	Catalyst 1	Catalyst 2	Product
mmol	2 mmol	37	-	0.2	1.92
MW	101	61	-	101	162
mass	202 mg	2.28 g (2 mL, density: 1.14 g/mL)	200 mg (SiO_2)	20.2 mg	311 mg

$$PMI = \frac{0.202 + 0.020 + 2.28 + 0.200}{0.311} = 8.7$$

$$STY = \frac{0.311\ g}{2\ mL \cdot 6\ h} = 0.026 \frac{g}{mL \cdot h} = 0.026\ \frac{kg}{L \cdot h}$$

2 Comparing molecular modelling techniques for known water-accelerated reactions

2.1 General procedure

PM6-D3H4 calculations were carried out using MOPAC 2016 package.[9] The large systems resulted in multiple imaginary frequencies and attempts to improve through ultrafine grid and tighter SCF condition were unsuccessful. The standard COSMO solvation model in MOPAC2016 was used.

All other quantum mechanical calculations were performed with Gaussian 09 Rev D.01 on the High-Performance Research Computing facility at University of Leeds.[10] Geometries were optimized using two different density functionals and two different basis sets, with GD3 empirical dispersion correction where available. Polarisable continuum solvation model (PCM) solvation model was employed for toluene, ethanol, and nitromethane where appropriate. M06-2X calculations used the ultrafine integration grid. Vibrational frequencies were computed for all optimized structures to verify that they were either minima (zero imaginary frequencies) or transition states (a single imaginary frequency). Computationally obtained structures were illustrated with PyMOL or Avogadro. Natural bonding orbitals and partial charges were performed using NBO3 in Gaussian 09 Rev D.01.

2.2 Cycloaddition reaction

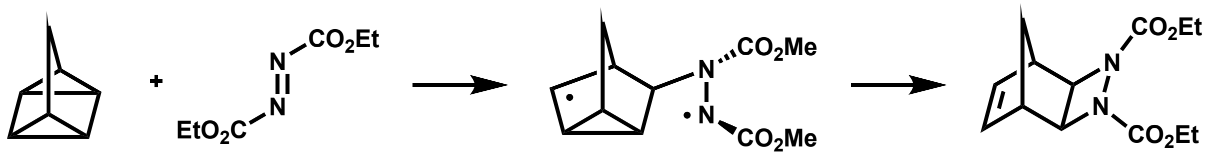

Optimized structures and energies of all stationary points. Absolute energies depicted in Hartrees.

2.2.1 PM6-D3H4 calculations in toluene

2.2.1.1 Reactant

Quadricyclane

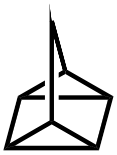

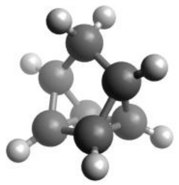

G (PM6-D3H4) kcal/mol

54.63

Dimethylazodicarboxylate

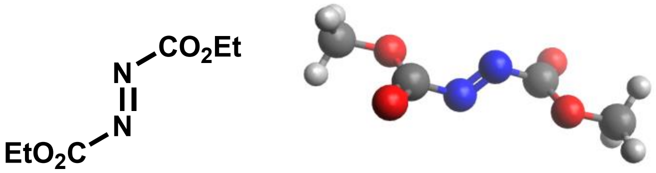

G (PM6-D3H4) kcal/mol
-141.62

2.2.1.2 Transition State

TS

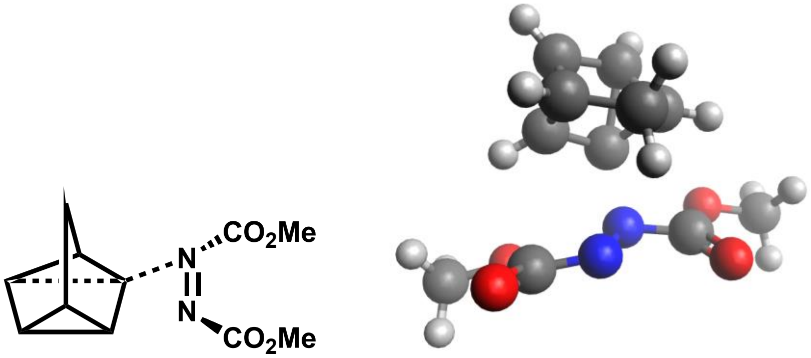

G (PM6-D3H4) kcal/mol
-44.34

2.2.1.3 Product

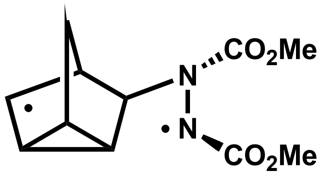

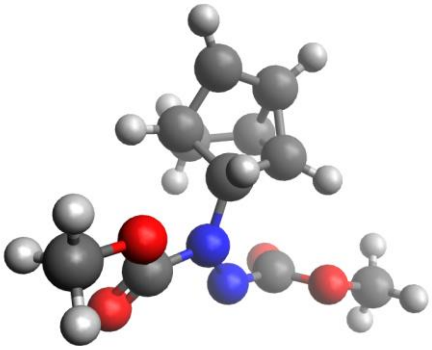

G (PM6-D3H4) kcal/mol
-94.37

2.2.2 PM6-D3H4 calculation with explicit H_2O molecules in toluene

2.2.2.1 Reactant

Dimethylazodicarboxylate

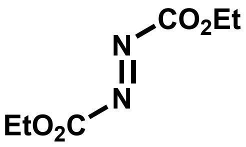

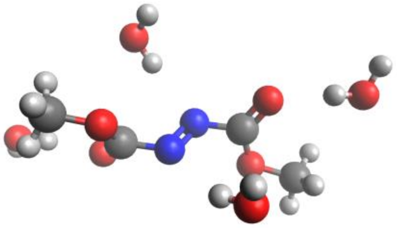

G (PM6-D3H4) kcal/mol
-399.42

2.2.2.2 Transition State

TS

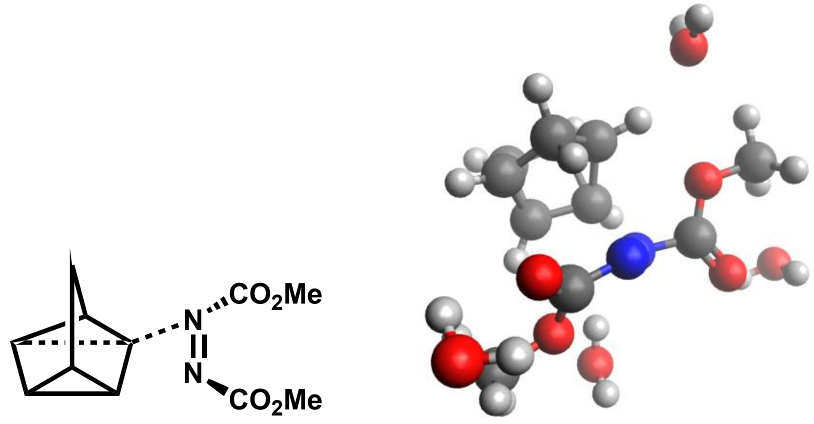

G (PM6-D3H4) kcal/mol
-300.73

2.2.3 B3LYP within the CPCM model for toluene

2.2.3.1 Reactant

Quadricyclane

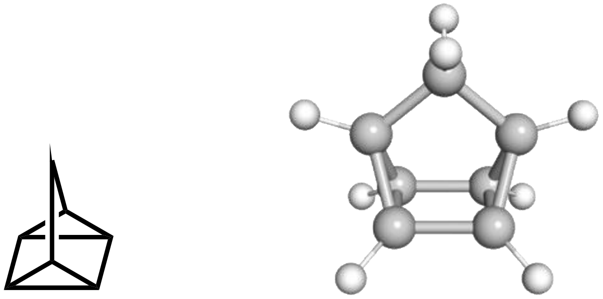

E (B3LYP)	H (B3LYP)	G (B3LYP)
-271.342039	-271.341095	-271.373918

Dimethylazodicarboxylate

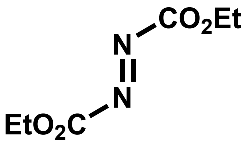

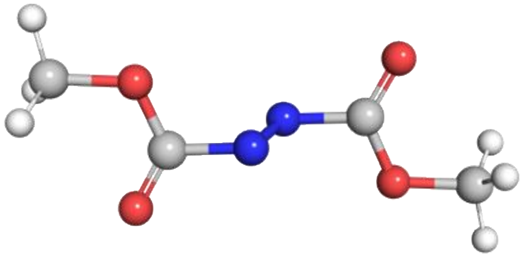

E (B3LYP)	H (B3LYP)	G (B3LYP)
-566.303127	-566.302182	-566.353568

2.2.3.2 Transition State

TS

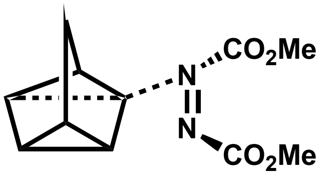

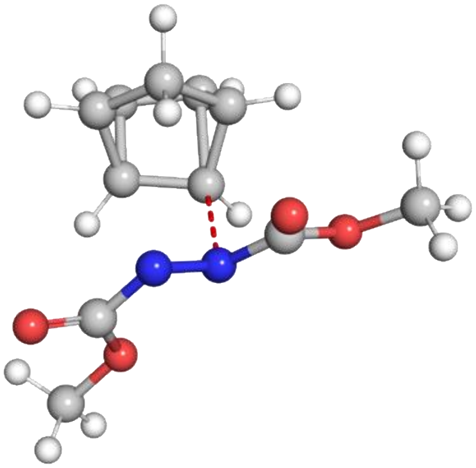

Imaginary frequency at -492.7 cm^{-1}

E (B3LYP)	H (B3LYP)	G (B3LYP)
-837.623256	-837.622312	-837.684288

2.2.3.3 Product

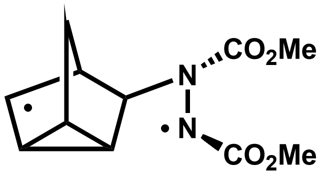

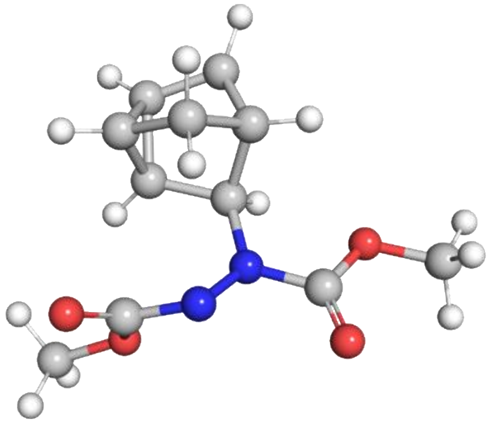

E (B3LYP)	*H (B3LYP)*	*G (B3LYP)*
-837.666555	-837.665610	-837.728114

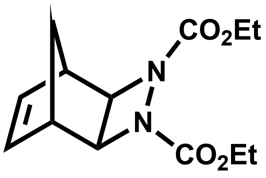

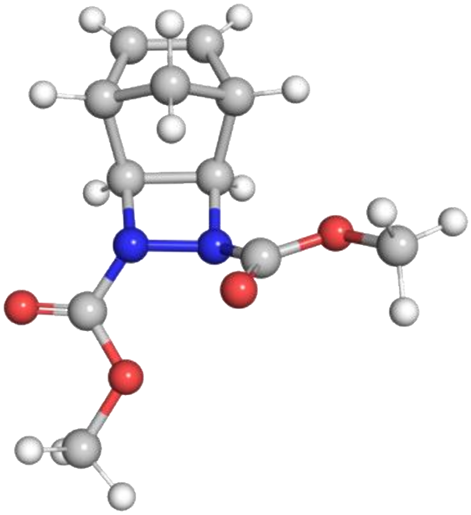

E (B3LYP)	*H (B3LYP)*	*G (B3LYP)*
-837.747636	-837.746692	-837.805939

2.2.4 B3LYP with explicit H_2O within the CPCM model for toluene

2.2.4.1 Reactant

Dimethylazodicarboxylate

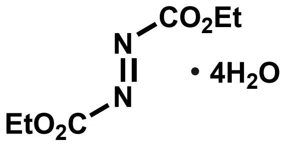

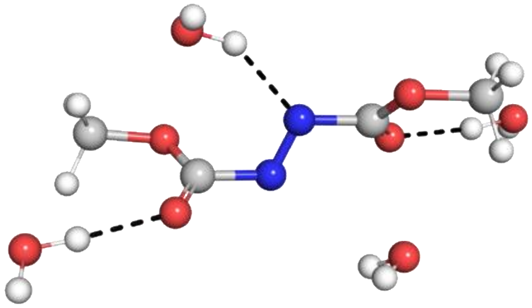

E (B3LYP)	*H (B3LYP)*	*G (B3LYP)*
-871.980283	-871.979339	-872.066827

2.2.4.2 Transition State

TS$_w$

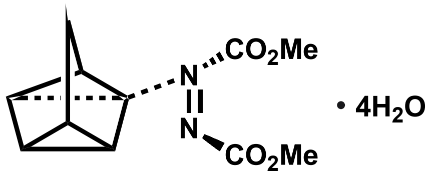

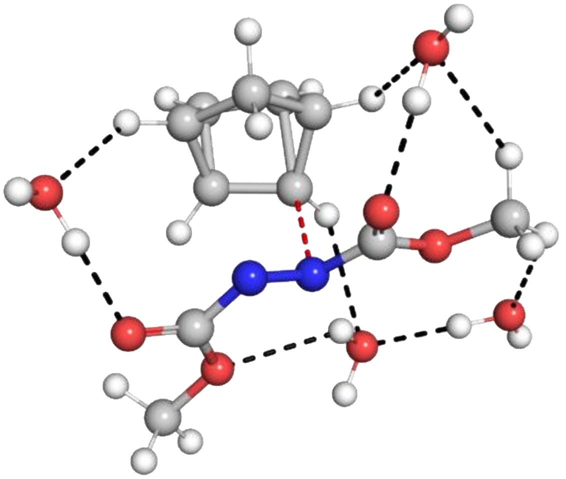

Imaginary frequency at -402.6 cm^{-1}

E (B3LYP)	*H (B3LYP)*	*G (B3LYP)*

-1143.322459	-1143.321515	-1143.414461

2.2.5 M06-2X within the CPCM model for toluene

2.2.5.1 Reactant

Quadricyclane

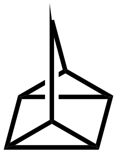

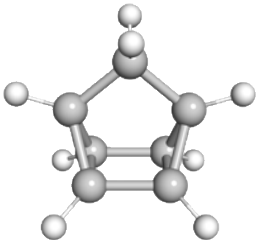

E (M06-2X)	*H (M06-2X)*	*G (M06-2X)*
-271.005854	-271.004910	-271.037633

Dimethylazodicarboxylate

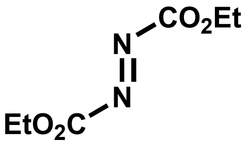

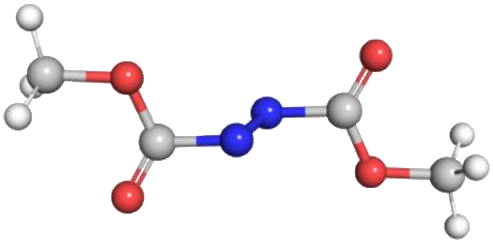

E (M06-2X)	*H (M06-2X)*	*G (M06-2X)*
-565.605789	-565.604845	-565.652708

2.2.5.2 Transition State

TS

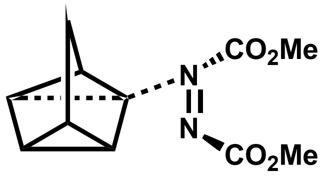

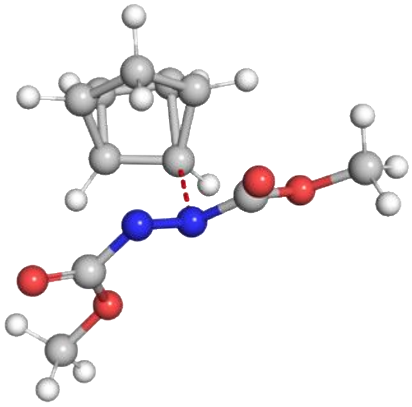

Imaginary frequency at -636.9 cm^{-1}

E (M06-2X)	*H (M06-2X)*	*G (M06-2X)*
-836.579510	-836.578566	-836.639399

2.2.5.3 Product

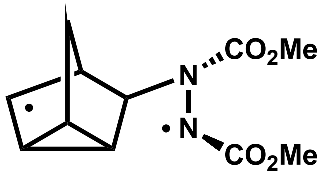

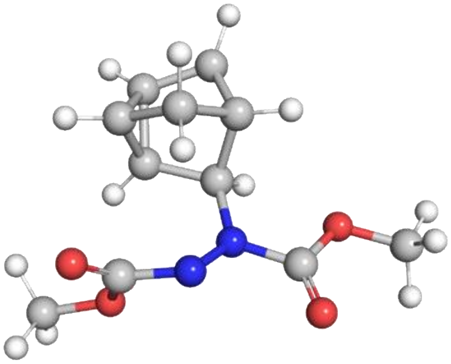

E (M06-2X)	*H (M06-2X)*	*G (M06-2X)*
-836.635342	-836.634398	-836.694794

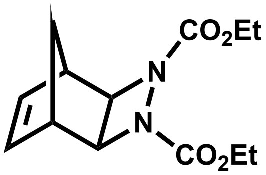

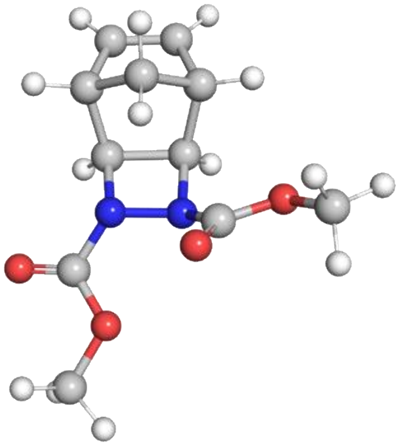

E (M06-2X)	H (M06-2X)	G (M06-2X)
-836.715969	-836.715025	-836.773017

2.2.6 M06-2X with explicit H_2O within the CPCM model for toluene

2.2.6.1 Reactant

TS$_w$

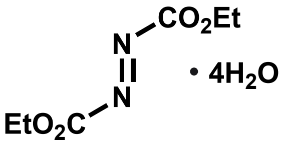

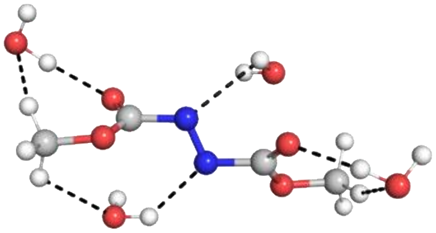

E (M06-2X)	H (M06-2X)	G (M06-2X)
-870.856799	-870.855855	-870.934749

2.2.6.2 Transition State

TS$_w$

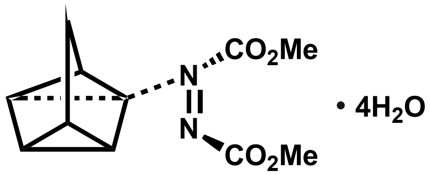

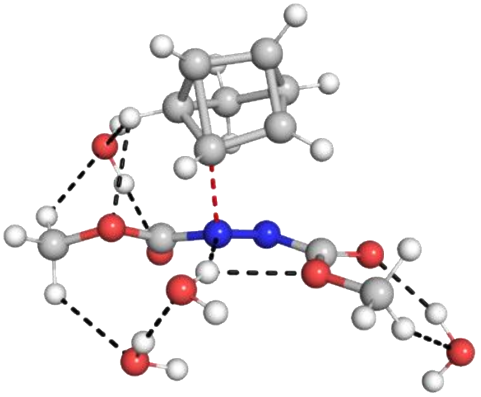

Imaginary frequency at -579.7 cm^{-1}

E (M06-2X)	*H (M06-2X)*	*G (M06-2X)*
-1141.847440	-1141.846496	-1141.932815

2.2.7 wB97X-D/def2-TZVP within the SMD model for toluene

2.2.7.1 Reactants

Quadricyclane

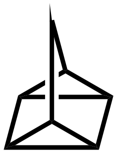

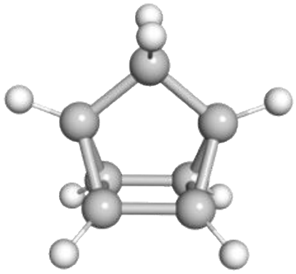

E	*H*	*G*
-271.337563	-271.336619	-271.370027

Dimethylazodicarboxylate

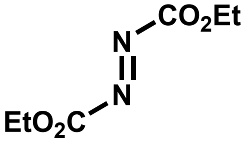

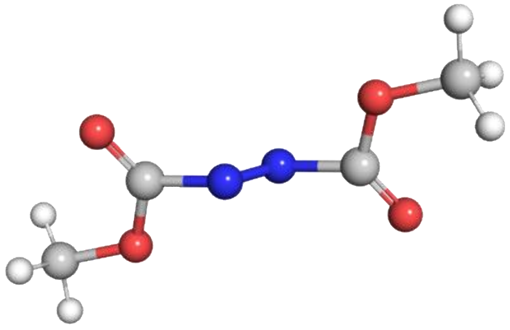

E	*H*	*G*
-566.306400	-566.305455	-566.355306

2.2.7.2 Transition State

TS

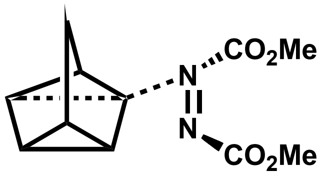

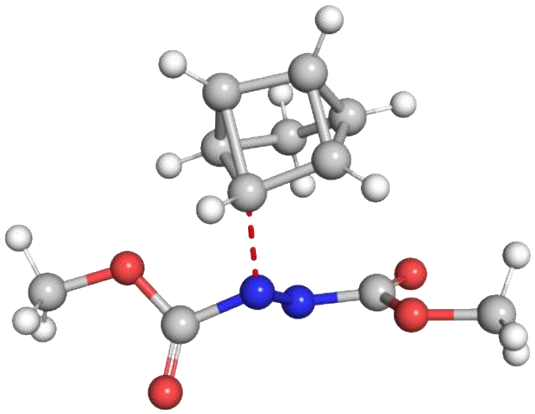

Imaginary frequency at -636.4 cm^{-1}

E	*H*	*G*
-837.601383	-837.600439	-837.660575

2.2.7.3 Product

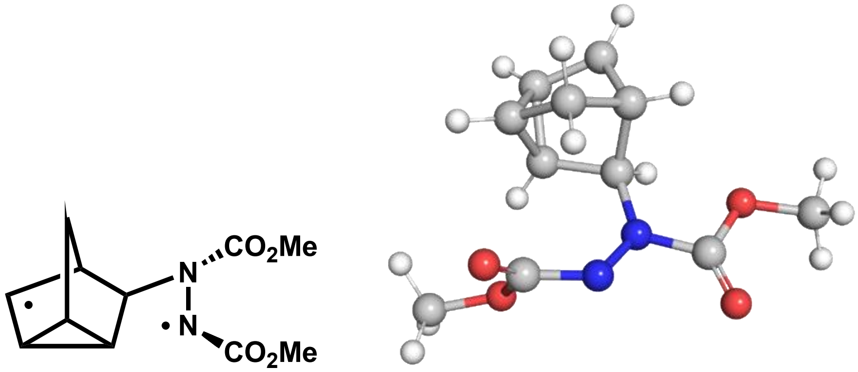

E	*H*	*G*
-837.657055	-837.656110	-837.718238

2.2.8 wB97X-D/def2-TZVP with explicit H_2O within SMD model for toluene

2.2.8.1 Reactant

Dimethylazodicarboxylate

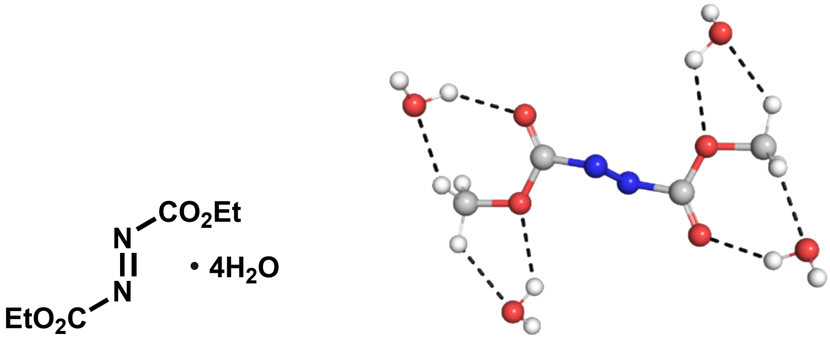

E	*H*	*G*
-872.008416	-872.007472	-872.082117

2.2.8.2 Transition State

TS$_w$

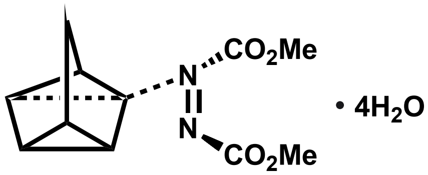

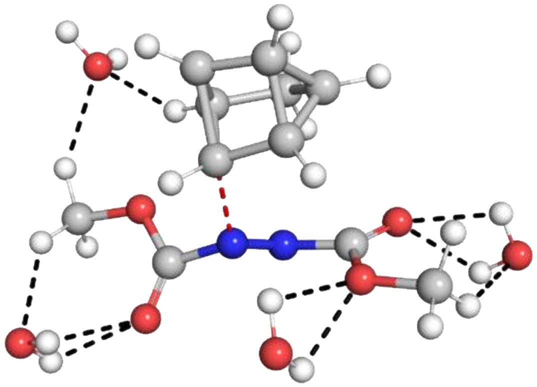

Imaginary frequency at -576.7 cm^{-1}

E	*H*	*G*
-1143.302935	-1143.301990	-1143.395031

2.2.9 wB97X-D/ma-def2-TZVP within the SMD model for toluene

2.2.9.1 Reactant

Quadricyclane

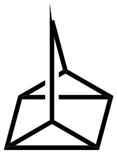

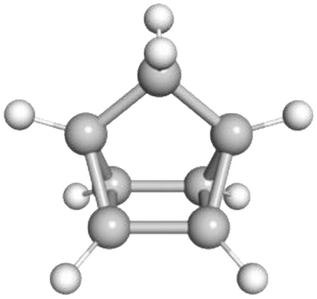

E	*H*	*G*
-271.337753	-271.336809	-271.370219

Dimethylazodicarboxylate

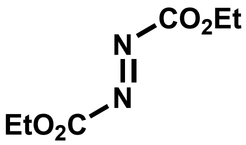

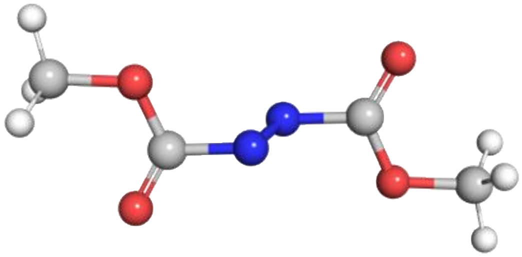

E	*H*	*G*
-566.308479	-566.307535	-566.357414

2.2.9.2 Transition State

TS

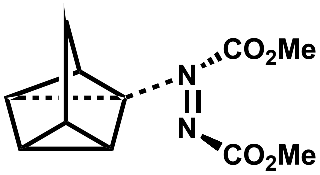

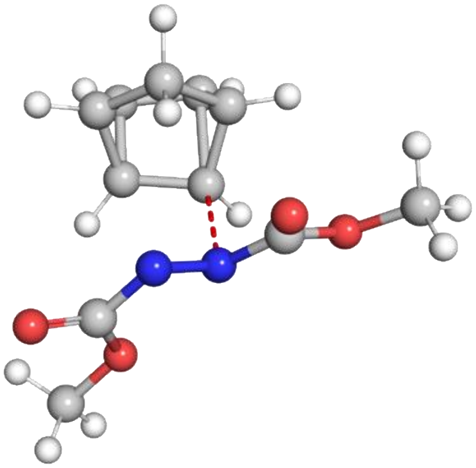

Imaginary frequency at -635.4 cm^{-1}

E	*H*	*G*
-837.603516	-837.602571	-837.662868

2.2.9.3 Product

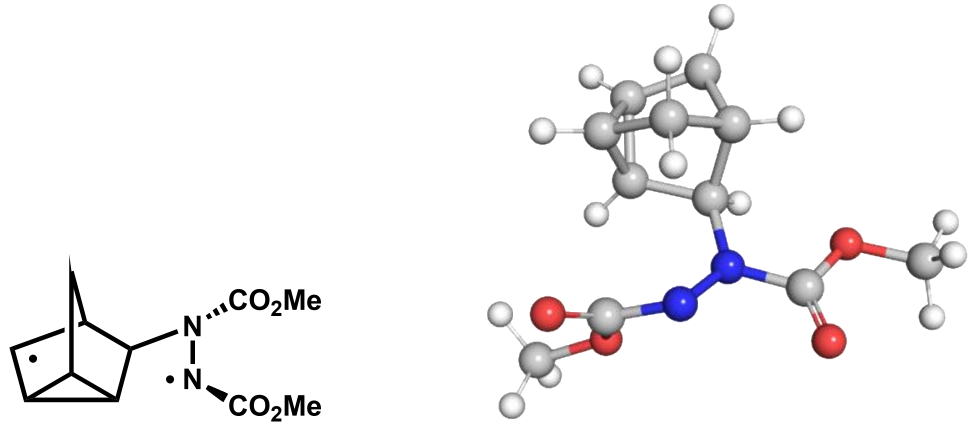

E	*H*	*G*
-837.658822	-837.657878	-837.720224

2.2.10 wB97X-D/ma-def2-TZVP with explicit H_2O within the SMD model for toluene

2.2.10.1 Reactant

Dimethylazodicarboxylate

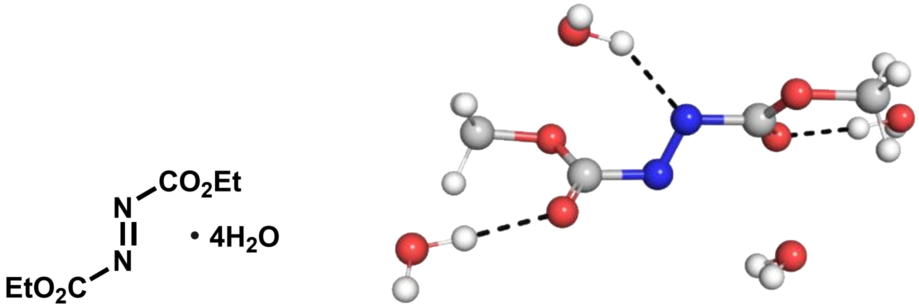

E	*H*	*G*
-872.012952	-872.012007	-872.086984

2.2.10.2 Transition State

TS$_w$

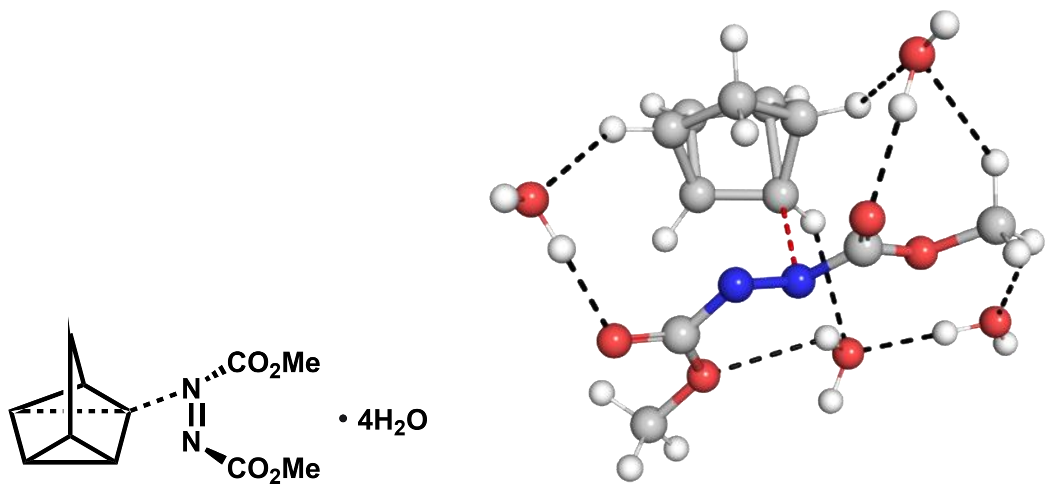

Imaginary frequency at -577.3 cm^{-1}

E	*H*	*G*
-1143.308784	-1143.307840	-1143.400745

2.3 Diels-Alder reaction

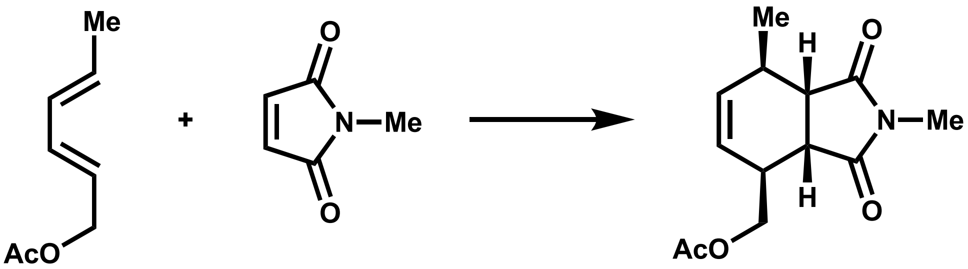

2.3.1 PM6-D3H4 calculation in toluene

2.3.1.1 Reactants

Diene

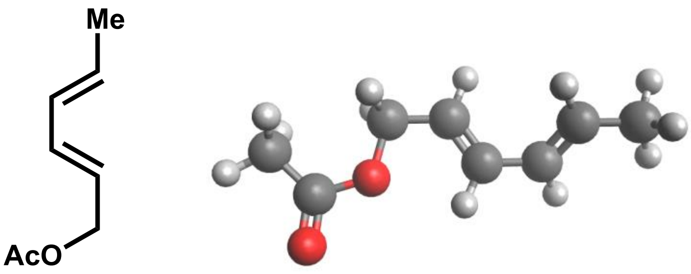

G (PM6-D3H4) kcal/mol
-99.56

Dienophile

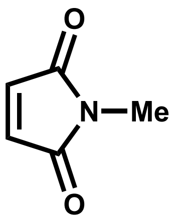

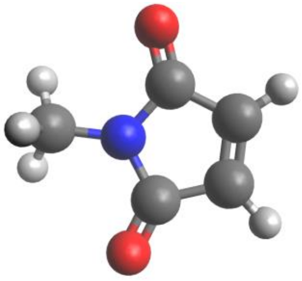

G (PM6-D3H4) kcal/mol
-78.12

2.3.1.2 Transition State

TS

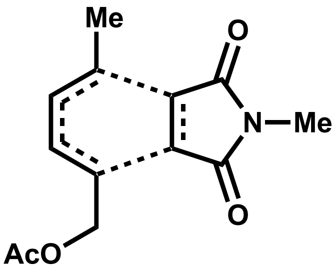

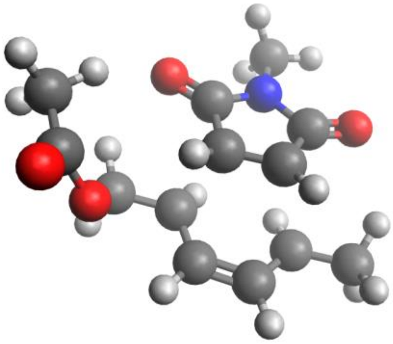

G (PM6-D3H4) kcal/mol
-145.91

2.3.1.3 Product

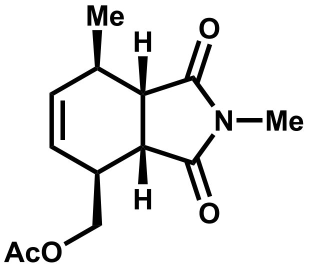

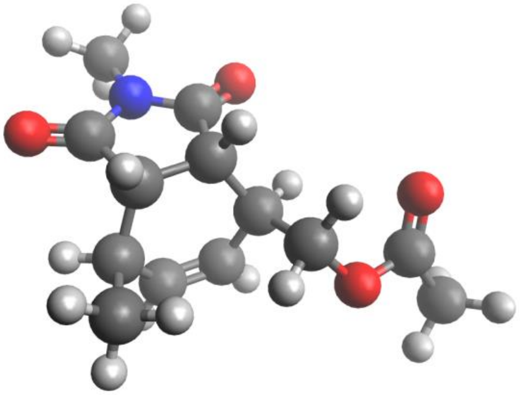

G (PM6-D3H4) kcal/mol
-217.65

2.3.2 PM6-D3H4 calculation in toluene with explicit water molecules

2.3.2.1 Transition State

TS

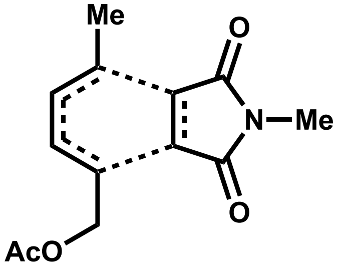

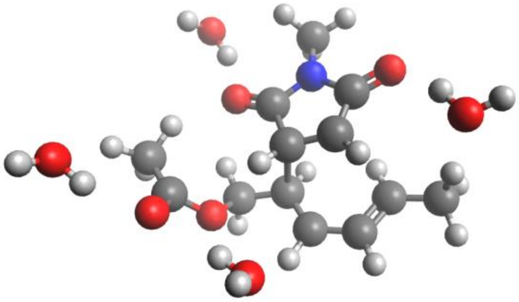

G (PM6-D3H4) kcal/mol
-405.72

2.3.2.2 Product

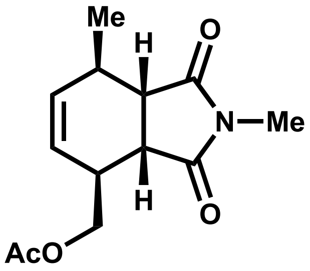

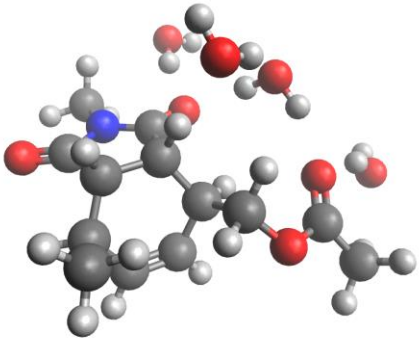

G (PM6-D3H4) kcal/mol
-477.12

2.3.3 B3LYP within the CPCM model for toluene

2.3.3.1 Reactants

Diene

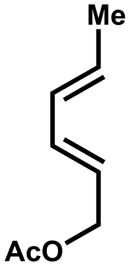

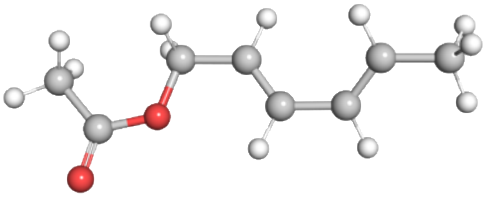

E (B3LYP)	*H (B3LYP)*	*G (B3LYP)*
-462.351371	-462.350427	-462.402227

Dienophile

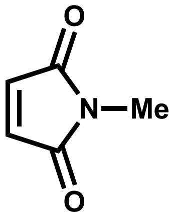

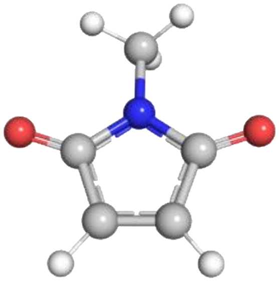

E (B3LYP)	*H (B3LYP)*	*G (B3LYP)*
-398.679975	-398.679030	-398.719552

2.3.3.2 Transition State

TS

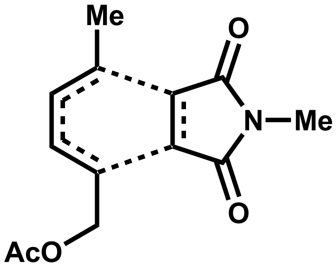

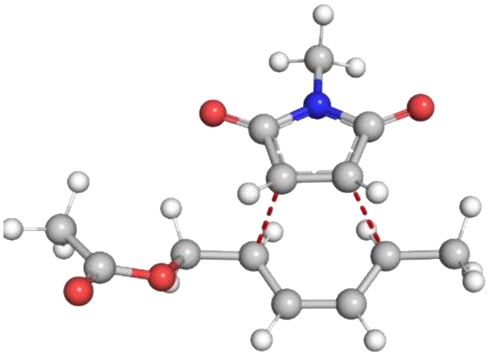

Imaginary frequency at -441.7 cm^{-1}

E (B3LYP)	*H (B3LYP)*	*G (B3LYP)*
-861.020332	-861.019388	-861.087815

2.3.3.3 Product

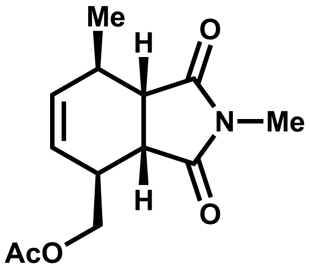

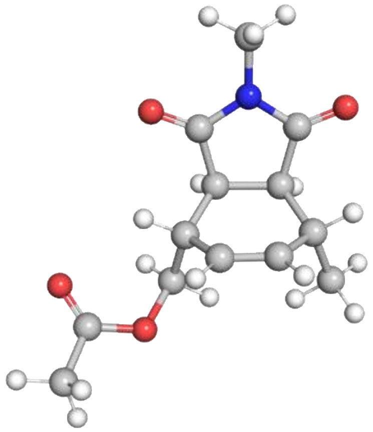

E (B3LYP)	*H (B3LYP)*	*G (B3LYP)*
-861.097765	-861.096820	-861.164496

2.3.4 B3LYP with explicit H_2O within the CPCM model for toluene

2.3.4.1 Reactant

Diene

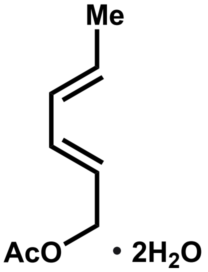

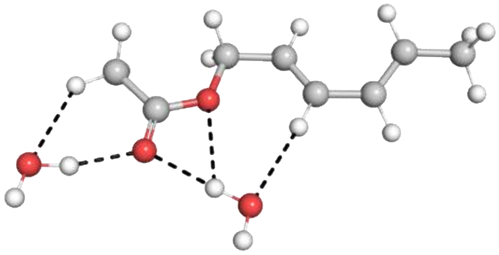

E (B3LYP)	*H (B3LYP)*	*G (B3LYP)*
-615.194805	-615.193861	-615.264479

Dienophile

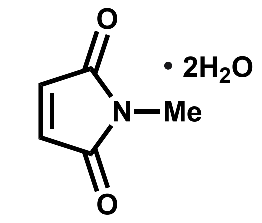

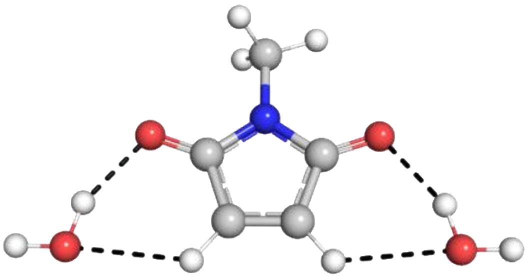

E (B3LYP)	*H (B3LYP)*	*G (B3LYP)*
-551.522221	-551.521277	-551.580837

2.3.4.2 Transition State

TS$_w$

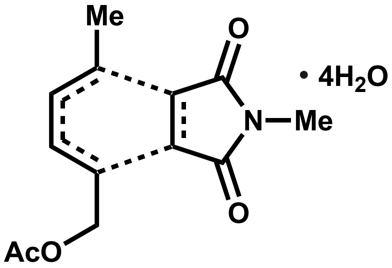

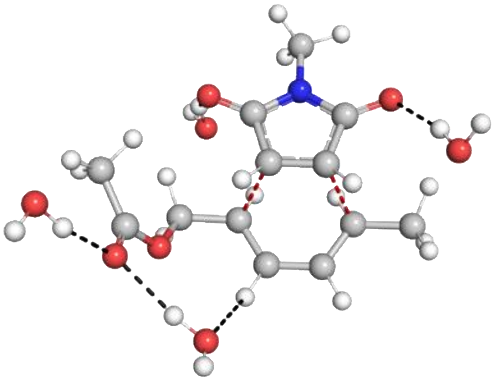

Imaginary frequency at -420.0 cm^{-1}

E (B3LYP)	*H (B3LYP)*	*G (B3LYP)*
-1166.708772	-1166.707828	-1166.810663

2.3.4.3 Product

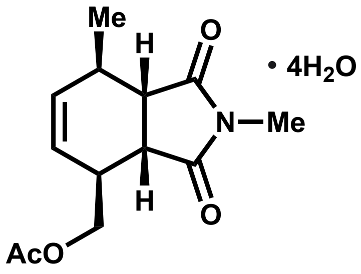

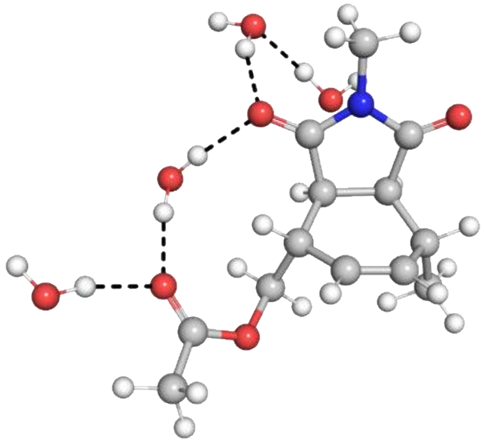

E (B3LYP)	*H (B3LYP)*	*G (B3LYP)*
-1166.794550	-1166.793606	-1166.890844

2.3.5 M06-2X within the CPCM model for toluene

2.3.5.1 Reactant

Diene

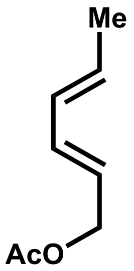

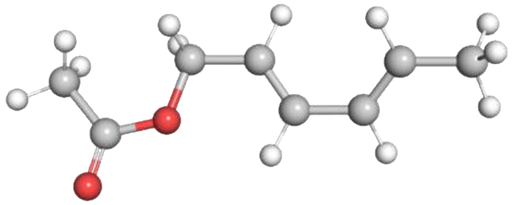

E (M06-2X)	*H (M06-2X)*	*G (M06-2X)*
-461.739619	-461.738675	-461.790581

Dienophile

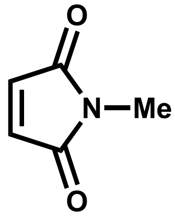

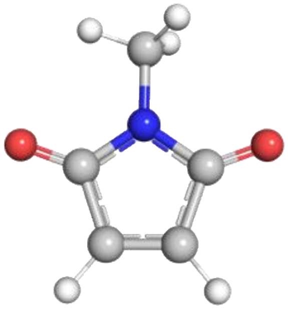

E (M06-2X)	*H (M06-2X)*	*G (M06-2X)*
-398.186540	-398.185596	-398.225248

2.3.5.2 Transition State

TS

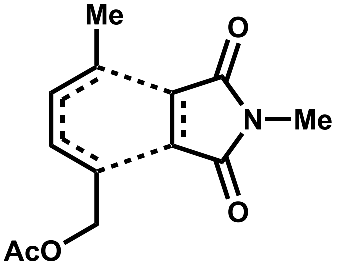

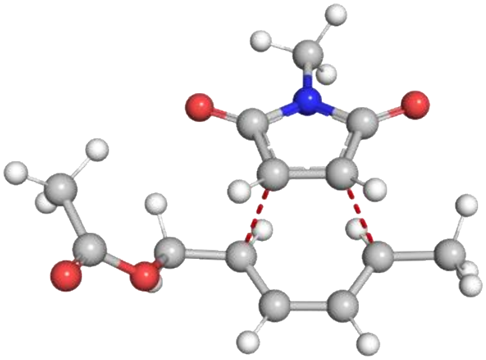

Imaginary frequency at -428.2 cm^{-1}

E (M06-2X)	*H (M06-2X)*	*G (M06-2X)*
-859.919006	-859.918062	-859.983711

2.3.5.3 Product

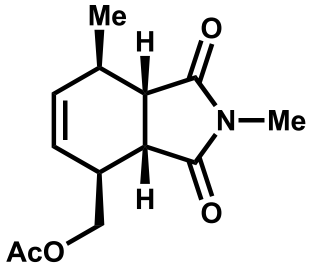

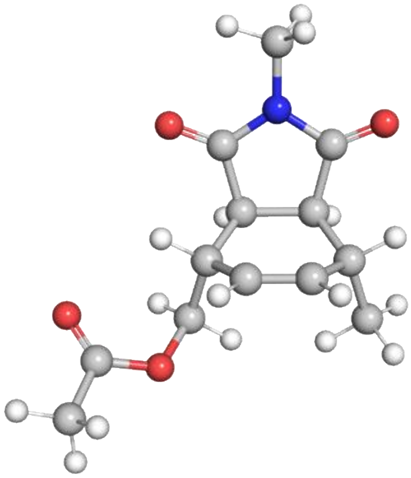

E (M06-2X)	*H (M06-2X)*	*G (M06-2X)*
-860.012483	-860.011539	-860.077540

2.3.6 M06-2X with explicit H_2O within the CPCM model for toluene

2.3.6.1 Reactant

Diene

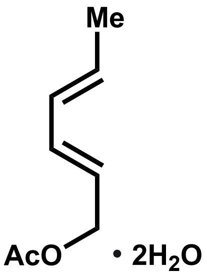

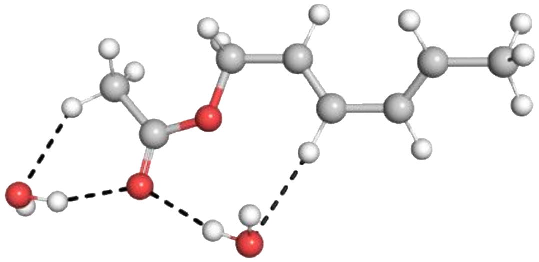

E (M06-2X)	*H (M06-2X)*	*G (M06-2X)*
-614.367485	-614.366540	-614.433248

Dienophile

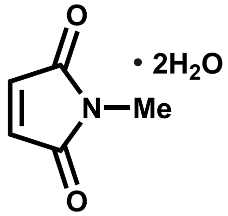

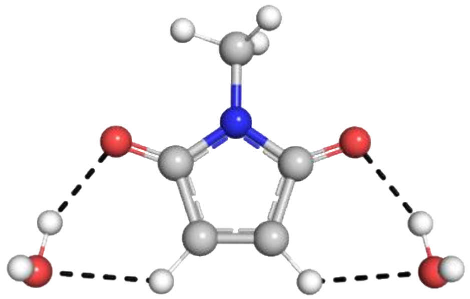

E (M06-2X)	*H (M06-2X)*	*G (M06-2X)*
-550.811719	-550.810775	-550.865473

2.3.6.2 Transition State

TS_w

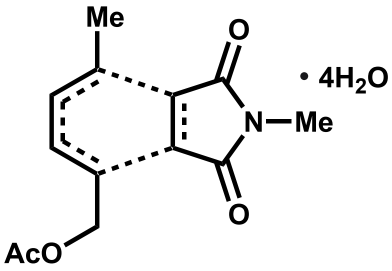

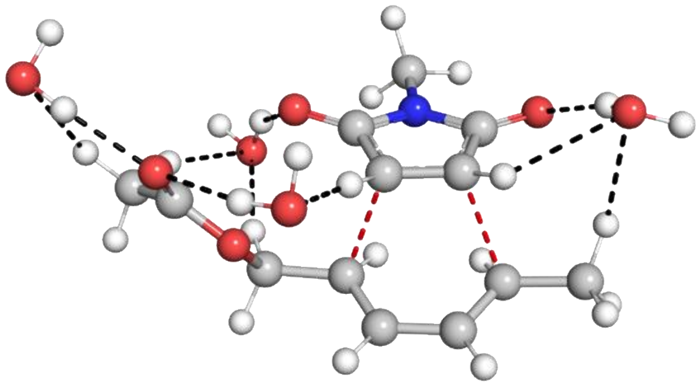

Imaginary frequency at -452.0 cm^{-1}

E (M06-2X)	*H (M06-2X)*	*G (M06-2X)*
-1165.179132	-1165.178188	-1165.270996

2.3.7 wB97X-D/def2-TZVP within the SMD model for toluene

2.3.7.1 Reactants

Diene

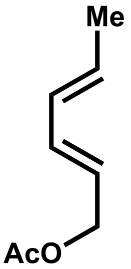

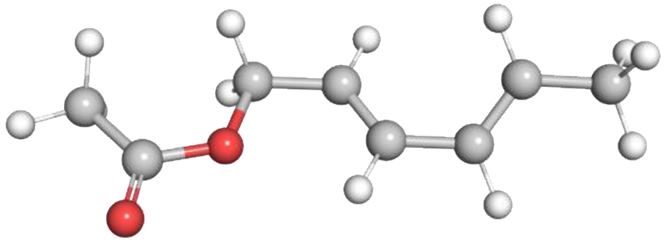

E (wB97X-D)	*H (wB97X-D)*	*G (wB97X-D)*
-462.332471	-462.331527	-462.382659

Dienophile

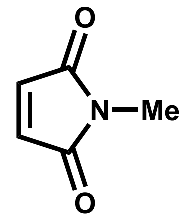

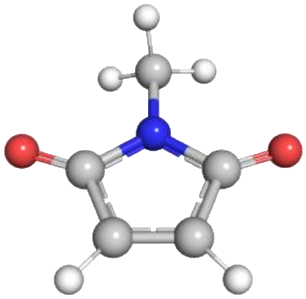

E (wB97X-D)	*H (wB97X-D)*	*G (wB97X-D)*
-398.676551	-398.675606	-398.716261

2.3.7.2 Transition State

TS

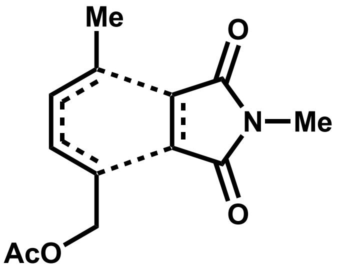

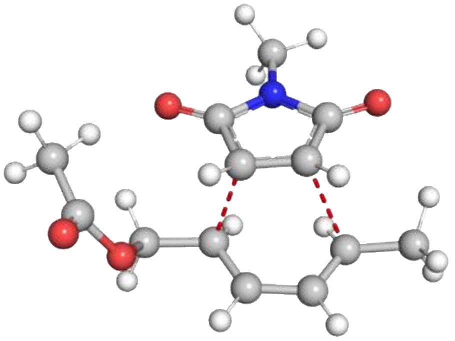

Imaginary frequency at -536.1 cm^{-1}

E (wB97X-D)	H (wB97X-D)	G (wB97X-D)
-860.987366	-860.986421	-861.053648

2.3.7.3 Product

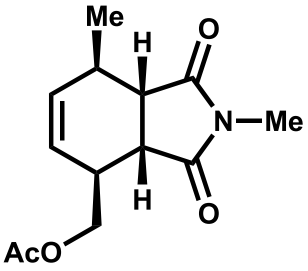

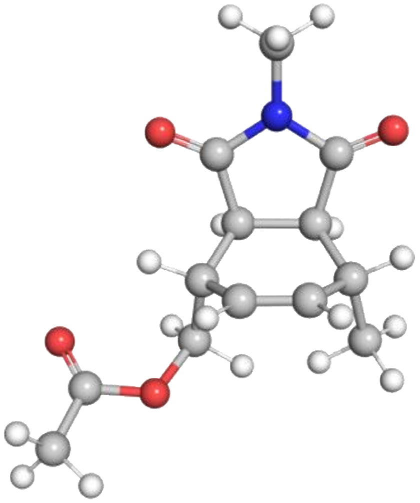

E (wB97X-D)	H (wB97X-D)	G (wB97X-D)
-861.082310	-861.081366	-861.145871

2.3.8 wB97X-D/def2-TZVP with explicit H_2O within SMD model for toluene

2.3.8.1 Reactant

Diene

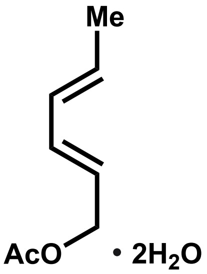

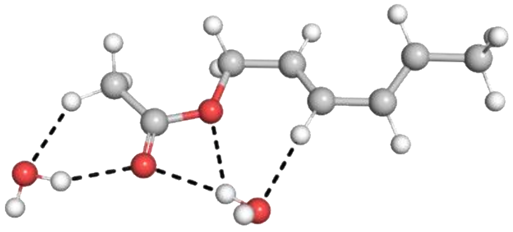

E (wB97X-D)	H (wB97X-D)	G (wB97X-D)

-615.178430	-615.177486	-615.246704

Dienophile

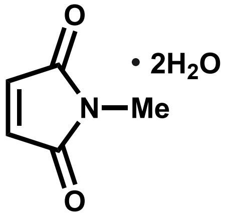

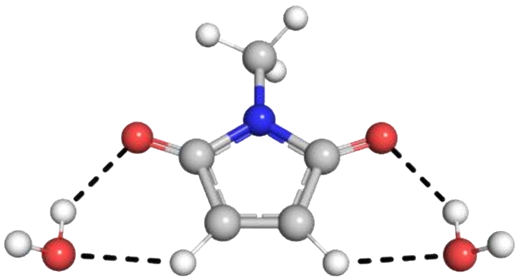

E (wB97X-D)	*H (wB97X-D)*	*G (wB97X-D)*
-551.520980	-551.520036	-551.578125

2.3.8.2 Transition State

TS_w

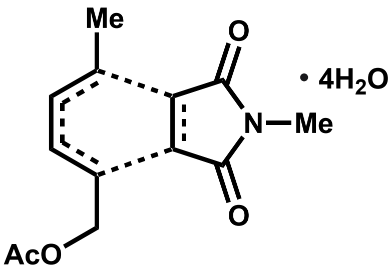

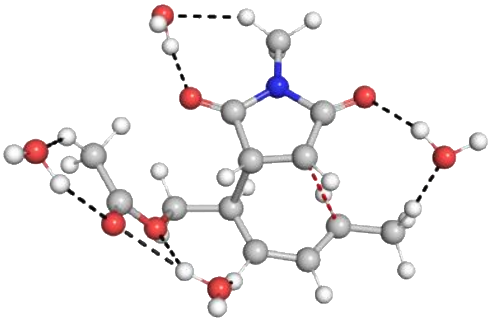

Imaginary frequency at -521.8 cm^{-1}

E (wB97X-D)	*H (wB97X-D)*	*G (wB97X-D)*
-1166.681217	-1166.680272	-1166.782946

2.3.9 wB97X-D/ma-def2-TZVP within the SMD model for toluene

2.3.9.1 Reactants

Diene

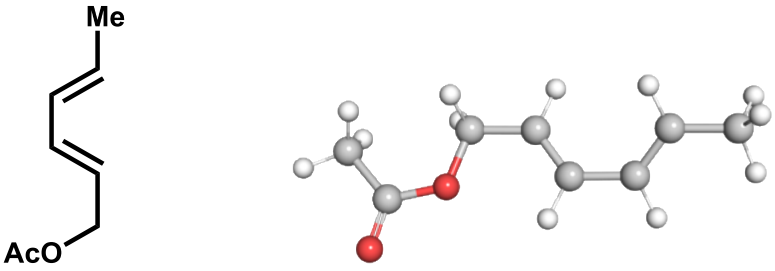

E (wB97X-D)	*H (wB97X-D)*	*G (wB97X-D)*
-462.333569	-462.332625	-462.383734

Dienophile

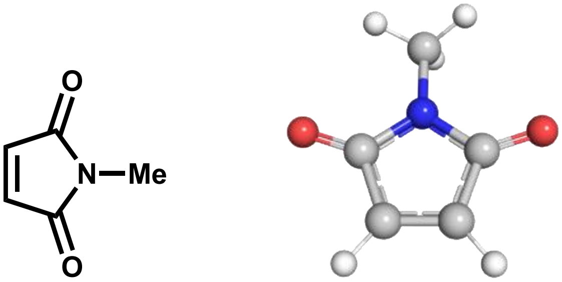

E (wB97X-D)	*H (wB97X-D)*	*G (wB97X-D)*
-398.677730	-398.676786	-398.717436

2.3.9.2 Transition State

TS

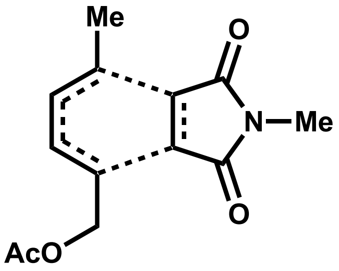

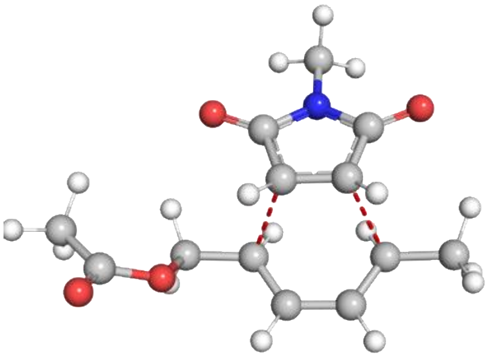

Imaginary frequency at -535.5 cm^{-1}

E (wB97X-D)	*H (wB97X-D)*	*G (wB97X-D)*
-860.989249	-860.988305	-861.055336

2.3.9.3 Product

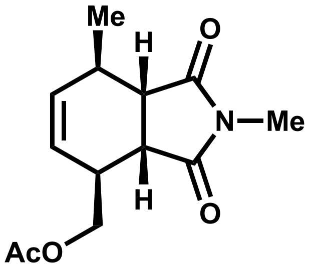

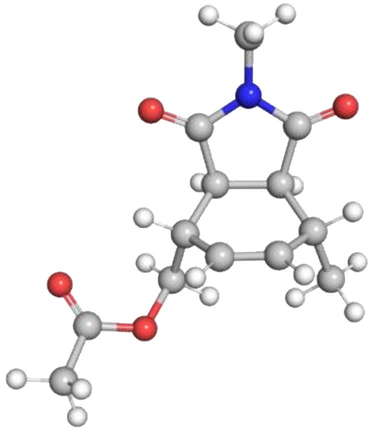

E (wB97X-D)	*H (wB97X-D)*	*G (wB97X-D)*
-861.084189	-861.083245	-861.147832

2.3.10 wB97X-D/ma-def2-TZVP with explicit H_2O within SMD model for toluene

2.3.10.1 Reactant

Diene

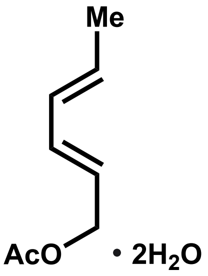

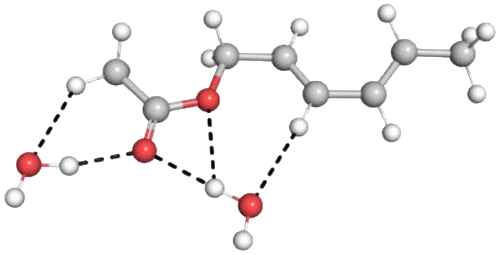

E (wB97X-D/ma)	H (wB97X-D/ma)	G (wB97X-D/ma)
-615.181490	-615.180546	-615.248818

Dienophile

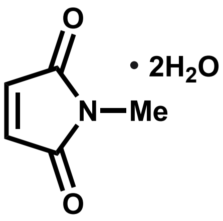

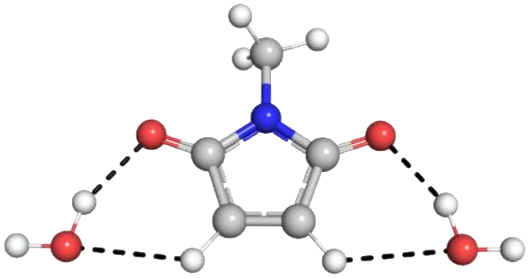

E (wB97X-D/ma)	H (wB97X-D/ma)	G (wB97X-D/ma)
-551.524385	-551.523441	-551.581556

2.3.10.2 Transition State

TS$_w$

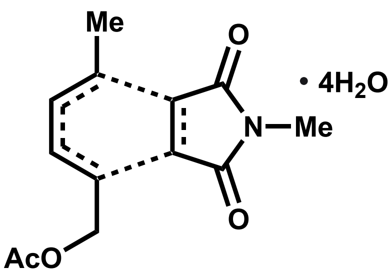

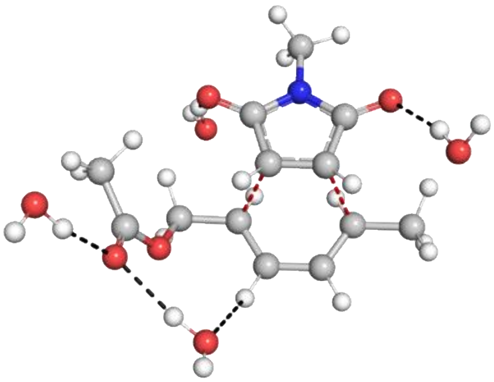

Imaginary frequency at -523.9 cm^{-1}

E (wB97X-D/ma)	*H (wB97X-D/ma)*	*G (wB97X-D/ma)*
-1166.687612	-1166.686668	-1166.787986

2.4 Claisen Rearrangement

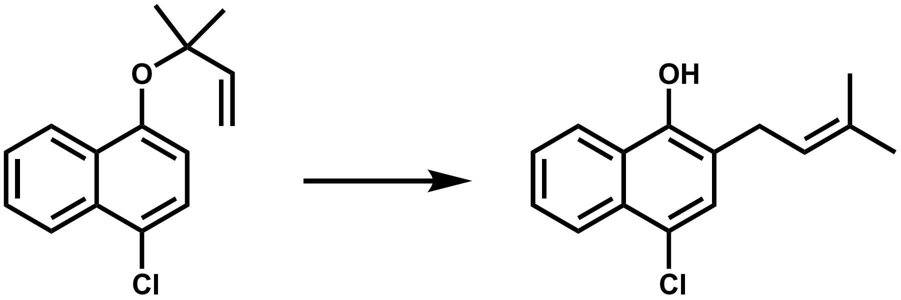

2.4.1 PM6-D3H4 calculations in toluene

2.4.1.1 Reactant

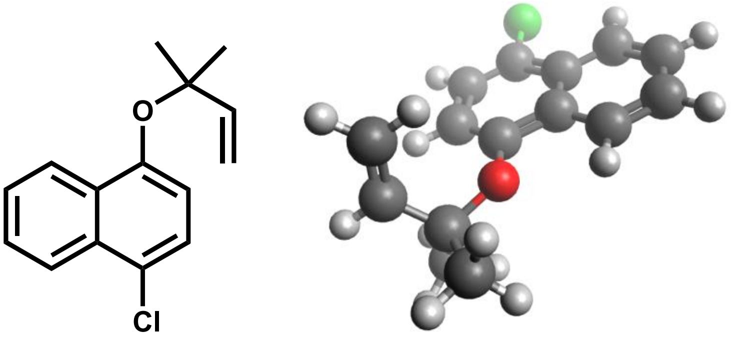

G (PM6-D3H4) kcal/mol
-43.30

2.4.1.2 Transition State

TS

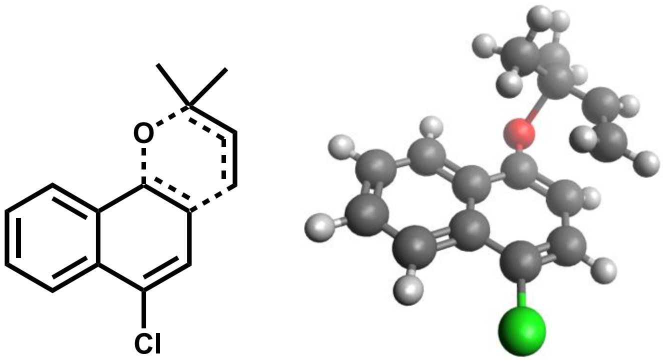

G (PM6-D3H4) kcal/mol
-13.89

2.4.1.3 Product

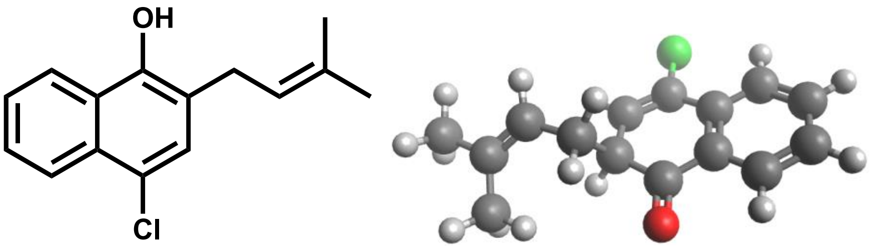

G (PM6-D3H4) kcal/mol
-53.98

2.4.2 PM6-D3H4 calculations in toluene with explicit water molecules

2.4.2.1 Reactant

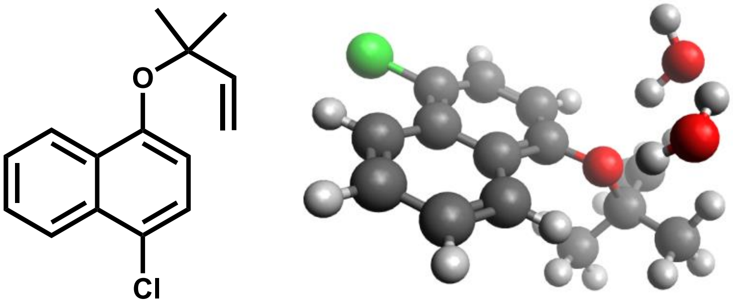

S68

G (PM6-D3H4) kcal/mol
-170.52

2.4.2.2 Transition State

TS

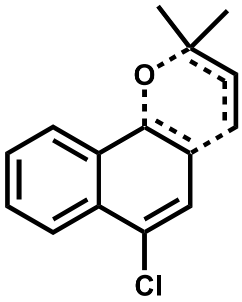

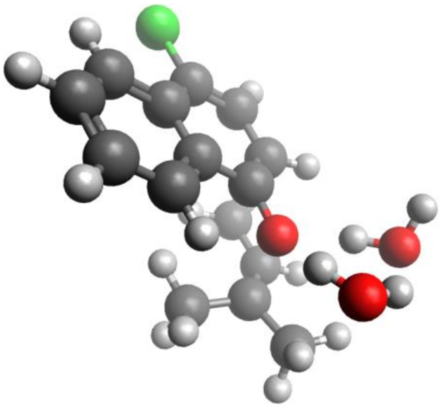

G (PM6-D3H4) kcal/mol
-144.78

2.4.3 B3LYP within the CPCM model for toluene

2.4.3.1 Reactant

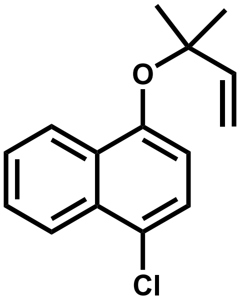

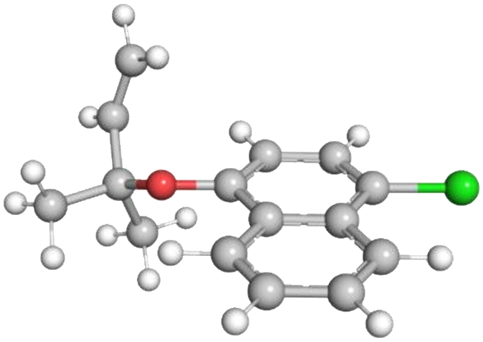

E (B3LYP)	*H (B3LYP)*	*G (B3LYP)*

-1115.838241	-1115.837297	-1115.896689

2.4.3.2 Transition State

TS

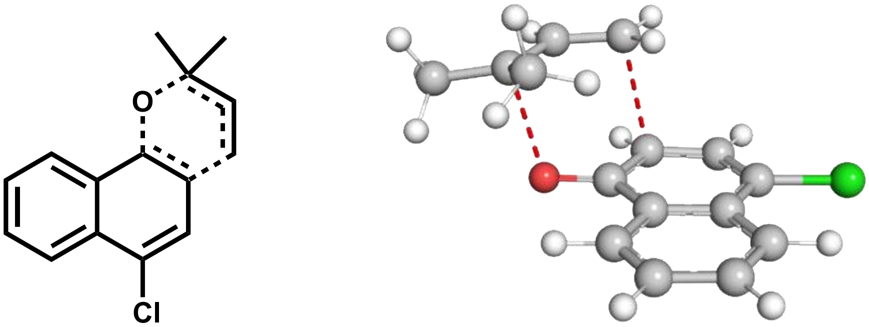

Imaginary frequency at -202.4 cm^{-1}

E (B3LYP)	*H (B3LYP)*	*G (B3LYP)*
-1115.808285	-1115.807341	-1115.866103

2.4.3.3 Product

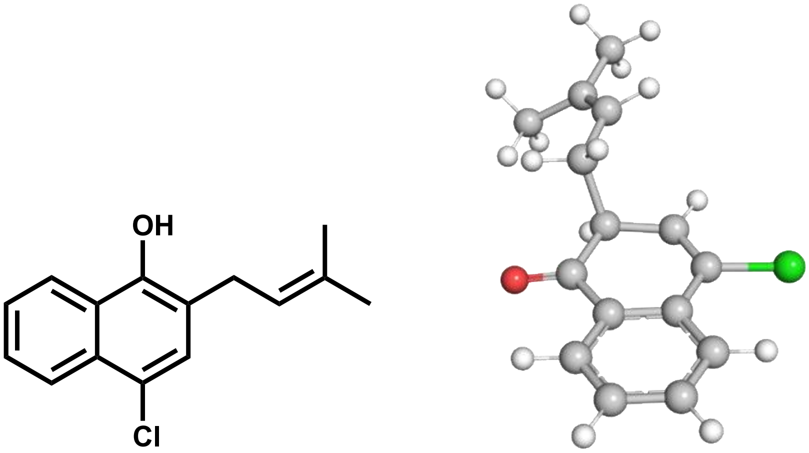

E (B3LYP)	*H (B3LYP)*	*G (B3LYP)*

-1115.840890	-1115.839946	-1115.902672

2.4.4 B3LYP with explicit H_2O within the CPCM model for toluene

2.4.4.1 Reactant

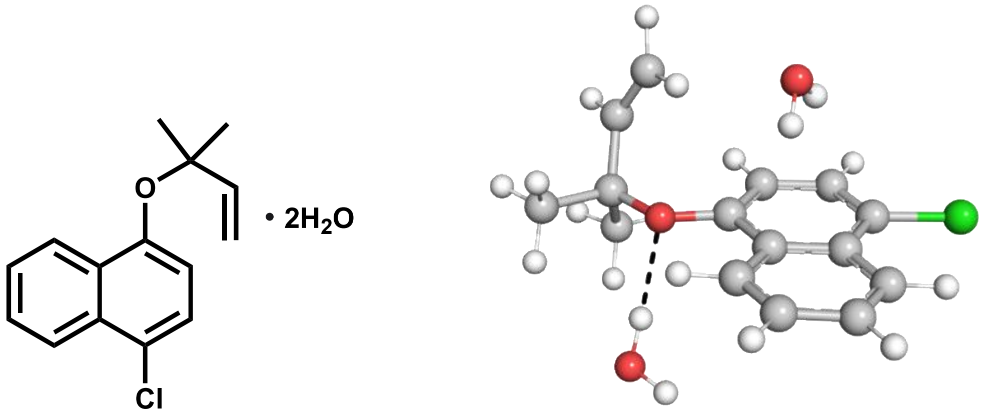

E (B3LYP)	*H (B3LYP)*	*G (B3LYP)*
-1268.677439	-1268.676494	-1268.752735

2.4.4.2 Transition State

TS_w

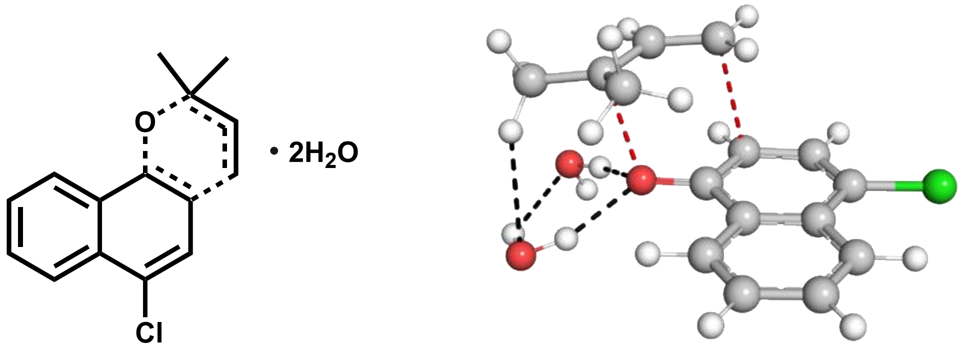

Imaginary frequency at -118.2 cm^{-1}

E (B3LYP)	*H (B3LYP)*	*G (B3LYP)*

-1268.660474	-1268.659530	-1268.732036

2.4.5 M06-2X within the CPCM model for toluene

2.4.5.1 Reactant

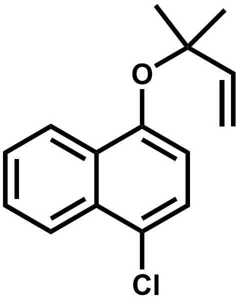

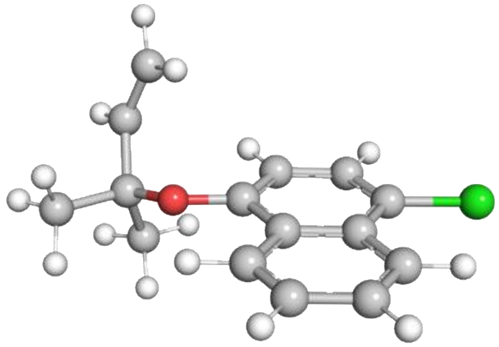

E (M06-2X)	*H (M06-2X)*	*G (M06-2X)*
-1114.844376	-1114.843431	-1114.902556

2.4.5.2 Transition State

TS

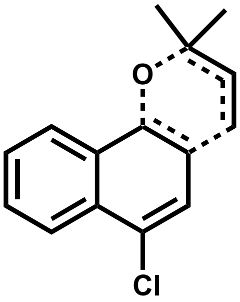

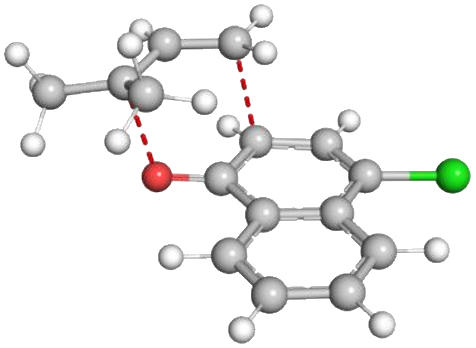

Imaginary frequency at -428.5 cm^{-1}

E (M06-2X)	*H (M06-2X)*	*G (M06-2X)*

-1114.797066	-1114.796122	-1114.853708

2.4.5.3 Product

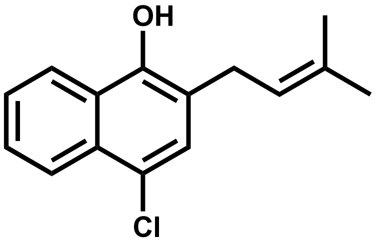

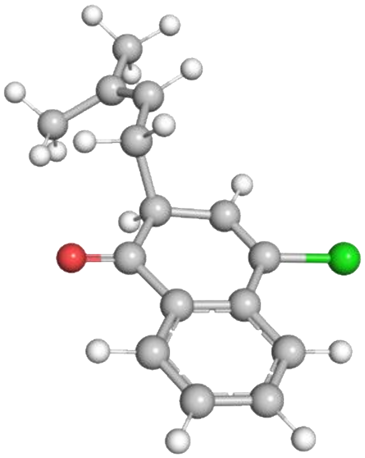

E (M06-2X)	*H (M06-2X)*	*G (M06-2X)*
-1114.842014	-1114.841070	-1114.903597

2.4.6 M06-2X with explicit H_2O within the CPCM model for toluene

2.4.6.1 Reactant

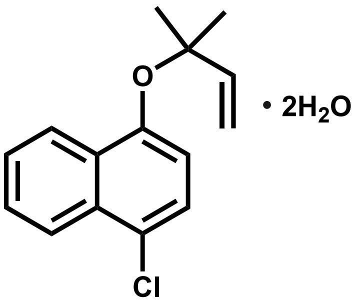

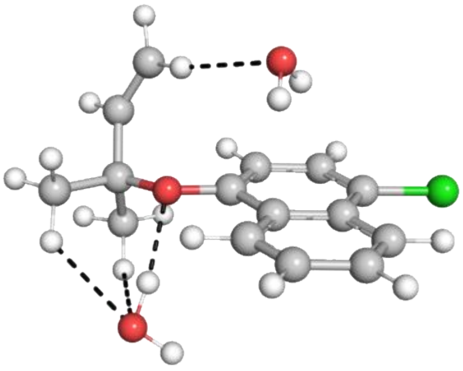

E (M06-2X)	*H (M06-2X)*	*G (M06-2X)*
-1267.469811	-1267.468867	-1267.542000

2.4.6.2 Transition State

TS$_w$

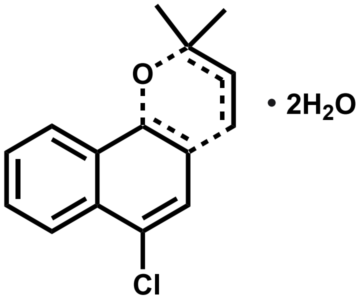

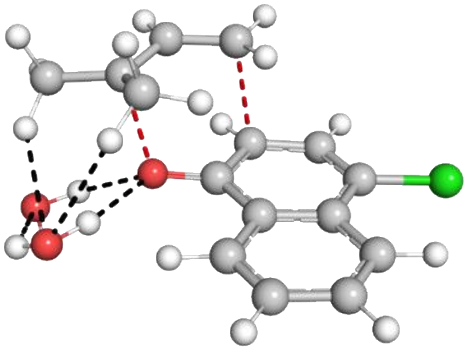

Imaginary frequency at -339.7 cm^{-1}

E (M06-2X)	*H (M06-2X)*	*G (M06-2X)*
-1267.433342	-1267.432398	-1267.502242

2.4.7 wB97X-D/def2-TZVP within the SMD model for toluene

2.4.7.1 Reactant

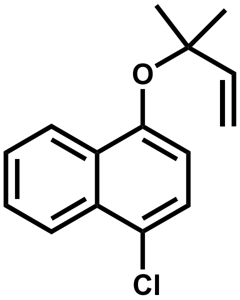

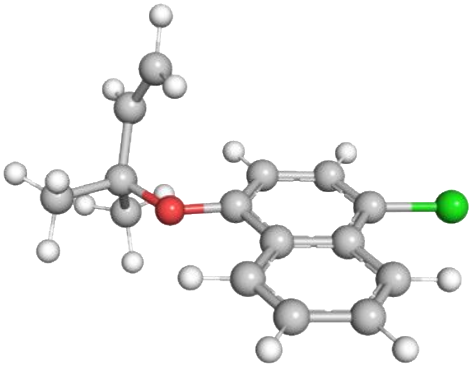

E (wB97X-D)	*H (wB97X-D)*	*G (wB97X-D)*
-1115.802219	-1115.801275	-1115.860061

2.4.7.2 Transition State

TS

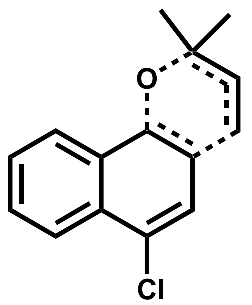

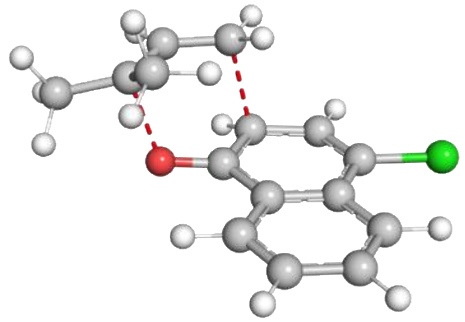

Imaginary frequency at -355.2 cm^{-1}

E (wB97X-D)	*H (wB97X-D)*	*G (wB97X-D)*
-1115.759546	-1115.758602	-1115.816142

2.4.7.3 Product

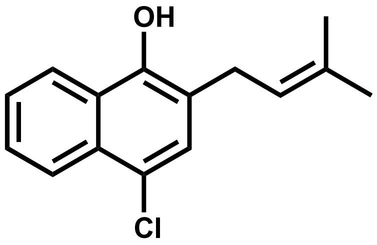

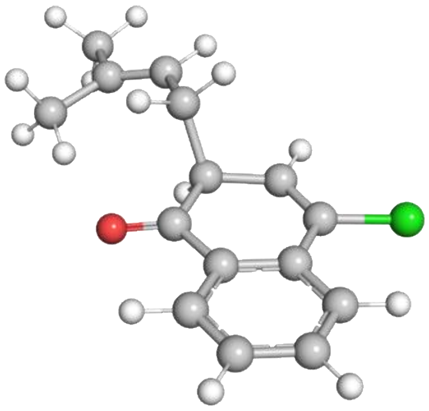

E (wB97X-D)	*H (wB97X-D)*	*G (wB97X-D)*
-1115.809715	-1115.808771	-1115.869348

2.4.8 wB97X-D/def2-TZVP with explicit H_2O within SMD model for toluene

2.4.8.1 Reactant

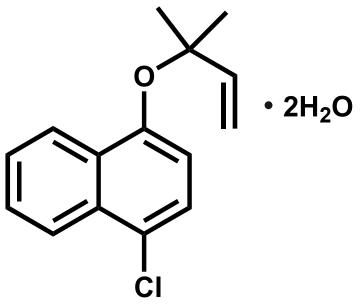

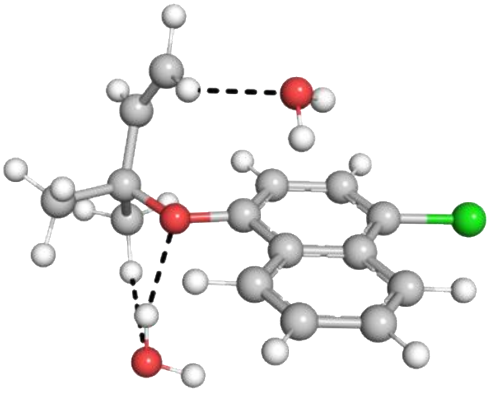

E (wB97X-D)	H (wB97X-D)	G (wB97X-D)
-1268.644815	-1268.643871	-1268.719201

2.4.8.2 Transition State

TS$_w$

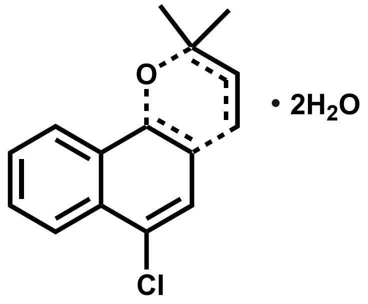

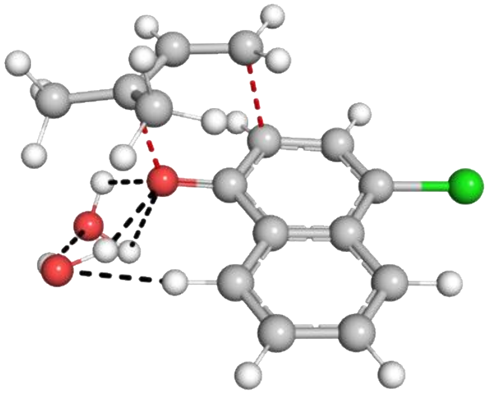

Imaginary frequency at -228.7 cm^{-1}

E (wB97X-D)	H (wB97X-D)	G (wB97X-D)
-1268.614639	-1268.613695	-1268.685390

2.4.9 wB97X-D/ma-def2-TZVP within the SMD model for toluene

2.4.9.1 Reactant

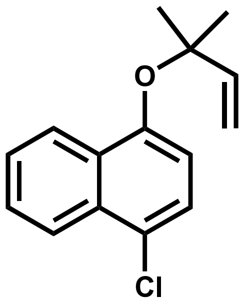

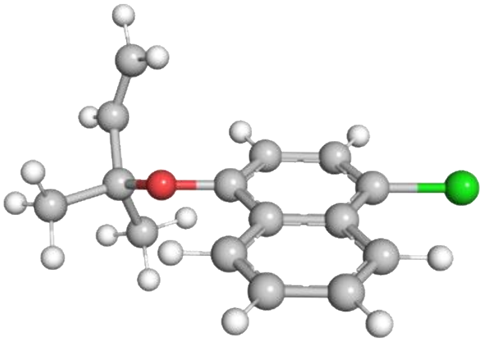

E (wB97X-D/ma)	*H (wB97X-D/ma)*	*G (wB97X-D/ma)*
-1115.803285	-1115.802341	-1115.861141

2.4.9.2 Transition State

TS

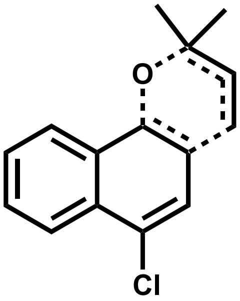

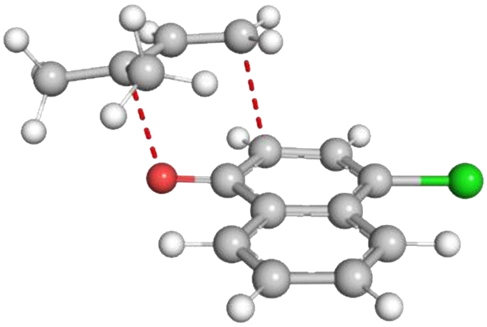

Imaginary frequency at -351.8 cm^{-1}

E (wB97X-D/ma)	*H (wB97X-D/ma)*	*G (wB97X-D/ma)*
-1115.760833	-1115.759889	-1115.817461

2.4.9.3 Product

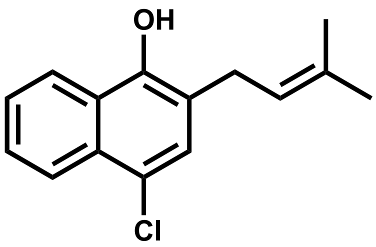

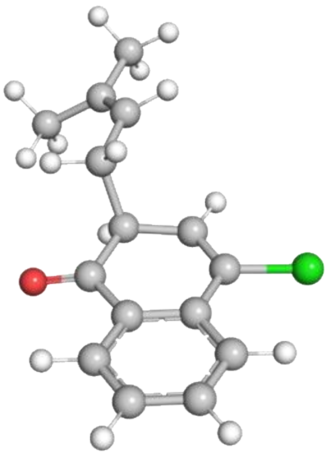

E (wB97X-D/ma)	*H (wB97X-D/ma)*	*G (wB97X-D/ma)*
-1115.810881	-1115.809937	-1115.870495

2.4.10 wB97X-D/ma-def2-TZVP with explicit H_2O within SMD model for toluene

2.4.10.1 Reactant

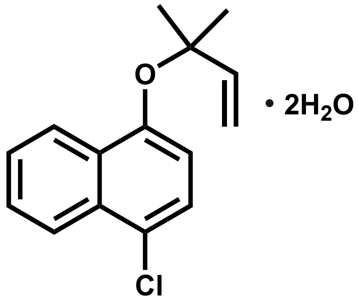

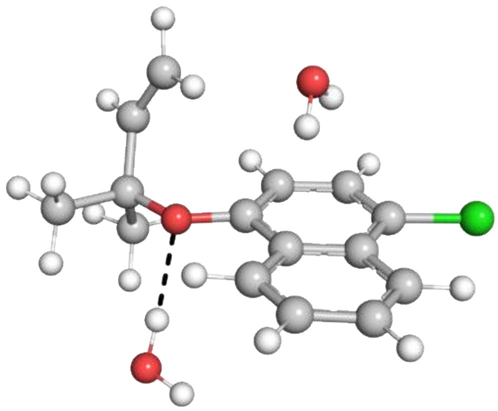

E (wB97X-D/ma)	*H (wB97X-D/ma)*	*G (wB97X-D/ma)*
-1268.647469	-1268.646525	-1268.722920

2.4.10.2 Transition State

TS_w

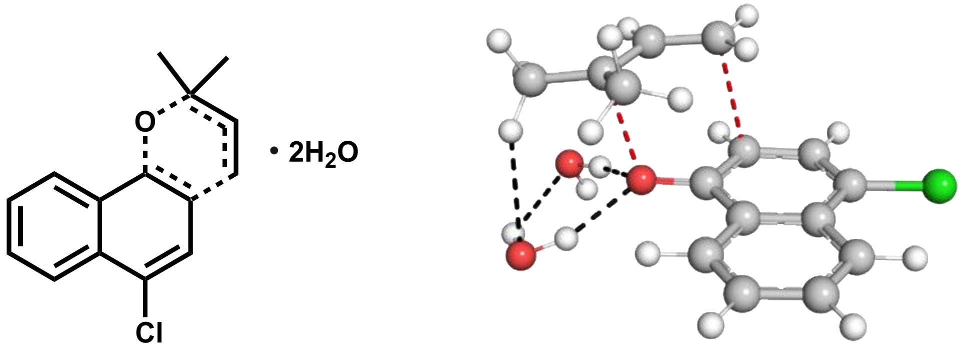

Imaginary frequency at -229.1 cm^{-1}

E (wB97X-D/ma)	*H (wB97X-D/ma)*	*G (wB97X-D/ma)*
-1268.617614	-1268.616670	-1268.689142

2.5 Summary of different molecular modelling techniques

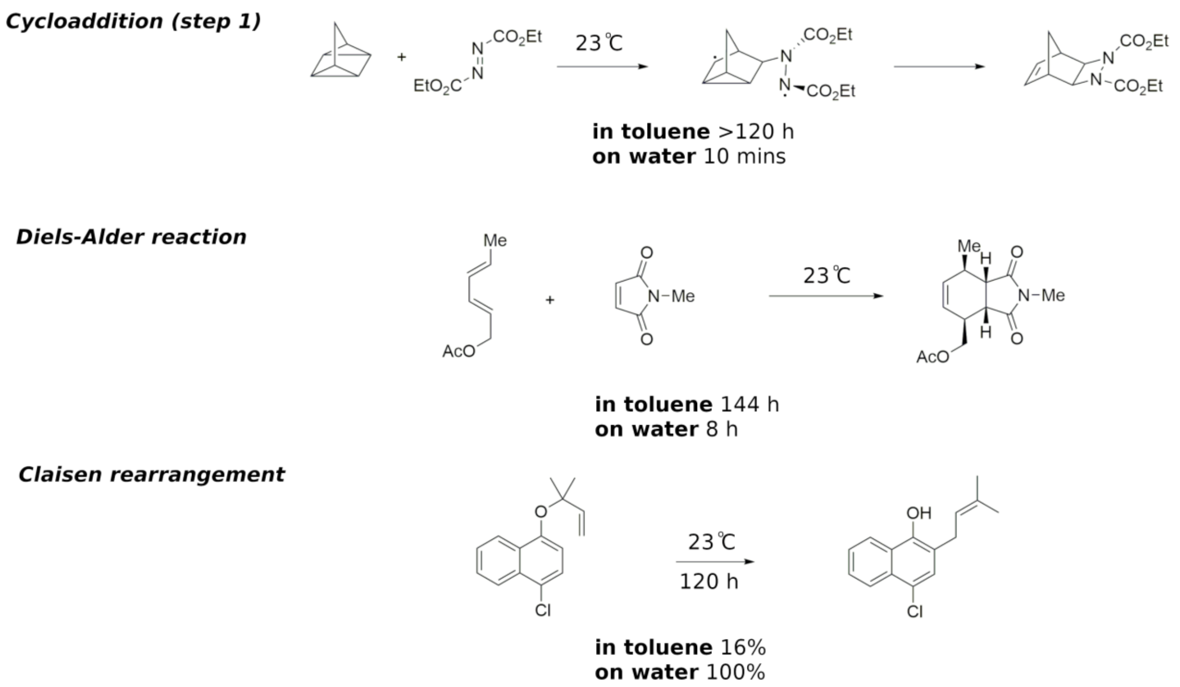

Figure S25. Experimental results on water acceleration effect of three reactions reported by Sharpless

We planned to compare three computational methods: PM6-D3H4, B3LYP/6-31+G(d,p), M06-2x/def2-SVP, wB97X-D/def2-TZVP, and wB97X-D/ma-def2-TZVP, against three well-known water-accelerated reactions with well-defined transition states at 298.15 K (Figure S25).[11,12] For the Diels-Alder reaction, a methyl group on the nitrogen atom was used instead of the ethyl group used by Sharpless to simplify conformational complexity.[12] The methods were selected for their speed (PM6-D3H4), historical precedent (B3LYP/6-31G+(d,p)),[11] and performance in main group systems where long-range interactions are important (M06-2x/def2-SVP, wB97X-D/def2-TZVP, and wB97X-D/ma-def2-TZVP). The ma-def2-TZVP was chosen to compare the effect of diffuse functions. Dispersion correction (D3) was included due to the importance of long-range stabilization of transition states by water molecules.[13] The aim of these calculations is to evaluate the reliability of each method in determining the decrease in activation energy conferred by water-stabilized transition states at 298.15 K. In each case, the activation energies are calculated in toluene, and toluene (PCM model) with explicit water molecules. The COSMO solvent model was used for PM6-D3H4 as implemented in MOPAC2016, CPCM for B3LYP and M06-2X and SMD for wB97X-D. The results are summarized in Table S11.

Experimental $\Delta\Delta G^{\ddagger}$ values were calculated using:

$$\Delta\Delta G^{\ddagger} = -RT \ln\left(\frac{k_{H_2O}}{k_{tol}}\right)$$

where $\frac{k_{H_2O}}{k_{tol}}$ is the experimentally observed increase in rate. However, these values must be taken as qualitative rather than quantitative since no actual kinetic data was collected and the calculation was based solely on estimated differences in reaction time and temperature. Experimental values were taken from Sharpless et al. 2005. The relative experimental acceleration values were taken as the difference from the reaction in toluene and the reaction with water. All values were measured at 23ºC. Cycloaddition is the difference in time to completion, toluene: >120h, water accelerated: 10 mins. Diels-Alder is the difference in time to completion, toluene: 144h, water accelerated: 8h. Claisen condensation is the difference in yield after 120h, toluene: 16%, water accelerated: 100%, which is not a good quantitative measure of rate.

Table S10. Relative experimental acceleration values

**Reaction**	$\boldsymbol{\frac{k_{H_2O}}{k_{tol}}}$
Cycloaddition	720
Diels-Alder reaction	18
Claisen rearrangement	6.25

Table S11. Comparison between different computation methods in three water-accelerated reactions against experimental data

No.	Method	ΔG≠ (kcal.mol^{-1}) Cycloaddition	Number of H_2O molecules	ΔG≠ (kcal.mol^{-1}) Diels Alder	Number of H_2O molecules	ΔG≠ (kcal.mol^{-1}) Claisen rearrangement	Number of H_2O molecules
1	PM6-D3H4 in toluene	42.7	0	31.8	0	29.4	0
2	PM6-D3H4 with H_2O molecules*	44.1	4	29.3	4	25.7	2
3	**PM6-D3H4 Water* stabilization of TS**	**1.4**		**2.5**		**3.7**	
4	B3LYP/6-31+G(d,p) in toluene	27.1	0	21.7	0	19.2	0
5	B3LYP/6-31+G(d,p) with H_2O molecules	16.5	4	21.3	4	13.0	2
6	**B3LYP/6-31+G(d,p) Water stabilization of TS**	**10.6**		**0.4**		**6.2**	
7	M06-2x/def2-SVP in toluene	32.0	0	20.2	0	30.7	0
8	M06-2x/def2-SVP with H_2O molecules	24.8	4	17.4	4	24.9	2
9	**M06-2x/def2-SVP Water stabilization of TS**	**7.2**		**2.8**		**5.8**	
10	wB97X-D/def2-TZVP in toluene	40.6	0	28.4	0	33.4	0
11	wB97X-D/def2-TZVP with H_2O molecules	35.8	4	26.3	4	21.2	2
12	**wB97X-D/def2-TZVP Water stabilization of TS**	**4.8**		**2.1**		**12.2**	
13	wB97X-D /ma-def2-TZVP in toluene	40.6	0	28.8	0	27.4	0
14	wB97X-D/ma-def2-TZVP with H_2O molecules	35.4	4	26.6	4	21.2	2
15	**wB97X-D/ma-def2-TZVP Water stabilization of TS**	**5.2**		**2.2**		**6.2**	
16	Experimentally estimated	**3.9**		**1.7**		**1.1**	

*PM6-D3H4 calculations in MOPAC often fail to optimize correctly when water molecules are included, leaving extraneous negative frequencies.

Table S12. Comparison of single core CPU time for optimization of transition states using M06-2x-D3/def2-SVP and wB97X-D/ma-def2-TZVP.

**Transition state**	**CPU time**	
	**M06-2x-D3/def2-SVP**	**wB97X-D/ma-def2-TZVP**
Cycloaddition	33m	57d 1h 12m
Diels-Alder reaction	14h 31m	29d 20h 30m
Claisen rearrangement	2h 50m	20d 6h 31m

3 DFT calculations of Henry reaction

All calculations were carried out with Gaussian 09 package, using M06-2x/def2-SVP functionals and basis set. Where appropriate, PCM solvent model was applied using nitromethane, or ethanol as solvent, with explicit water molecules.

3.1 Calculations in nitromethane

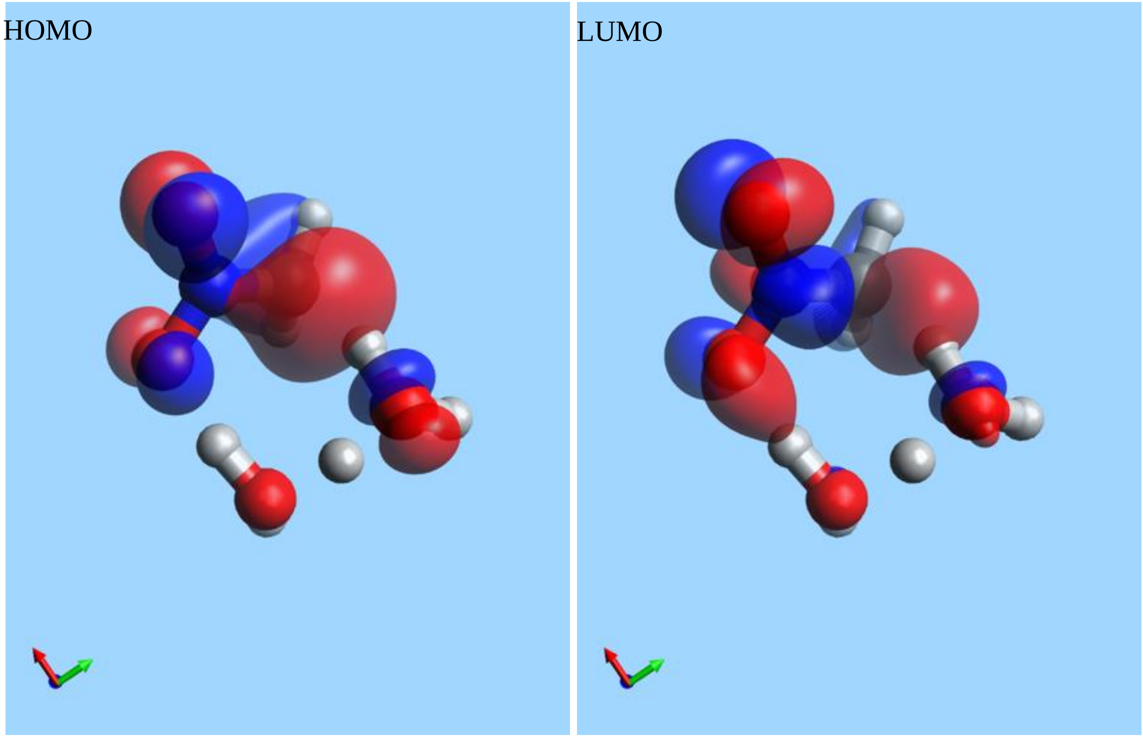

Figure S26. HOMO and LUMO of **TS1**$_{w}$

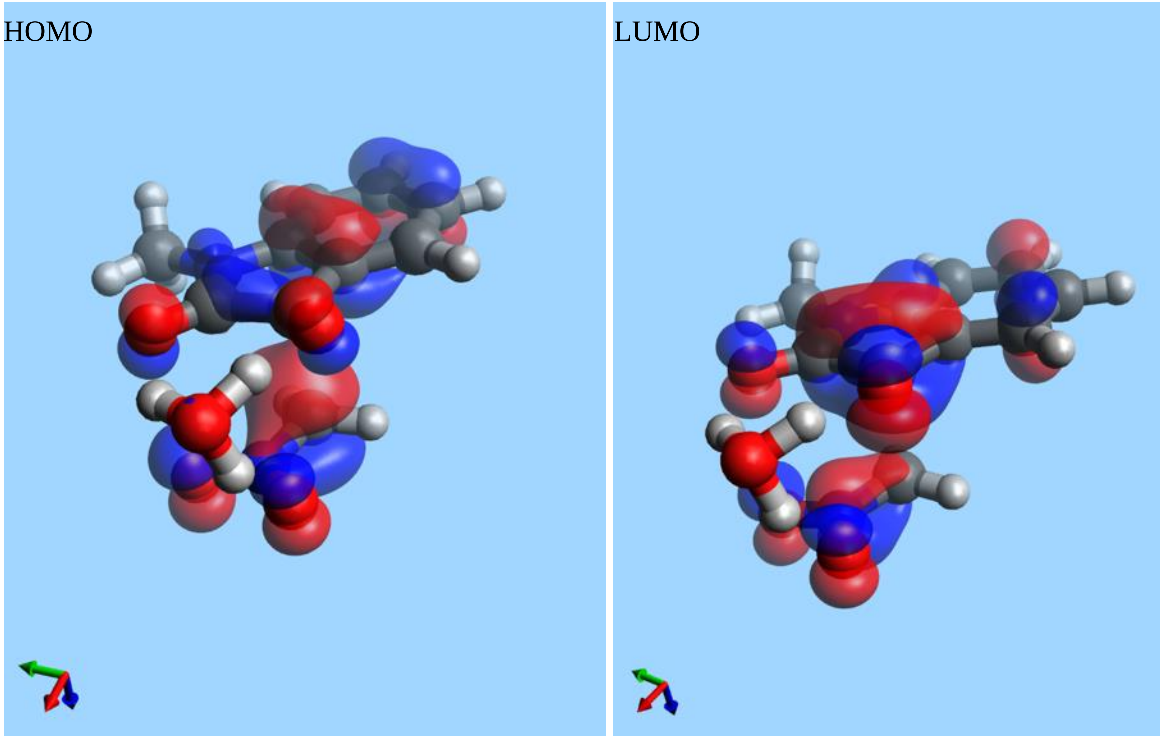

Figure S27. HOMO and LUMO of $\mathbf{TS2_w}$

3.1.1 Reactant

Nitromethane

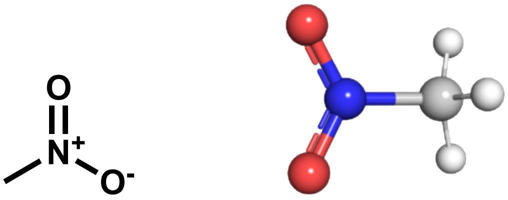

E (M06-2X)	*H (M06-2X)*	*G (M06-2X)*
-244.666741	-244.665797	-244.698803

Enolized nitromethane

3

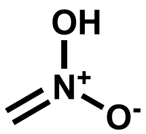

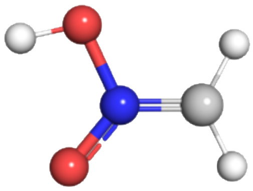

E (M06-2X)	H (M06-2X)	G (M06-2X)
-244.643725	-244.642780	-244.673726

Water

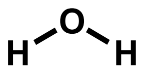

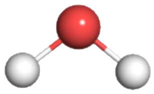

E (M06-2X)	H (M06-2X)	G (M06-2X)
-76.305700	-76.304755	-76.326193

N-methylisatin

1

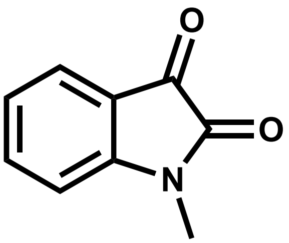

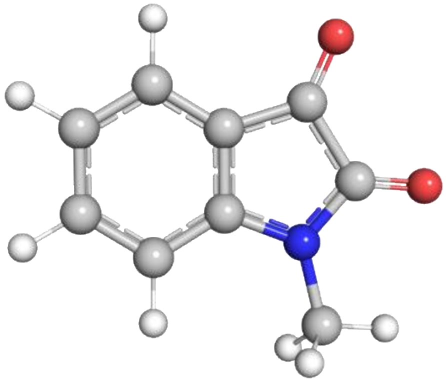

E (M06-2X)	H (M06-2X)	G (M06-2X)
-551.604521	-551.603577	-551.648701

3.1.2 Transition State

TS1$_w$

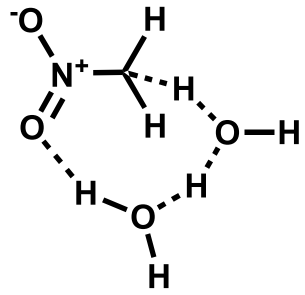

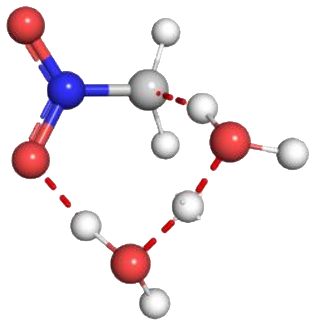

E (M06-2X)	*H (M06-2X)*	*G (M06-2X)*
-397.262993	-397.262049	-397.302275

TS2$_w$

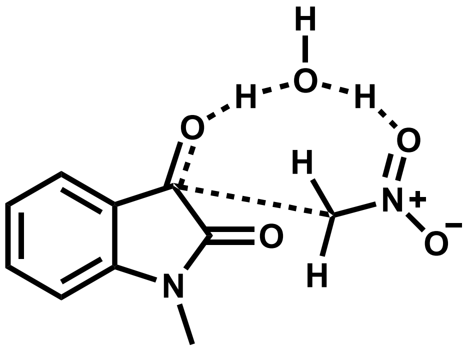

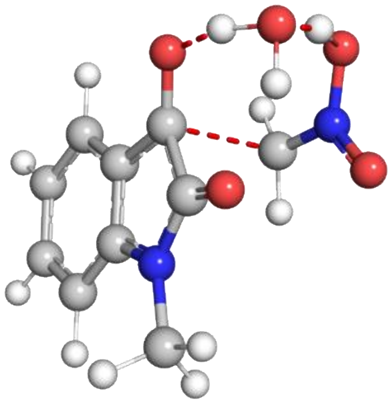

E (M06-2X)	*H (M06-2X)*	*G (M06-2X)*
-872.568468	-872.567523	-872.625342

3.1.3 Product

2

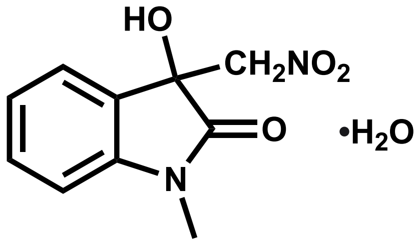

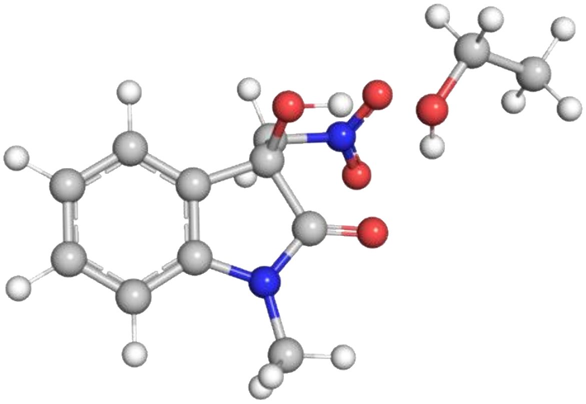

E (M06-2X)	H (M06-2X)	G (M06-2X)
-872.616490	-872.615546	-872.675158

3.2 Calculation in ethanol

3.2.1 Reactants

Nitromethane

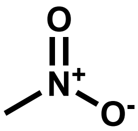

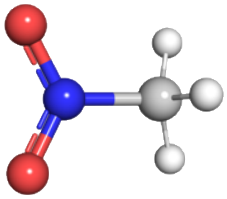

E (M06-2X)	H (M06-2X)	G (M06-2X)
-244.666608	-244.665664	-244.699306

Enolized nitromethane

3

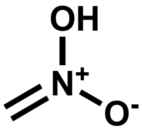

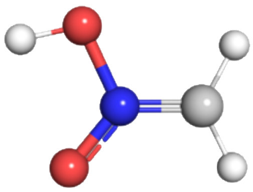

E (M06-2X)	*H (M06-2X)*	*G (M06-2X)*
-244.643617	-244.642673	-244.673617

Ethanol

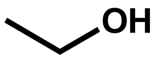

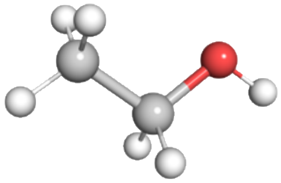

E (M06-2X)	*H (M06-2X)*	*G (M06-2X)*
-154.759549	-154.758605	-154.789257

N-methylisatin

1

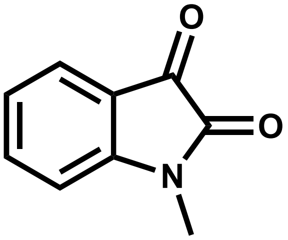

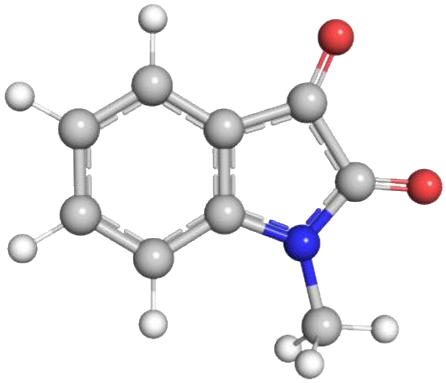

E (M06-2X)	*H (M06-2X)*	*G (M06-2X)*
-551.604339	-551.603395	-551.648521

3.2.2 Transition State

$TS1_{EtOH}$

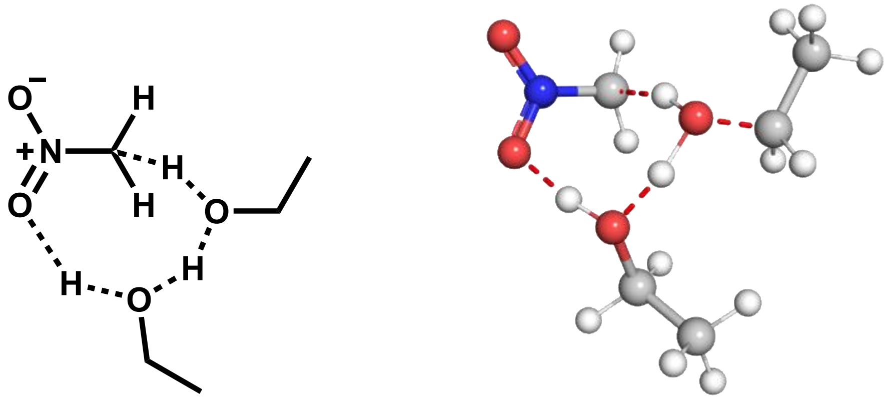

E (M06-2X)	*H (M06-2X)*	*G (M06-2X)*
-554.172347	-554.171403	-554.227619

TS2$_{\mathbf{EtOH}}$

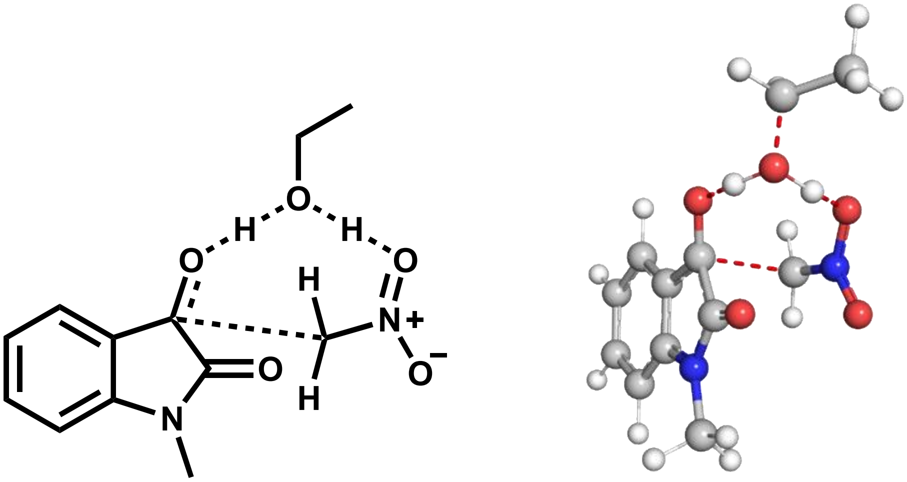

E (M06-2X)	*H (M06-2X)*	*G (M06-2X)*
-951.017656	-951.016712	-951.083779

3.2.3 Product

2

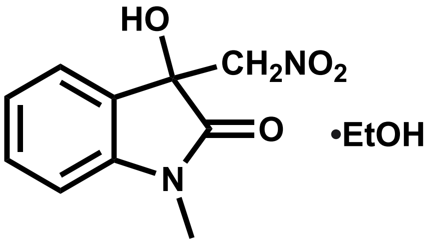

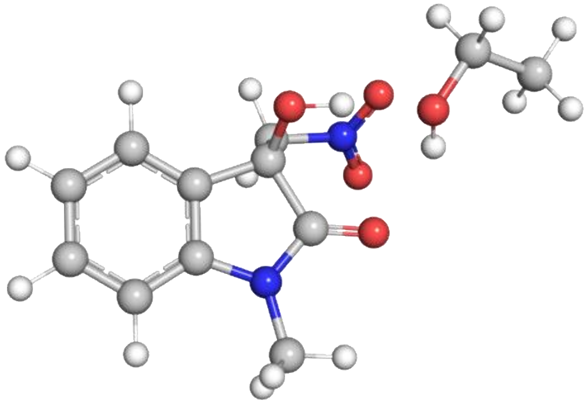

E (M06-2X)	*H (M06-2X)*	*G (M06-2X)*
-951.069126	-951.068182	-951.137024

3.3 Calculation in nitromethane at 90 ºC

3.3.1 Reactants

Nitromethane

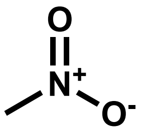

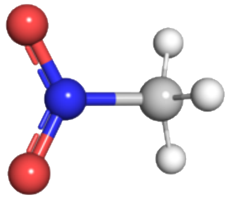

E (M06-2X)	*H (M06-2X)*	*G (M06-2X)*
-244.666561	-244.665411	-244.703611

Enolized nitromethane

3

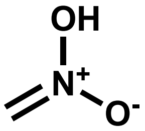

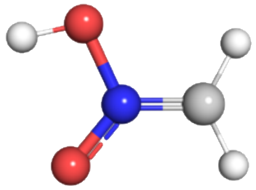

E (M06-2X)	H (M06-2X)	G (M06-2X)
-244.642234	-244.641084	-244.680641

Water

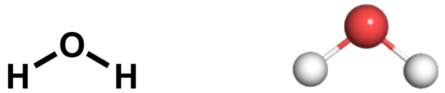

E (M06-2X)	H (M06-2X)	G (M06-2X)
-76.305073	-76.303923	-76.330951

N-methylisatin

1

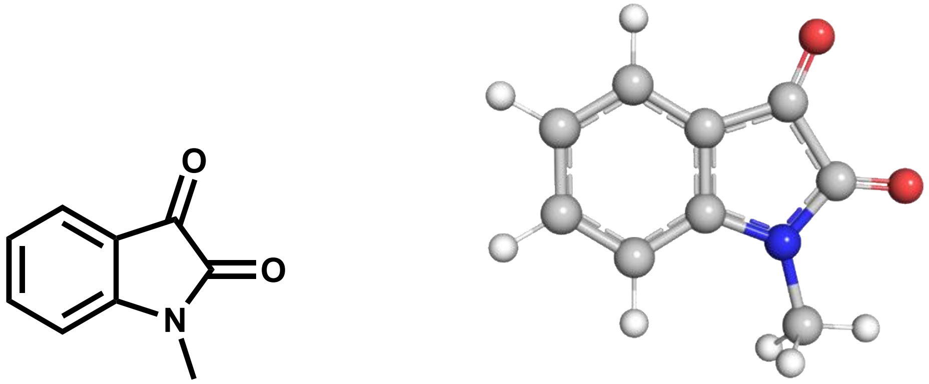

E (M06-2X)	H (M06-2X)	G (M06-2X)
-551.600408	-551.599258	-551.658965

3.3.2 Transition State

TS1$_w$

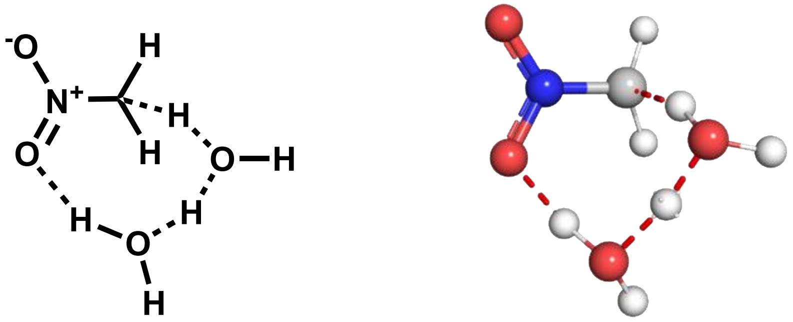

E (M06-2X)	*H (M06-2X)*	*G (M06-2X)*
-397.260075	-397.258925	-397.311355

TS2$_w$

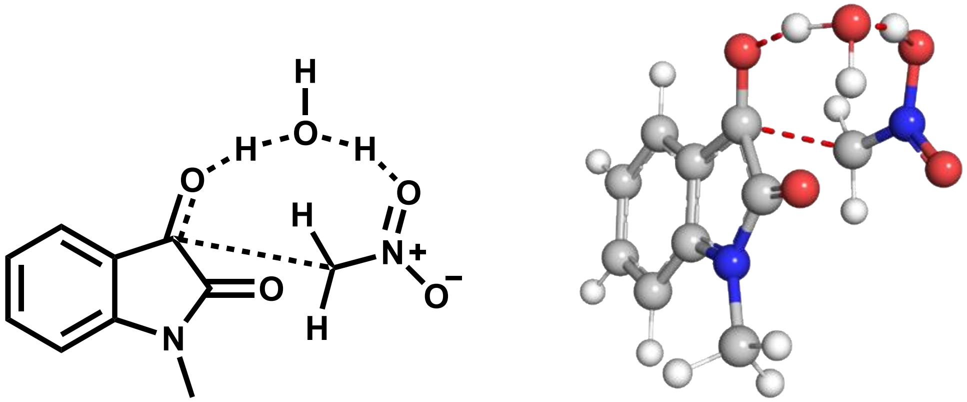

E (M06-2X)	*H (M06-2X)*	*G (M06-2X)*
-872.561899	-872.560749	-872.638617

3.3.3 Product

2

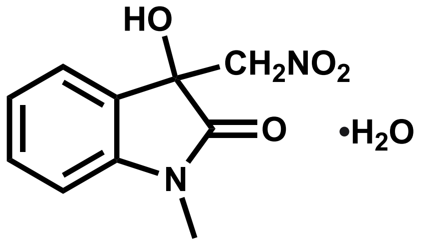

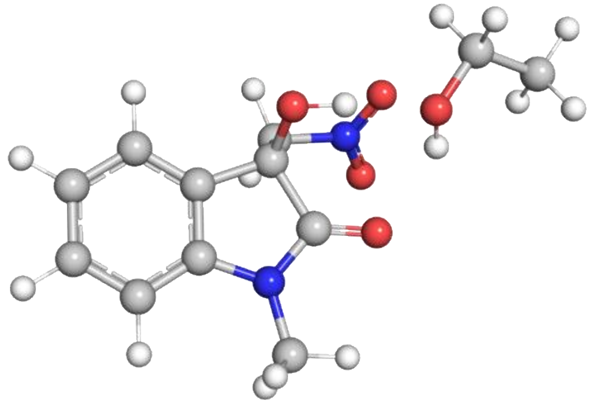

E (M06-2X)	H (M06-2X)	G (M06-2X)
-872.609699	-872.608549	-872.688846

3.4 Calculation in nitromethane with Na_2SO_4

3.4.1 Reactants

HSO_4^-

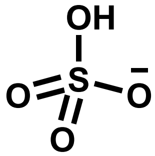

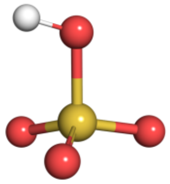

E (M06-2X)	H (M06-2X)	G (M06-2X)
-699.263503	-699.262558	-699.296499

3.4.2 Transition State

$TS1_{HSO4}$

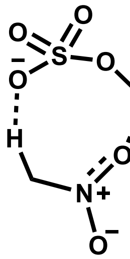 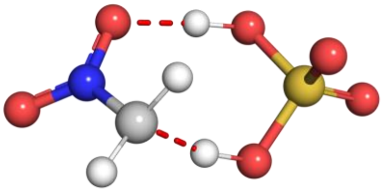

E (M06-2X)	*H (M06-2X)*	*G (M06-2X)*
-943.917592	-943.916648	-943.961111

$TS2_{HSO4}$

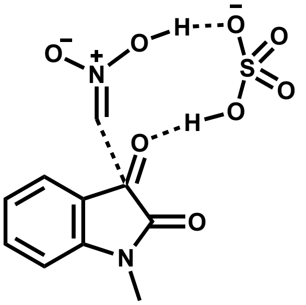 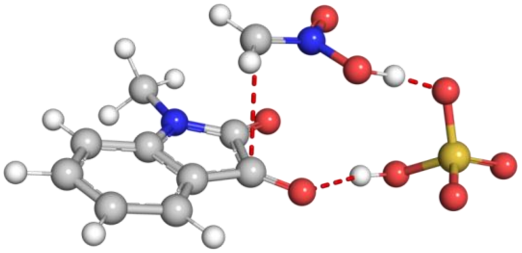

E (M06-2X)	*H (M06-2X)*	*G (M06-2X)*
-1495.549267	-1495.548322	-1495.619390

3.4.3 Product

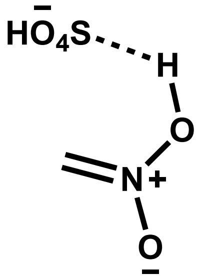 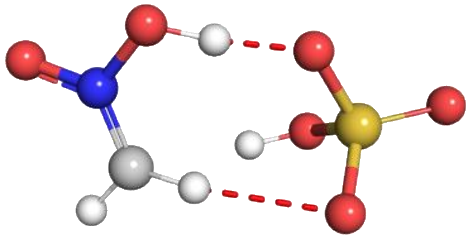

E (M06-2X)	*H (M06-2X)*	*G (M06-2X)*

-943.928072	-943.927128	-943.972169

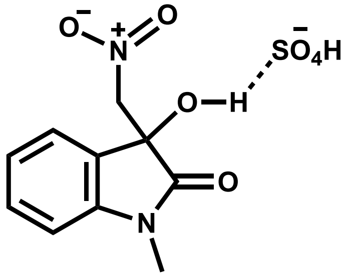

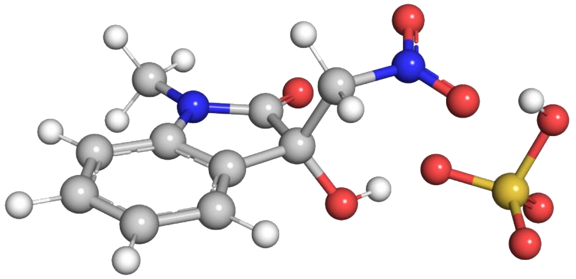

E (M06-2X)	*H (M06-2X)*	*G (M06-2X)*
-1495.577708	-1495.576764	-1495.646628

4 References

(1) Elinson, M. N.; Ilovaisky, A. I.; Merkulova, V. M.; Barba, F.; Batanero, B. Electrochemically Induced Henry Reaction of Nitromethane and Carbonyl Compounds. *Tetrahedron* **2008**, *64* (25), 5915–5919. https://doi.org/https://doi.org/10.1016/j.tet.2008.04.039.

(2) SaiPrathima, P.; Srinivas, K.; Mohan Rao, M. "On Water" Catalysis: An Expeditious Approach for the Synthesis of Quaternary Centered 3-Hydroxy-3-(Nitromethyl)Indolin-2-One Derivatives. *Green Chem.* **2015**, *17* (4), 2339–2343. https://doi.org/10.1039/C4GC02203C.

(3) SaiPrathima, P.; Srinivas, K.; Mohan Rao, M. "On Water" Catalysis: An Expeditious Approach for the Synthesis of Quaternary Centered 3-Hydroxy-3-(Nitromethyl)Indolin-2-One Derivatives. *Green Chem.* **2015**, *17* (4), 2339–2343. https://doi.org/10.1039/c4gc02203c.

(4) Stephenson, R. M. Mutual Solubilities: Water-Ketones, Water-Ethers, and Water-Gasoline-Alcohols. *J. Chem. Eng. Data* **1992**, *37* (1), 80–95. https://doi.org/10.1021/je00005a024.

(5) Gómez Marigliano, A. C.; Gramajo de Doz, M. B.; Sólimo, H. N. Influence of Temperature on the Liquid–Liquid Equilibria Containing Two Pairs of Partially Miscible Liquids. Water+nitromethane+1-Butanol Ternary System1The Material of This Paper Was Taken from a Thesis of Ana Clelia Gómez Marigliano Written in Partial F. *Fluid Phase Equilib.* **1998**, *149* (1), 309–322. https://doi.org/https://doi.org/10.1016/S0378-3812(98)00231-3.

(6) Vishwakarma, R.; Mannepalli, L. K.; Rathod, V. Kinetics of Henry Reaction Catalyzed by Fluorapatite. *Chem. Eng. Res. Des.* **2022**, *181*, 101–109. https://doi.org/10.1016/j.cherd.2022.03.001.

(7) Wu, C.; Hu, B.; Liu, H.; Jiang, J.; Kim, J. Arginine-Catalyzed Henry Reaction of α-Keto Amides with Nitromethane on Water. *ChemistrySelect* **2022**, *7* (7), e202104433. https://doi.org/10.1002/slct.202104433.

(8) Tanemura, K.; Suzuki, T. Base-Catalyzed Reactions Enhanced by Solid Acids: Amine-Catalyzed Nitroaldol (Henry) Reactions Enhanced by Silica Gel or Mesoporous Silica SBA-15. *Tetrahedron Lett.* **2018**, *59* (4), 392–396. https://doi.org/10.1016/j.tetlet.2017.12.050.

(9) Stewart, J. *MOPAC2016*; Stewart Computational Chemistry: Colorado Springs, 2016.

(10) Frisch, M. J.; Trucks, G. W.; Schlegel, H. B.; Scuseria, G. E.; Robb, M. A.; Cheeseman, J. R.; Scalmani, G.; Barone, V.; Petersson, G. A.; Nakatsuji, H.; Li, X.; Caricato, M.; Marenich, A.; Bloino, J.; Janesko, B. G.; Gomperts, R.; Mennucci, B.; Hratchian, H. P.; Ortiz, J. V.; Izmaylov, A. F.; Sonnenberg, J. L.; Williams-Young, D.; Ding, F.; Lipparini, F.; Egidi, F.; Goings, J.; Peng, B.; Petrone, A.; Henderson, T.;

Ranasinghe, D.; Zakrzewski, V. G.; Gao, J.; Rega, N.; Zheng, G.; Liang, W.; Hada, M.; Ehara, M.; Toyota, K.; Fukuda, R.; Hasegawa, J.; Ishida, M.; Nakajima, T.; Honda, Y.; Kitao, O.; Nakai, H.; Vreven, T.; Throssell, K.; Montgomery, J. A.; Jr., J. E. P.; Ogliaro, F.; Bearpark, M.; Heyd, J. J.; Brothers, E.; Kudin, K. N.; Staroverov, V. N.; Keith, T.; Kobayashi, R.; Normand, J.; Raghavachari, K.; Rendell, A.; Burant, J. C.; Iyengar, S. S.; Tomasi, J.; Cossi, M.; Millam, J. M.; Klene, M.; Adamo, C.; Cammi, R.; Ochterski, J. W.; Martin, R. L.; Morokuma, K.; Farkas, O.; Foresman, J. B.; Fox, D. J. Gaussian 09. Gaussian, Inc.: Wallingford CT 2016.

(11) Jung, Y.; Marcus, R. A. On the Theory of Organic Catalysis "on Water." *J. Am. Chem. Soc.* **2007**, *129* (17), 5492–5502. https://doi.org/10.1021/ja068120f.

(12) Rideout, D. C.; Breslow, R. Hydrophobic Acceleration of Diels-Alder Reactions. *J. Am. Chem. Soc.* **1980**, *102* (26), 7816–7817. https://doi.org/10.1021/ja00546a048.

(13) Grimme, S.; Antony, J.; Ehrlich, S.; Krieg, H. A Consistent and Accurate Ab Initio Parametrization of Density Functional Dispersion Correction (DFT-D) for the 94 Elements H-Pu. *J. Chem. Phys.* **2010**, *132* (15), 154104. https://doi.org/10.1063/1.3382344.
